# Phylogenetic footprint of the plant clock system in angiosperms: evolutionary processes of *Pseudo-Response Regulator*s

**DOI:** 10.1186/1471-2148-10-126

**Published:** 2010-05-01

**Authors:** Naoki Takata, Shigeru Saito, Claire Tanaka Saito, Matsuo Uemura

**Affiliations:** 1 United Graduate School of Agricultural Sciences, Iwate University, Morioka 020-8550, Japan; 2 Laboratory of Bioscience, Faculty of Engineering, Iwate University, Morioka 020-8551, Japan; 3 Cryobiofrontier Research Center, Iwate University, Morioka 020-8550, Japan; 4 Umeå Plant Science Centre, Department of Plant Physiology, Umeå University, SE-901 87 Umeå, Sweden; 5 Department of Bioenvironmental Science, Okazaki Institute for Integrative Bioscience, National Institutes of Natural Sciences, Okazaki 444-8787, Japan

## Abstract

**Background:**

Plant circadian clocks regulate many photoperiodic and diurnal responses that are conserved among plant species. The plant circadian clock system has been uncovered in the model plant, *Arabidopsis thaliana*, using genetics and systems biology approaches. However, it is still not clear how the clock system had been organized in the evolutionary history of plants. We recently revealed the molecular phylogeny of *LHY/CCA1 *genes, one of the essential components of the clock system. The aims of this study are to reconstruct the phylogenetic relationships of angiosperm clock-associated *PRR *genes, the partner of the *LHY/CCA1 *genes, and to clarify the evolutionary history of the plant clock system in angiosperm lineages.

**Results:**

In the present study, to investigate the molecular phylogeny of *PRR *genes, we performed two approaches: reconstruction of phylogenetic trees and examination of syntenic relationships. Phylogenetic analyses revealed that *PRR *genes had diverged into three clades prior to the speciation of monocots and eudicots. Furthermore, copy numbers of *PRR *genes have been independently increased in monocots and eudicots as a result of ancient chromosomal duplication events.

**Conclusions:**

Based on the molecular phylogenies of both *PRR *genes and *LHY/CCA1 *genes, we inferred the evolutionary process of the plant clock system in angiosperms. This scenario provides evolutionary information that a common ancestor of monocots and eudicots had retained the basic components required for reconstructing a clock system and that the plant circadian clock may have become a more elaborate mechanism after the speciation of monocots and eudicots because of the gene expansion that resulted from polyploidy events.

## Background

Many organisms such as cyanobacteria, fruit flies, mammals and plants have an endogenous time-keeping mechanism, a circadian clock, to gauge daily and seasonal environmental changes. Circadian clock systems in plants regulate various photoperiodic and diurnal responses, such as photomorphogenic processes, floral transition, leaf movements, stomatal conductance, photosynthetic capacity, and volatile emissions (reviewed in [1]). Among these, means to discriminate the length of the photoperiod are conserved among plant species, and it is commonly thought that circadian clock system of plants shares a basic mechanism that controls photoperiodic responses.

**Figure 1:**
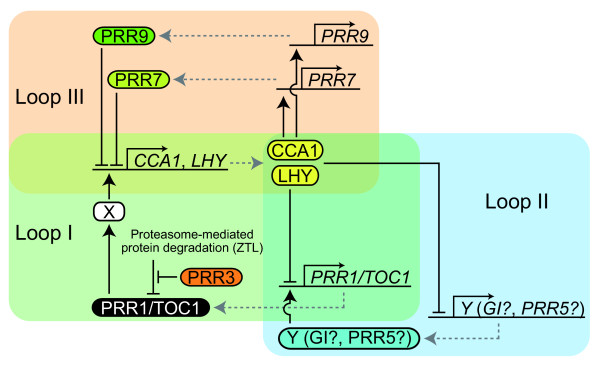
**Model of the circadian clock system in *Arabidopsis thaliana***. Green, blue and orange shadings indicate loop I, II and III, respectively. This figure is modified from McClung [7] and Harmer [60].

In the past decade, numerous molecular genetic analyses of the model plant *Arabidopsis thaliana *have uncovered the basic molecular network of the plant circadian clock (reviewed in [2,3]). Mathematical analyses have been used to develop a computational model of the plant clock system, which contains the main transcriptional feedback loop (Loop I) and two additional loops (Loops II and III) associated with the main loop (Figure 1) [4,5]. This multiple feedback loop system of the plant clock system is composed of two gene families, *Pseudo-Response Regulator*s (*PRR*s) and *Late Elongated Hypocotyl*/*Circadian Clock Associated 1 *(*LHY/CCA1*), and two unknown factors ("X" and "Y"). The main feedback loop (Loop I) consists of two *LHY/CCA1 *genes, the *Pseudo-Response Regulator 1*/*Timing of CAB2 Expression 1 *(*PRR1*/*TOC1*) gene and the unknown factor "X". In this loop, the feedback regulatory network operates as follows: the evening-acting *PRR1*/*TOC1 *gene induces the morning-acting *LHY *and *CCA1 *genes via the unknown factor "X", and is in turn repressed by *LHY/CCA1 *[6]. The Loop I associates with Loop II via the *PRR1/TOC1 *gene and with Loop III via *LHY/CCA1 *genes [4,5]. Loop II is made up of *PRR1/TOC1 *and an unknown factor "Y". It has been proposed that the unknown factor "Y" is *GIGANTEA *and/or *PRR5*, although the true component has not yet been identified [7]. Loop III consists of *LHY/CCA1 *genes and two *PRR *genes, *PRR7 *and *PRR9*. Together, the gene families *PRR*s and *LHY/CCA1*s have key roles and form the complex regulatory network in the plant clock system.

Clock-associated *PRR *genes are conserved among angiosperm evolutionary lineages as are their partner *LHY/CCA1 *genes [8,9]. In eudicotyledonous plants, five copies of *PRR *genes have been identified in *A. thaliana *and *Carica papaya *and seven copies have been found in *Populus trichocarpa *[10-12]. In monocotyledonous plants, *Oryza sativa *has five *PRR *genes [13]. The expression patterns of *PRR *genes in *A. thaliana *and *O. sativa *share some common features. The five *PRR *genes in *A. thaliana *show diurnal and sequential-temporal expression patterns from dawn to dusk as follows; *PRR9*→*PRR7*→*PRR5*→*PRR3*→*PRR1 *[10]. The similar sequential expression pattern is found in homologous genes of *O. sativa*, which are expressed as follows; *OsPRR73 *(*OsPRR37*)→*OsPRR95 *(*OsPRR59*)→*OsPRR1 *[13]. In spite of these similarities in the copy numbers and the expression patterns of clock-associated *PRR *genes, it is still unclear how the *PRR *genes have evolved in monocots and eudicots and how they have been incorporated in the regulatory network of the clock system in the evolutionary history of plants.

Rapid accumulation of genomic sequence data offers new perspectives on the molecular phylogeny of genes in angiosperms [14]. Completion of genomic sequences for various plant species reveals that angiosperm genomes have undergone several ancient chromosomal or whole genome duplication events [11,15-17]. In monocot lineages, the ρ polyploidy event occurred before the speciation of *O. sativa *and *Sorghum bicolor *in commelinids [18,19]. On the other hand, four polyploidy events appear to have occurred in eudicot lineages. Among these polyploidy events, the γ triplication event took place near the base of the eudicot clade though the timing of this event is still being debated [11,14,16,17]. The draft genomic sequence analysis of *C. papaya *has revealed that the genome of *A. thaliana *underwent two polyploidy events (α and β) after the speciation of *C. papaya *and *A. thaliana *in eurosids II [11]. Furthermore, the β polyploidy event is thought to have occurred before the α event [14]. In eurosids I, the salicoid polyploidy event occurred within the Salicaceae lineages, which includes *Populus *[17]. The footprints of these chromosomal duplication events are the conserved order of the genes on the duplicated chromosomes in the present genomic sequences [20]. Thus, comparison of the order of genes surrounding duplicated genes provides molecular evolutionary information on their phylogenetic relationships [21,22].

In the present study, to clarify the phylogenetic relationships among angiosperm *PRR *genes, we (1) identified *PRR *genes using available genomic databases of eudicots (*Vitis vinifera*, *P. trichocarpa*, *C. papaya*, and *A. thaliana*) and monocots (*O. sativa *and *S. bicolor*) and (2) examined the evolutionary processes of angiosperm *PRR *genes by conventional phylogenetic reconstruction and examination of syntenic relationships. With these results, we reconstructed the molecular phylogeny of *PRR *genes in angiosperms and found that gene expansion of *PRR*s occurred via polyploidy events in monocots and eudicots. Taken together with the molecular phylogeny of the other major gene family of the plant clock system (*LHY/CCA1*s) [9], our data allow us to explore the evolutionary history of the multiple feedback loop system in angiosperm lineages.

## Results

### Identification of clock-associated *PRR *genes in angiosperms

There are five copies of the *PRR *genes in the genomes of *O. sativa*, *S. bicolor*, *V. vinifera *and *C. papaya*, six copies in *A. thaliana*, and eight copies in *P. trichocarpa *(see Additional files 1 and 2). The *PRR1/TOC1 *gene in *C. papaya *was not retrieved from the genomic sequence database because the nucleotide sequence of the C-terminal region of the gene has not yet been determined. The angiosperm *PRR *genes retained a highly conserved PR-domain at the N-terminus and a CCT-motif at the C-terminus (see Additional file 3). However, two *PRR*-like genes (*PRR9b *in *A. thaliana*, *AtPRR9b*, and *PRR5c *in *P. trichocarpa*, *PtPRR5c*) retained the CCT-motif but not the PR-domain (see Additional file 4). Thus, we examined the molecular phylogeny of *PRR *genes but excluded *AtPRR9b *and *PtPRR5c *in the present study.

### Phylogenetic analysis of *PRR *gene family

**Figure 2:**
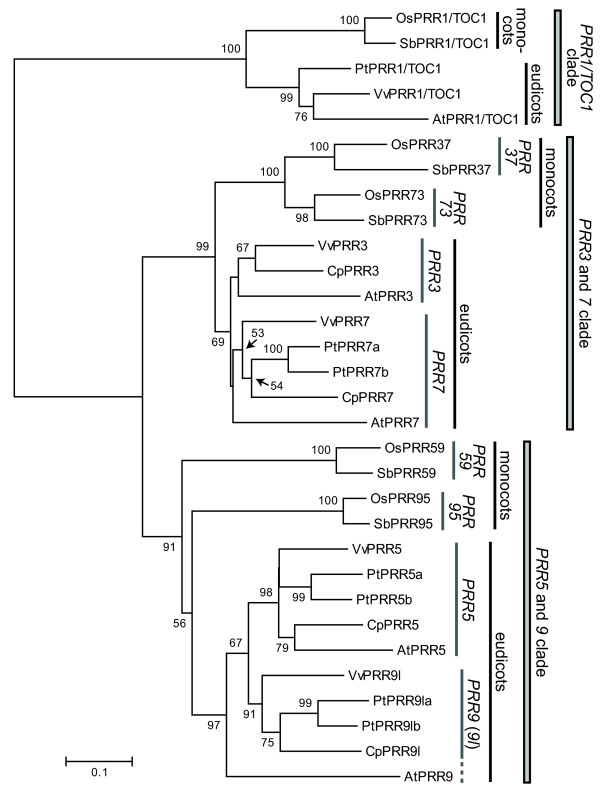
**Phylogenetic tree of angiosperm *PRR *genes**. Amino acid sequences were aligned using TCoffee program [47]. The phylogenetic tree was reconstructed by the ME method from the numbers of amino acid substitutions estimated by the JTT model. *PRR1/TOC1 *genes were utilized as an outgroup in the phylogenetic trees. The numerals at the branch indicate bootstrap values calculated by the ME method with 1,000 replications. Bootstrap values >50% are shown.

**Figure 3:**
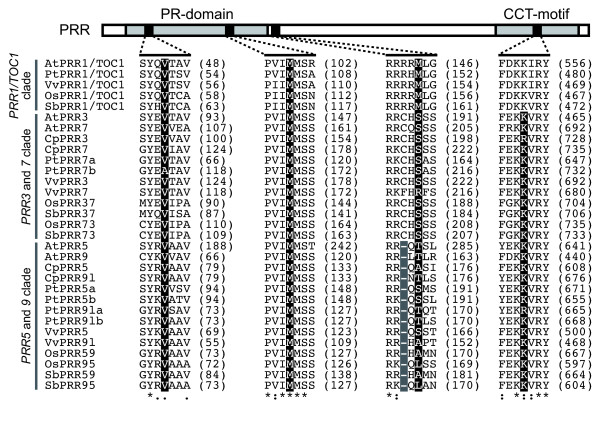
**Comparison of the exon-intron structures of *PRR *genes around the region of PR-domain and CCT-motif**. The amino acid sequences encoded by *PRR *genes were aligned using TCoffee program [47]. Accession numbers and gene IDs of the *PRR *genes are shown in Additional file 1. The numerals on the right side indicate the numbers of amino acid residues of each protein. Sequence similarity is indicated below the alignment using the symbols "asterisk," "colon," and "dot" for identical, highly similar, and weakly similar residues, respectively. Black and gray shadings on the alignments indicate a site of exon-intron boundary and one-amino acid deletion, respectively.

To deduce the evolutionary relationships among *PRR *genes in angiosperms, a phylogenetic tree was reconstructed using the minimum evolution (ME) method. Angiosperm *PRR *genes clearly separated into three clades (*PRR1/TOC1 *clade, *PRR3 *and *7 *clade, and *PRR5 *and *9 *clade) (Figure 2). This classification was consistent with the categorization of genomic structures of *PRR *genes that was apparent when exon-intron structures and insertions/deletions variation were examined (Figure 3, see Additional file 5). We found that each clade contains genes from both eudicots and monocots, suggesting that ancient *PRR *gene(s) diverged into three clades before the speciation of monocots and eudicots.

In all plant species examined, one copy of the *PRR1/TOC1 *gene was retained in the *PRR1/TOC1 *clade (Figure 2), whereas at least two copies were found in the *PRR3 *and *7 *clade and the *PRR5 *and *9 *clade. *PRR1/TOC1 *genes diverged into two clusters representing monocots and eudicots.

The *PRR3 *and *7 *clade consisted of two different clusters, each of which exclusively consists of monocot or eudicot genes (Figure 2). Accordingly, the phylogenetic tree suggested that the gene duplication events producing monocotyledonous *PRR37 *and *PRR73 *or eudicotyledonous *PRR3 *and *PRR7 *occurred independently within monocot and eudicot lineages, respectively. After the gene duplication event in eudicots, orthologs of *P. trichocarpa PRR3 *appeared to be lost, whereas the *P. trichocarpa PRR7 *gene was duplicated into *PRR7a *and *7b*.

In the *PRR5 *and *9 *clade, the monocot *PRR59 *and *PRR95 *genes showed an earlier gene duplication that may have occurred in a common ancestor of monocots and eudicots (Figure 2). However, the bootstrap value supporting this branch was not very high, 56%. Eudicotyledonous *PRR5 *and *PRR9*/*9*-like (*9l*) genes formed a cluster in the phylogenetic tree. In this cluster, *A. thaliana PRR9 *was distantly related to other *PRR5 *and *PRR9l *genes, which was also observed in the phylogenetic trees reconstructed by the neighbor-joining, maximum likelihood and Bayesian methods (see Additional files 6 and 7). This topology within *PRR5 *and *9 *clade might be the artefact caused by faster substitution rate of the *AtPRR9 *gene. Otherwise, sparse taxonomic sampling obscures the additional gene duplication and loss events occurred in eudicots. *PRR5a *in *P. trichocarpa *was more closely related to *PRR5b *than other *PRR5*, and similar close relationship was found between *PRR9la *and *PRR9lb *in *P. trichocarpa *(Figure 2). These findings indicated that the gene duplication events that produced *PRR5a *and *5b *and *PRR9la *and *9lb *occurred within rosids. Collectively, although the *PRR3 *and *7 *clade and the *PRR5 *and *9 *clade contained at least two copies of *PRR *genes in both monocots and eudicots, *PRR*s in the two clades are assumed to have independently duplicated in monocot and eudicot lineages.

### Functional divergence among *PRR *gene clusters

**Figure 4:**
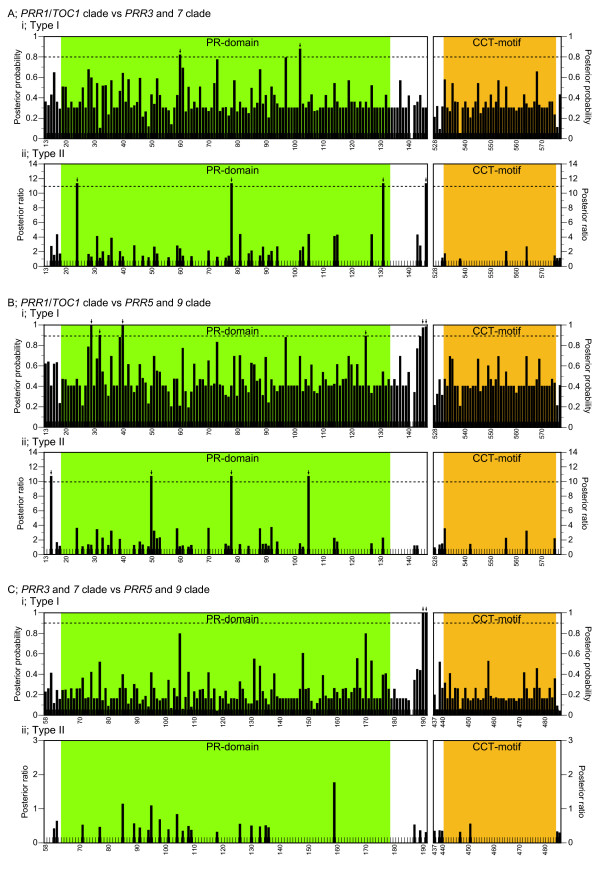
**Type I and type II functional divergences among the *PRR *gene clades**. Coefficients of type I (i) and type II (ii) functional divergences between *PRR1*/*TOC1 *clade and *PRR3 *and *7 *clade (A), between *PRR1*/*TOC1 *clade and *PRR5 *and *9 *clade (B) and between *PRR3 *and *7 *clade and *PRR5 *and *9 *clade (C) were calculated by DIVERGE 2.0 [55,56] using the TCoffee alignment (see Additional file 3) and the ME tree (Figure 2). Right and left panels indicate the PR-domain and its flanking region, and the CCT-motif and its flanking region, respectively. Positions of amino acid residues correspond to AtPRR1/TOC1 in *PRR1*/*TOC1 *clade vs *PRR3 *and *7 *clade, AtPRR1/TOC1 in *PRR1*/*TOC1 *clade vs *PRR5 *and *9 *clade and to AtPRR3 in *PRR3 *and *7 *clade vs *PRR5 *and *9 *clade. Cutoff values of the posterior probability and posterior ratio were established empirically by sequentially removing the highest scoring sites from the alignment until θ = 0. The cutoff values are shown by broken lines. The value of θ_II _between *PRR3 *and *7 *clade and *PRR5 *and *9 *clade was not set because the coefficient of the θ_II _was not significantly greater than zero. Thus, there is no broken line shown in the bottom panel (C, ii). The regions represented in this figure are surrounded with blue boxes in Additional file 3. Arrows indicate sites above the empirical cutoff values. Green and yellow shadings on the panels indicate PR-domain and CCT-motif, respectively.

**Figure 5:**
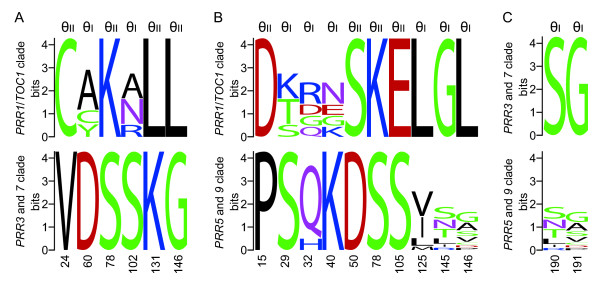
**Functionally divergent sites among the *PRR *gene clades**. Sequence logos indicate amino acid variation of type I (θ_I_) and type II (θ_II_) sites that were above the empirical cutoff values in the comparison between *PRR1*/*TOC1 *clade and *PRR3 *and *7 *clade (A), between *PRR1*/*TOC1 *clade and *PRR5 *and *9 *clade (B) and between *PRR3 *and *7 *clade and *PRR5 *and *9 *clade (C). Amino acids are color-coded by physicochemical property. Positions of amino acid residues (bottom) correspond to AtPRR1/TOC1 in *PRR1*/*TOC1 *clade vs *PRR3 *and *7 *clade, AtPRR1/TOC1 in *PRR1*/*TOC1 *clade vs *PRR5 *and *9 *clade and to AtPRR3 in *PRR3 *and *7 *clade vs *PRR5 *and *9 *clade. The regions represented in this figure are surrounded with blue boxes in Additional file 3. Sequence logos were generated with WebLogo version 2.8.2 [57,58].

Clock associated-*PRR *genes were divided into the three gene clusters (*PRR1/TOC1 *clade, *PRR3 *and *7 *clade, and *PRR5 *and *9 *clade) that had been formed prior to the speciation of monocots and eudicots. Although the amino acid sequences of the genes were highly conserved in the PR-domain and CCT-motif, there were several amino acid changes that were distinctive among the three clades, which potentially contribute to functional differences (see Additional file 3). To detect amino acid substitutions that are potentially involved in functional divergence among the clades in PR-domain, CCT-motif and their flanking regions, we performed statistical analysis to estimate the coefficient of type I and type II functional divergences (θ_I _and θ_II_). In the type I functional divergence, sites are conserved in one gene cluster but variable in the sister clusters [23]. On the other hand, type II sites are fixed in both clusters but the amino acid residues are different between the clusters [24]. In the comparison among the *PRR *gene clusters, all of the coefficients for the type I functional divergence (θ_I_) were significantly larger than zero (Table 1). In addition, the values of the type II functional divergence (θ_II_) between *PRR1*/*TOC1 *clade and *PRR3 *and *7 *clade and between *PRR1*/*TOC1 *clade and *PRR5 *and *9 *clade were significantly different from zero while the value between *PRR3 *and *7 *clade and *PRR5 *and *9 *clade was not significantly greater than zero. Six sites that were above the empirical cutoff values were identified in the comparison between *PRR1/TOC1 *clade and *PRR3 *and *7 *clade, ten sites in *PRR1*/*TOC1 *clade and *PRR5 *and *9 *clade and two sites in *PRR3 *and *7 *clade and *PRR5 *and *9 *clade (Figures 4 and 5). Intriguingly, these sites were predominantly detected in the PR-domain and its flanking region rather than the CCT-motif (Figure 4). These results imply that the amino acid substitutions in the PR-domain, through which PRR proteins interact with other proteins (ZEITLUPE and PRRs) [25,26], may partially contribute to the functional divergence among the three gene clusters.

**Table 1:** Coefficients of type I and type II functional divergences among the *PRR *gene clades.

Comparison	Type I		Type II	
		
	θ_I_	SE	θ_II_	SE
PRR1/TOC1 vs PRR3 and 7	0.366	0.109*	0.196	0.093**
PRR1/TOC1 vs PRR5 and 9	0.473	0.075*	0.175	0.088**
PRR3 and 7 vs PRR5 and 9	0.234	0.048*	-0.003	0.106

*P < 0.01, **P < 0.05

### Phylogenetic relationships of *PRR *gene family inferred from chromosome syntenies

To clarify evolutionary events such as gene duplication and gene deletion among angiosperm *PRR *genes, we investigated chromosomal syntenies among the genomes of monocots or eudicots. Because ancient chromosome duplication events result in conserved gene order on the duplicated chromosomes [20], comparisons of gene organization and detection of chromosomal syntenies can provide molecular evolutionary information to understand the phylogenetic relationships of the genes [21,22].

**Figure 6:**
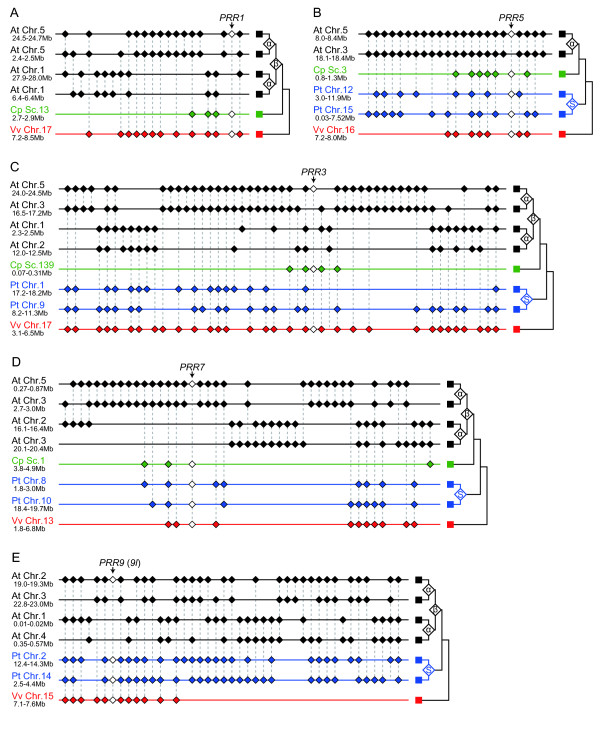
**Chromosomal syntenies of flanking regions of eudicotyledonous *PRR *genes**. Syntenic relationships of flanking regions of *PRR1/TOC1 *(A), *PRR5 *(B), *PRR3 *(C), *PRR7 *(D) and *PRR9*/*9l *(E) were examined using the comparative genomic tool, CoGe [29]. Syntenic relationships within *A. thaliana *or *P. trichocarpa *were analyzed by a comparative genomic tool, CoGe, and according to previous studies [17,27,28,59]. Diamonds colored with black (*A. thaliana*), green (*C. papaya*), blue (*P. trichocarpa*) and red (*V. vinifera*) indicate individual genes. White diamonds marked with arrows indicate *PRR *genes. Genes with no syntenic matches on the selected regions are not plotted. Orthologous genes are connected by broken lines. Diamonds with characters on the right side of strands indicate angiosperm polyploidy event (α, β and salicoid). The salicoid polyploidy event is shown as the diamond with the initial letter of salicoid (S). The lengths of the genomic regions are shown on the left.

In eudicots, the flanking region of *PRR1/TOC1*, *PRR3*, *5*, *7 *and *9 *in *A. thaliana *showed a syntenic relationship with *PRR1/TOC1*, *PRR3*, *5*, *7 *and *9l *in *V. vinifera*, *P. trichocarpa*, and *C. papaya*, respectively (Figure 6). In addition, the syntenies were also found in the flanking regions between *AtPRR3 *and *VvPRR7*, *AtPRR7 *and *VvPRR3*, *AtPRR5 *and *VvPRR9l *and *AtPRR9 *and *VvPRR5 *(data not shown). The former syntenic relationships between *AtPRR3 *and *VvPRR3*, *AtPRR7 *and *VvPRR7*, *AtPRR5 *and *VvPRR5 *and *AtPRR9 *and *VvPRR9l *were more conserved than the latter relationships between *AtPRR3 *and *VvPRR7*, *AtPRR7 *and *VvPRR3*, *AtPRR5 *and *VvPRR9l *and *AtPRR9 *and *VvPRR5*, respectively. Syntenic relationships were not found between the neighbouring regions of *P. trichocarpa PRR1/TOC1 *and those of other *PRR1/TOC1*s, or between the neighbouring region of *C. papaya PRR9l *and those of other *PRR9*/*9l*s. It is not clear whether these observations can be ascribed to chromosomal rearrangements or fragmentations of genomic sequences in small contigs.

In the genome of *A. thaliana*, the flanking regions of *PRR *genes showed syntenies with one or three partial regions of its genome (Figure 6). These syntenic relationships originated from the chromosomal duplications that arose from the β and α polyploidy events [27,28]. In the *P. trichocarpa *genome, two copies of each *PRR5*, *PRR7*, and *PRR9l *gene were located at the syntenic regions of chromosomes 12 and 15 (Figure 6B), those of chromosomes 8 and 10 (Figure 6D), and those of chromosomes 2 and 14 (Figure 6E), respectively. Tuskan *et al*., [17] showed that these syntenic regions were produced via the salicoid polyploidy event. Although flanking region of *A. thaliana PRR3 *shares syntenic relationships with partial regions of *P. trichocarpa *chromosomes 1 and 9, these two partial regions did not retain a *PRR *gene (Figure 6C).

**Figure 7:**
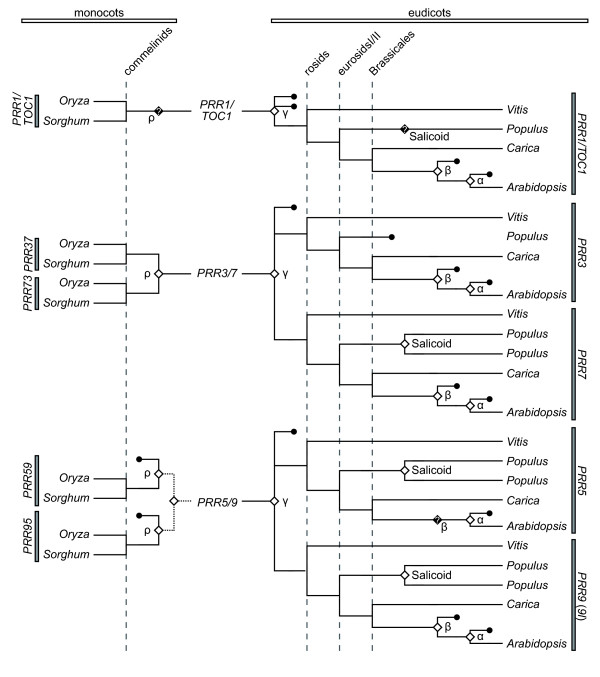
**Evolutionary processes of *PRR *genes in angiosperms reconstructed by phylogenetic analysis and syntenic relationships**. Chromosomal syntenies among eudicots or monocots were analyzed by the comparative genomic tool, CoGe [29] or VISTA Browser [31], and according to previous studies [11,16-18,27,28,30,59]. Diamonds and circles indicate gene duplication and gene loss event, respectively. The timing of a gene duplication event that is not clear in the previous studies is shown by dotted line. Black diamonds with a question mark indicate that a gene duplication event derived from a polyploidy event is not resolved. Timings of plant speciation in commelinids, rosids, eurosids I/II and Brassicales are described by broken lines.

Next, we investigated the chromosomal syntenic relationships derived from the γ triplication event using the genomic information of *V. vinifera *[16]. There were chromosomal syntenies conserved between the flanking regions of *VvPRR3 *and *VvPRR7 *and between the flanking regions of *VvPRR5 *and *VvPRR9l*, which were originated from the γ triplication event ([16], see also the comparative genomic tool, CoGe [29]). The synteny of *PRR3*, *5*, *7 *and *9l *in *V. vinifera *and homologous genes in other eudicotyledonous plants (*A. thaliana*, *P. trichocarpa*, and *C. papaya*), respectively, suggest that the ancestral *PRR3/7 *gene in eudicots was duplicated into *PRR3 *and *PRR7*, and *PRR5/9 *into *PRR5 *and *PRR9*/*9l *in the γ polyploidy event (Figure 7). After the γ polyploidy event, one copy of each *PRR *gene (*PRR1/TOC1*, *PRR3*, *5*, *7 *and *9*/*9l*) has been conserved in the present genomes of *V. vinifera *and *C. papaya*, which apparently have not undergone additional polyploidy events. Although *A. thaliana PRR *genes were repeatedly duplicated by the β and/or α polyploidy events, one copy of each gene remains in the present *A. thaliana *genome, which is similar to *V. vinifera *and *C. papaya *genomes (Figures 6 and 7). In the genome of *P. trichocarpa*, *PRR5*, *7*, and *9l *were duplicated in the subsequent salicoid polyploidy event, but the *PRR3 *was lost prior to the salicoid polyploidy event or duplicated *PRR3*s were lost following the polyploidy event.

The flanking region of the *PRR *gene in *O. sativa *(*OsPRR1/TOC1*, *OsPRR73*, *OsPRR59 *and *OsPRR95*) showed conserved synteny with that of orthologous genes in *S. bicolor *[30]. On the other hand, only a few syntenic regions were identified between *PRR37 *of *O. sativa *and the orthologous gene of *S. bicolor*. In the genome of *O. sativa*, the neighbouring region of *OsPRR37 *showed synteny with that of *OsPRR73*. This syntenic relationship resulted from the chromosomal duplication that occurred in the ρ polyploidy event [18]. The ρ polyploidy event also resulted in conserved chromosomal synteny between the flanking region of *OsPRR59 *and a partial region of chromosome 8, and between the flanking region of *OsPRR95 *and a different partial region of chromosome 8. However, these partial regions of chromosome 8 have lost *PRR *genes ([18], see also the comparative genomic tool, VISTA Browser [31]). These results indicated that the gene duplication event resulting in *PRR37 *and *PRR73 *was the monocotyledonous ρ polyploidy event, and that *PRR59 *and *PRR95 *were duplicated via the ρ polyploidy event but one of the duplicated genes was lost from genomes of *O. sativa *and *S. bicolor *(Figure 7).

## Discussion

The plant clock system consists of multiple interlocked feedback loops, which are comprised predominantly of two gene families, *LHY/CCA1*s and *PRR*s [4,5]. These gene families are conserved among both monocots and eudicots [8]. To clarify the evolutionary process of the plant clock system, we recently reported the molecular phylogeny of *LHY/CCA1 *genes in angiosperms [9]. Furthermore, in the present study, we reconstructed phylogenetic relationships among clock-associated *PRR *genes in monocots and eudicots using two approaches: reconstruction of phylogenetic trees and examination of syntenic relationships. Together, these phylogenetic analyses of the plant circadian clock related-genes, *LHY/CCA1*s and *PRR*s, are promising tools to unravel the evolutionary history of the plant clock system among angiosperm lineages.

### Evolutionary processes of clock-associated *PRR *genes in angiosperms

*PRR *genes are conserved in angiosperms and at least five copies of *PRR *genes have been retained in their genomes (see Additional file 1). Angiosperm *PRR *genes are grouped into three clades (the *PRR1/TOC1 *clade, the *PRR3 *and *7 *clade, and the *PRR5 *and *9 *clade) that have already existed prior to the divergence of monocots and eudicots (Figures 2 and 3). After the speciation of monocots and eudicots, copy numbers of *PRR *genes independently increased in each lineage as a result of ancient chromosomal duplication events (Figure 7). In monocots, the ancestral *PRR37*/*PRR73 *was duplicated into *PRR37 *and *PRR73 *in the ρ polyploidy event that occurred before the speciation of *O. sativa *and *S. bicolor *[18,19]. In eudicots, the gene duplication events between *PRR3 *and *PRR7 *and between *PRR5 *and *PRR9*/*9l *are derived from the γ polyploidy event that took place before the speciation of Vitales (*V. vinifera*) and eurosid species (*P. trichocarpa*, *C. papaya*, and *A. thaliana*) [16]. In addition, our results show that *PRR *genes in *P. trichocarpa *have expanded more than those in other plant species (see Additional file 1). This expansion apparently resulted from the salicoid polyploidy event that occurred in the *Populus *lineage but not in other eudicots (*V. vinifera*, *C. papaya*, and *A. thaliana*) (Figure 7). Consequently, circadian clocks may have become more intricate networks after the speciation of monocots and eudicots if the additional genes have roles in the circadian networks.

In contrast to the increase in *PRR *genes via ancient chromosomal duplication events, the present genome of *A. thaliana *retains one copy of each *PRR *gene (*PRR1/TOC1*, *PRR3*, *5*, *7*, and *9*) after β and α polyploidy events (Figure 7). Likewise, in *Brassica rapa*, *PRR *genes that were increased in a recent hexaploidization event have been reduced in its genome following the event, though the genome retains at least a set of *PRR *genes [32]. These results implied that dosages of the clock related-genes had been altered in the genomes during evolution. Since some reports showed that a gene dosage change of clock related genes influenced the clock regulatory network and downstream signals [33,34], plants may have flexibly modulated the complex network of the clock system after polyploidy events and subsequent gene deletion events. Indeed, allopolyploid *Arabidopsis *species can fine-tune a regulatory and stoichiometric balance of the circadian clock system to properly maintain a downstream homeostasis of the plants [35]. The history of gene duplications and deletions in the *Arabidopsis *clock system imply that the regulatory network of the clock system has maintained a degree of organization throughout the dynamic changes of copy numbers and functions of clock-related genes.

### Phylogenetic footprint of the plant clock system in angiosperms

**Figure 8:**
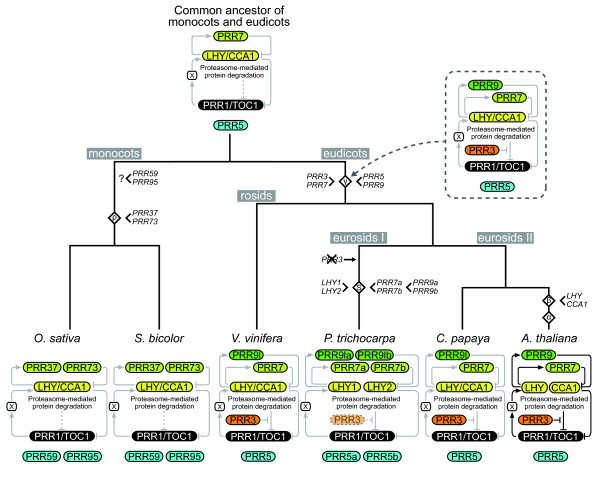
**Proposed schematic diagram of the evolutionary process of the plant circadian clock system**. For the plant clock system model, loop I and III are described in this diagram. Diamonds with characters indicate angiosperm polyploidy event (α, β, γ, salicoid and ρ). The salicoid polyploidy event is shown as the diamond with the initial letter of salicoid (S).

Loops I and III of the *Arabidopsis *clock system contain four *PRR *genes (*PRR1/TOC1*, *PRR3*, *7 *and *9*) and two *LHY/CCA1 *genes (*LHY *and *CCA1*) (Figure 1) [4,5,36]. Although the circadian clock-related genes in *A. thaliana *were duplicated via the β and α polyploidy events, the present genome of *A. thaliana *retains only one pair of *LHY *and *CCA1 *genes, which is derived from the β polyploidy event (Figure 8) [9]. As the β polyploidy event is assumed to have occurred in the Brassicaceae, *LHY *and *CCA1 *genes did not diverge before the speciation of *A. thaliana *and *C. papaya*, which is consistent with the fact that there is only one copy of the *LHY/CCA1 *gene in the genome of *C. papaya *[11]. Similar to the genome of *A. thaliana*, the *C. papaya *genome retained only one copy each of the *PRR1/TOC1*, *PRR3*, *7*, and *9 *genes (see Additional file 1). These results suggest that one copy of *LHY/CCA1*, *PRR1/TOC1*, *PRR3*, *7*, and *9 *was involved in the plant clock system in the common ancestor of *A. thaliana *and *C. papaya*.

The evolutionary history of the plant clock system in two divergent members of Brassicales (*A. thaliana *and *C. papaya*) raises the question as to when the *Arabidopsis*-type clock apparatus arose in the evolutionary history of plants. Phylogenies of the circadian clock-related genes showed that a set of the genes, one copy of each *LHY/CCA1*, *PRR1/TOC1*, *PRR3*, *7 *and *9l *genes, is conserved in the genome of *V. vinifera *(Figures 7 and 8, see Additional file 1) [9,16]. Conservation of the set of clock-related genes suggests that the fundamental mechanism of the *Arabidopsis*-type clock apparatus was formed before the speciation of Vitales (*V. vinifera*) and eurosid species (*P. trichocarpa*, *C. papaya*, and *A. thaliana*), although it remains to be determined whether functional divergences between *PRR3 *and *7 *and between *PRR5 *and *9*/*9l*, which were duplicated in the γ triplication event, existed in the common ancestor of Vitales and eurosids (Figure 8).

Intriguingly, the clock system of *P. trichocarpa *might differ from the *Arabidopsis *clock system, because the *P. trichocarpa PRR3 *gene was lost and *LHY/CCA1 *and *PRR7 *and *9 *were duplicated via the salicoid polyploidy event that occurred after the speciation of eurosids I and II (Figures 7 and 8). PRR3 protein in *A. thaliana *interacts with PRR1/TOC1 protein, which is a component of the main loop (Loop I), to inhibit its protein degradation [36]. The lack of *PRR3 *in *Populus *might influence the posttranslational regulation of PRR1/TOC1 protein or might be compensated by recruiting other *PRR *genes although conserved changes that would mark the *PRR3 *gene cluster were not identified in other *Populus *PRR proteins (data not shown). Furthermore, duplication of *LHY/CCA1 *and *PRR7 *and *9*, but not *PRR1/TOC1*, could also affect the regulation mechanism of the *Populus *clock system, because a dosage balance in the plant clock system was ruined [33]. We recently revealed that *Populus LHY*s show typical diurnal expressions similar to *LHY/CCA1 *genes in other plant species [9,37-40], which is apparently contradictory to the speculation. The *Populus *clock system appears to retain a functional regulatory network in regard to the expression regulation of the *LHY *genes throughout the evolutionary changes of the circadian clock components.

The monocots *O. sativa *and *S. bicolor *retain one *LHY/CCA1 *gene and five *PRR *genes in their genomes (see Additional file 1) [9]. Phylogenetic analysis showed that the gene duplication events that produced *PRR37 *and *73*, and *PRR59 *and *95 *in monocots occurred separately and independently of the events that produced *PRR3 *and *7*, and *PRR5 *and *9 *in eudicots (Figure 7). This finding raises a complex question; what are the roles of these paralogous genes in the monocotyledonous clock system? The expression of *PRR5 *in *A. thaliana *is not regulated by light signals and reaches a peak of the diurnal rhythm around 8 h after dawn [10]. On the other hand, *PRR9 *in *A. thaliana *shows a light response expression, resulting in rhythmicity with peak expression just after dawn [10,41]. *PRR59 *and *95 *in *O. sativa *have peak expressions around 9 h after dawn and are not induced by light signals [13], which appear to be more similar to the regulation of *A. thaliana PRR5 *expression than to that of *A. thaliana PRR9 *expression. In addition, peak expressions of *O. sativa PRR37 *and *73 *are followed by expressions of *PRP59 *and *95*, which may correspond to the sequential expression pattern of *A. thaliana PRR7 *and *PRR5 *[10,13]. These results collectively suggest that paralogous gene pairs *PRR59*/*95 *and *PRR37*/*73 *genes in monocots share functional roles with *PRR5 *and *PRR7*, respectively, in *A. thaliana *(Figure 8). Together, these data indicate that a common ancestor of monocots and eudicots may have had a main feedback loop (*LHY/CCA1*-*PRR1/TOC1*) that was not posttranslationally regulated by *PRR3*. Although the ancestral clock system appears to have been more simplified than that of the current *Arabidopsis*-type clock apparatus, it is assumed that the ancestral clock system have had the basic components reconstructing a primitive multiple feedback loop system.

## Conclusions

The present study inferred the molecular phylogeny of angiosperm *PRR *genes that have key roles in the plant clock system. Clock-associated *PRR *genes diverged into three clades before the speciation of monocots and eudicots and, in addition, *PRR3/7 *and *PRR5*/*9 *underwent independent expansion in monocots and eudicots (Figure 7). Taken together with the molecular phylogeny of *LHY/CCA1 *genes [9], our studies suggest that the basic components of the *Arabidopsis *clock were established prior to the speciation of eudicots and monocots (Figure 8). Additional functional analyses and accumulation of genomic information from other plant species will clarify details of evolutionary and developmental processes of plant clock systems.

## Methods

### Retrieval of sequences of clock-associated *PRR *genes from draft genome sequences

*PRR *genes were retrieved from genomic databases for *A. thaliana *(TIGR *Arabidopsis thaliana *Database [42]) and *O. sativa *(Rice Annotation Project Database [43]). To identify *PRR *genes in *S. bicolor*, *V. vinifera*, *P. trichocarpa*, and *C. papaya*, TBLASTN searches were performed against the genomic databases using amino acid sequences encoded by *PRR *genes of *A. thaliana *or *O. sativa *as queries: JGI *Sorghum bicolor *v1.0 [44] for *S. bicolor*; Grape Genome Browser [45] for *V. vinifera*; JGI *Populus trichocarpa *v1.1 [46] for *P. trichocarpa*, and Papaya Genome Project v0.4 in CoGe [29] for *C. papaya*. Genes that retained the typical PR-domain at the N-terminal region and the CCT-motif at the C-terminal region were retrieved from these genomic databases. Genes that lacked the PR-domain or CCT-motif but showed significantly high similarity with typical *PRR *genes were also retrieved (those with E-values lower than 10^-50 ^or >90% similarity). The genes retrieved from the genomic databases were aligned with *PRR *genes in *A. thaliana *and *O. sativa *using the TCoffee program [47]. Mispredicted genes, if found, were manually modified as follows. For predicted genes lacking a conserved portion of the *PRR *gene, we searched the database for expressed sequence tags (ESTs) of the gene (TIGR Plant Transcript Assemblies [48]) and re-annotated by assembling the predicted gene and relevant ESTs. In some cases, the open reading frame of the gene was repredicted by the Fgenesh+ program [49]. When the exon-intron boundary of a gene was mis-demarcated, we improved the boundary based on standard donor/acceptor splice sites without resulting in a frame shift.

### Phylogenetic analysis

Amino acid sequences were deduced from nucleotide sequences of the predicted *PRR *genes and then aligned using the TCoffee program [47]. The alignment around the flanking region of the PR-domain was manually corrected based on the exon-intron structure. The number of amino acids substituted between each pair of PRR proteins was estimated by the Jones-Taylor-Thornton (JTT) model [50] with the complete- deletion option. From the number of estimated amino acid substitutions, a phylogenetic tree was reconstructed by the ME method [51]. Bootstrap values were calculated with 1,000 replications using the ME method [52]. *PRR1/TOC1 *genes were utilized as an outgroup in the phylogenetic trees. These procedures were performed using the MEGA4 software [53,54].

### Detection of functional divergence

Type I and type II functional divergences among *PRR *gene clades was examined using the DIVERGE 2.0 software [24,55,56]. To calculate coefficient of type I and type II functional divergences (θ_I _and θ_II_), we used the protein sequence alignment constructed using the TCoffee program [47] and the topology and branch length of phylogenetic tree reconstructed by the ME method. Cutoff values of the posterior probability and posterior ratio were set empirically by sequentially removing the highest scoring sites from the alignment until θ = 0. Sequence logos were generated with WebLogo version 2.8.2 [57,58].

### Identification of chromosomal synteny

Conservation of chromosomal synteny in *V. vinifera*, *P. trichocarpa*, *C. papaya*, and *A. thaliana *was determined as follows. First, we reconstructed the ancient gene organization of the flanking regions of *A. thaliana PRR *genes before the α and β polyploidy events using the chromosomal syntenies reported in previous studies [27,28]. Then, we compared the syntenic relationships between the ancient gene organization in *A. thaliana *and the flanking regions of *PRR *genes in *V. vinifera*, *P. trichocarpa*, and *C. papaya *using the comparative genomic tool, CoGe [11,29,59]. This process also reconstructed chromosomal syntenies in *P. trichocarpa *that were derived from the salicoid polyploidy event [17]. To clarify syntenic relationships derived from the γ polyploidy event, we used information on chromosomal syntenies within the genome of *V. vinifera *[16] and the comparative genomic tool, CoGe [29].

Syntenic relationships between the flanking regions of *PRR *genes in *O. sativa *and those in *S. bicolor *were reconfirmed using the chromosomal syntenies reported in previous studies [30] and a comparative genomic tool VISTA Browser [31]. To reconstruct chromosomal syntenies of the flanking regions of *PRR *genes derived from the monocotyledonous ρ polyploidy event, syntenic regions were identified according to methods reported previously using the *O. sativa *genomic sequence [18].

## Authors' contributions

NT conceived of the study, performed all analyses and wrote the manuscript. MU, SS and CTS participated in coordination and helped to draft the manuscript. All authors read and approved the final manuscript.

## Supplementary Material

Additional file 1 ***PRR *genes in angiosperms used in the present study**. ^a^Plant classification refers to APGII [61]. ^b^Gene ID corresponds to the name obtained from Rice Annotation Project Database [43]. ^c^Gene ID corresponds to the name obtained from JGI *Sorghum bicolor *v1.0 [44]. ^d^Gene IDs correspond to the names obtained from TIGR Plant Transcript Assemblies [48]. ^e^Gene ID corresponds to the name obtained from Grape Genome Browser [45]. ^f^Gene IDs correspond to the names obtained from JGI *Populus trichocarpa *v1.1 [46]. ^g^Gene IDs correspond to the names obtained from TIGR Plant Genomics [62]. ^h^Gene IDs correspond to the names obtained from Comparative Genomics Homepage [29].*Genes, which appeared to be misprediced, were manually modified in this study. **Gene is misannotated.***Genes lack the PR-domain. Click here for file

Additional file 2 **Predicted *PRR *genes in angiosperms**. Angiosperm *PRR *genes were retrieved from genomic databases for *A. thaliana *(TIGR *Arabidopsis thaliana *Database, http://www.tigr.org/tdb/e2k1/ath1/), *V. vinifera *(Grape Genome Browser, http://www.genoscope.cns.fr/externe/GenomeBrowser/Vitis/), *P. trichocarpa *(JGI *Populus trichocarpa *v1.1, http://genome.jgi-psf.org/Poptr1_1/Poptr1_1.home.html), *C. papaya *(Papaya Genome Project v0.4 in CoGe, http://synteny.cnr.berkeley.edu/CoGe/), *O. sativa *(Rice Annotation Project Database, http://rapdb.dna.affrc.go.jp/) and *S. bicolor *(JGI *Sorghum bicolor *v1.0, http://genome.jgi-psf.org/Sorbi1/Sorbi1.home.html). Click here for file

Additional file 3 **Alignments of the amino acid sequences encoded by angiosperm *PRR *genes**. Amino acid sequences were aligned using TCoffee program http://www.ebi.ac.uk/t-coffee/. Amino acid conservation was highlighted by the boxshade program http://www.ch.embnet.org/software/BOX_form.html. Identical and similar amino acid residues are highlighted with black and gray shading, respectively. Blue boxes indicate the regions that were represented in Figures 4 and 5. Click here for file

Additional file 4 **Alignment of the amino acid sequences encoded by (A)*AtPRR9*s and (B)*PtPRR5*s**. Sequence similarity is indicated below the alignment using the symbols "asterisk," "colon," and "dot" for identical, highly similar, and weakly similar residues, respectively. Black shadings indicate the PR-domain and the CCT-motif. Accession numbers and gene IDs of the *PRR *genes are shown in Additional file 1. Click here for file

Additional file 5 **Nucleotide sequences around the region of the exon-intron boundaries of angiosperm *PRR *genes at the flanking region of PR-domain**. Black and gray shadings on the alignments indicate a site of exon-intron boundary and one-amino acid deletion, respectively. Higher degree of conservation of nucleotide sequence is shown by the bigger size of letters. Click here for file

Additional file 6 **Phylogenetic tree of *PRR *genes reconstructed by the Neighbor-Joining (NJ) method**. Full-length amino acid sequences were aligned using TCoffee program. The phylogenetic tree was reconstructed by the NJ method from the numbers of amino acid substitutions estimated by applying the JTT model. The numerals at the branch indicate bootstrap values calculated by the NJ method with 1,000 replications. Bootstrap values >50% are shown. Click here for file

Additional file 7 **Phylogenetic trees of *PRR *genes reconstructed by the Maximum likelihood (ML) and Bayesian methods**. Full-length amino acid sequences were aligned using TCoffee program. The phylogenetic trees were reconstructed by the ML and Bayesian methods with applying the JTT model. The ML and Bayesian analyses were performed using the PhyML http://www.atgc-montpellier.fr/ and MrBayes http://mrbayes.csit.fsu.edu/ programs, respectively. *PRR1/TOC1 *genes were utilized as an outgroup in the phylogenetic trees. Support for the branches was calculated as percent of 100 bootstrap replications of the ML method (left) and Baysian posterior probabilities (right). The values >50% are shown. Click here for file
