# Supplementary material for: Phylogenetic footprint of the plant clock system in angiosperms: evolutionary processes of Pseudo-Response Regulators
Source: BMC Evol Biol. 2010 May 1;10:126. doi: 10.1186/1471-2148-10-126 (PMC2887406; doi:10.1186/1471-2148-10-126)
Supplement: Additional file 2 — Predicted PRR genes in angiosperms. Angiosperm PRR genes were retrieved from genomic databases for A. thaliana (TIGR Arabidopsis thaliana Database, http://www.tigr.org/tdb/e2k1/ath1/), V. vinifera (Grape Genome Browser, http://www.genoscope.cns.fr/externe/GenomeBrowser/Vitis/), P. trichocarpa (JGI Populus trichocarpa v1.1, http://genome.jgi-psf.org/Poptr1_1/Poptr1_1.home.html), C. papaya (Papaya Genome Project v0.4 in CoGe, http://synteny.cnr.berkeley.edu/CoGe/), O. sativa (Rice Annotation Project Database, http://rapdb.dna.affrc.go.jp/) and S. bicolor (JGI Sorghum bicolor v1.0, http://genome.jgi-psf.org/Sorbi1/Sorbi1.home.html). [file 1471-2148-10-126-S2.RTF]

Additional file 2. Predicted PRR genes in angiosperms

Arabidopsis thaliana
>AtPRR1/TOC1
ATGGATTTGAACGGTGAGTGTAAAGGAGGAGATGGGTTTATTGATAGAAGCAGAGTCAGGATTTTGCTTTGTGACAATGATTCCACGAGTTTGGGAGAGGTTTTTACTCTCCTTTCAGAGTGTTCTTATCAAGTGACTGCAGTGAAATCAGCAAGGCAGGTGATTGATGCACTTAATGCAGAGGGACCTGATATCGATATAATACTGGCGGAAATTGATCTCCCAATGGCTAAGGGTATGAAGATGCTGAGGTACATCACACGAGACAAAGATCTTCGCAGAATCCCTGTGATAATGATGTCGAGGCAAGACGAAGTCCCTGTCGTTGTAAAGTGCTTGAAGCTAGGTGCAGCTGACTACCTTGTGAAGCCTCTTCGCACCAACGAGCTTCTGAACTTGTGGACACACATGTGGAGAAGAAGACGCATGCTAGGACTTGCTGAGAAGAATATGTTGAGCTATGATTTTGATCTTGTGGGATCTGATCAAAGTGATCCAAACACAAATAGTACCAACCTGTTCTCTGACGACACAGATGATAGAAGTCTTAGGTCCACCAACCCACAGAGAGGAAATTTAAGTCACCAGGAAAATGAGTGGTCTGTTGCTACTGCTCCTGTTCATGCTCGTGATGGTGGTCTTGGTGCTGATGGAACAGCCACTTCTTCTCTTGCTGTTACTGCTATAGAGCCTCCATTGGATCATCTTGCTGGGTCTCACCATGAGCCAATGAAAAGAAATAGTAATCCAGCGCAATTTTCTTCAGCACCGAAGAAAAGTAGATTGAAGATCGGAGAGTCCTCTGCTTTCTTTACATATGTCAAATCTACTGTCCTTAGAACTAACGGTCAGGATCCTCCTCTTGTCGATGGAAATGGCTCACTTCATCTTCATCGGGGTTTGGCGGAGAAGTTTCAAGTGGTGGCTAGTGAAGGGATCAACAACACCAAACAAGCACGCAGAGCAACACCAAAATCTACTGTCCTTAGAACTAACGGTCAGGATCCTCCTCTTGTCAATGGAAATGGCTCACATCATCTTCATCGGGGTGCGGCGGAAAAGTTTCAAGTGGTGGCTAGTGAAGGGATCAACAACACCAAACAAGCACACAGAAGTAGAGGGACCGAGCAATACCATTCTCAAGGAGAGACCTTGCAGAATGGCGCCAGCTATCCACATTCCCTTGAGCGGTCACGCACGCTTCCCACATCAATGGAATCTCATGGTAGGAACTACCAAGAGGGCAATATGAATATTCCCCAAGTTGCTATGAACAGAAGTAAAGATTCGTCTCAAGTTGATGGATCGGGTTTCTCTGCACCAAATGCCTATCCTTACTATATGCATGGGGTCATGAACCAAGTTATGATGCAATCAGCAGCCATGATGCCTCAATATGGTCATCAAATTCCTCATTGCCAACCAAATCATCCGAATGGAATGACGGGATATCCTTACTACCACCACCCAATGAACACATCTTTGCAGCATAGTCAGATGTCTTTACAGAATGGTCAGATGTCTATGGTTCATCATTCTTGGTCACCGGCAGGAAATCCGCCTTCTAATGAGGTGAGGGTAAATAAACTTGACAGAAGAGAGGAAGCTCTGCTGAAATTCAGACGTAAAAGGAACCAACGTTGTTTTGATAAGAAGATTAGGTATGTGAATAGGAAACGCCTTGCTGAGAGGAGACCCCGCGTTAAGGGTCAGTTTGTTAGGAAGATGAACGGCGTGAATGTTGATTTAAATGGACAGCCTGACTCTGCTGACTATGATGACGAGGAAGAGGAGGAAGAAGAAGAAGAAGAGGAGAACCGGGATTCATCTCCTCAGGATGATGCTTTGGGAACTTGA

>AtPRR3
ATGTGTTTTAATAACATTGAAACTGGTGATGAAGTGGAAACCGAGAGGCAAGTGTTTGGTTCATCTGAAGAAGATGAATTTCGAGTTGAAGATACTGCTAGAAATACCAACAATGTACAGATTTCTCAACAACAGCAGCAACCGCTAGCTCATGTTGTGAAGTGGGAGAGGTATCTCCCAGTTAGATCGCTTAAGGTTCTTCTGGTGGAGAATGATGACTCAACACGCCATATTGTTACTGCCCTTTTAAAGAATTGCAGCTATGAAGTTACTGCTGTTCCGGATGTCCTTGAAGCCTGGAGAATTCTAGAAGATGAGAAAAGTTGCATTGATCTTGTCTTAACAGAGGTTGACATGCCTGTGCATTCAGGAACCGGTCTGCTGTCCAAGATTATGAGCCATAAGACACTTAAGAACATCCCCGTCATAATGATGTCATCACATGATTCTATGGTTCTGGTCTTTAAGTGTTTGTCGAATGGTGCTGTTGATTTTCTCGTGAAACCCATTAGAAAGAACGAACTAAAGAATCTTTGGCAACATGTCTGGAGAAGATGTCACAGCTCTAGCGGAAGCGGAAGTGAGAGTGGAATACATGACAAGAAGTCGGTGAAACCTGAAAGCACCCAAGGGTCAGAAAATGATGCCAGCATCAGTGATGAACACAGGAATGAAAGTGGGAGTAGTGGTGGTTTGAGTAACCAAGATGGTGGGAGTGATAACGGGAGTGGAACTCAGAGTTCTTGGACAAAAAGAGCCAGTGATACTAAGAGCACCTCGCCTTCAAATCAATTTCCCGATGCACCCAACAAGAAAGGAACCTATGAAAATGGATGTGCACATGTTAATAGACTGAAGGAGGCTGAAGATCAGAAGGAACAAATAGGCACGGGATCACAGACAGGAATGTCTATGAGTAAGAAAGCTGAAGAACCAGGAGATCTTGAAAAGAATGCAAAGTATTCTGTTCAAGCTTTGGAGAGAAACAATGATGACACGCTGAATCGCTCTTCTGGTAACTCACAAGTAGAAAGCAAAGCACCTTCATCTAACCGAGAAGATTTGCAATCACTCGAGCAAACTCTGAAAAAAACAAGAGAGGATAGAGATTACAAAGTCGGTGATCGAAGTGTGTTGAGGCATTCAAATCTCTCTGCATTCTCAAAATACAATAATGGTGCTACTTCTGCTAAGAAGGCTCCAGAAGAAAATGTGGAAAGTTGTTCTCCTCATGACAGTCCTATTGCAAAACTGTTGGGTTCGAGTTCAAGCAGTGACAATCCTTTAAAGCAGCAGTCTAGTGGAAGTGACCGATGGGCACAAAGAGAAGCTGCTTTGATGAAGTTTCGCCTTAAACGTAAAGAGCGATGTTTTGAGAAAAAGGTTAGGTACCATAGCAGGAAGAAACTAGCTGAGCAACGGCCTCACGTCAAAGGTCAATTCATTCGCAAGAGGGATGATCATAAATCAGGAAGTGAAGACAATTGA

>AtPRR5
ATGTGGCAAACGTGGCCACGTCAGCCAATTCTACTAGATATTTTTTCAAATCCAAATACTCTTTCCACAACCGTTAGATCATGGTCGGTTCGCCACCCACTTTCAATCATAACCGTTAAAACATTCGCTAGATTTTTTCTAGATATTTTCTTTTCTCCACACTATTATAGAAAGAATAAAGTTCTTTTTTTTGCTCTCTTCTCATTTATCTCTCCACTCACAAATATTTTGATTTGTTTTGTAACTGTTTCTCTTTCTCTGGAGCTTTCTTCTTCTTCTTCAATAATCGATTTAGGTTTTTCAAAGCTAAGTGTTTGTGTTGTGATAATGACTAGTAGCGAGGAAGTAGTTGAAGTGACGGTGGTTAAAGCACCTGAAGCTGGCGGAGGAAAGTTATCACGTCGGAAGATTCGGAAGAAAGACGCCGGTGTTGATGGTTTGGTGAAGTGGGAGAGATTTCTCCCGAAAATCGCGCTTAGAGTTTTGCTCGTTGAAGCTGATGATTCTACTAGACAGATTATCGCTGCTCTTCTCAGGAAATGTAGTTACAGAGTTGCTGCAGTACCTGATGGCTTAAAAGCTTGGGAGATGCTAAAAGGAAAGCCTGAAAGTGTTGATTTGATATTAACAGAGGTTGATCTACCTTCAATATCTGGATATGCTCTGCTAACACTTATCATGGAGCATGATATTTGCAAGAACATTCCTGTTATAATGATGTCGACACAGGACTCGGTGAATACTGTGTATAAGTGTATGTTGAAAGGTGCGGCTGATTATCTTGTTAAGCCGTTGAGGAGGAATGAGCTTAGAAATCTTTGGCAGCATGTCTGGAGAAGACAAACTTCACTTGCTCCTGATAGCTTTCCATGGAATGAGAGTGTTGGACAGCAGAAAGCCGAGGGTGCGTCTGCAAACAACTCGAACGGAAAGAGAGACGATCATGTTGTGAGTGGGAATGGTGGTGATGCCCAGAGCTCGTGTACAAGACCAGAGATGGAAGGTGAGAGCGCAGACGTGGAGGTTAGTGCGAGAGACGCAGTACAGATGGAGTGCGCAAAGTCTCAGTTTAATGAGACACGGCTTCTAGCAAATGAGTTGCAGAGTAAGCAAGCAGAAGCCATTGACTTCATGGGAGCATCGTTTAGAAGAACTGGACGACGTAACAGAGAAGAAAGTGTTGCTCAATACGAATCTCGGATAGAGCTTGATCTTTCTCTGAGAAGACCTAATGCTTCTGAGAACCAATCTTCTGGAGACCGGCCTTCTCTTCATCCTTCTAGTGCCTCAGCTTTCACACGGTACGTTCACAGGCCGTTGCAGACACAATGTTCAGCCTCCCCAGTGGTTACTGATCAAAGAAAGAATGTTGCAGCAAGTCAAGATGATAACATTGTGCTAATGAACCAATACAATACATCTGAACCGCCTCCAAATGCTCCAAGAAGAAACGACACCAGCTTTTACACTGGAGCTGACTCACCTGGTCCACCGTTTAGTAATCAGCTGAATTCTTGGCCGGGACAGAGTTCATACCCTACGCCAACCCCTATCAACAATATACAGTTCAGAGATCCCAACACAGCTTATACATCTGCAATGGCTCCTGCTTCACTCTCCCCAAGCCCTAGTTCCGTTAGCCCGCATGAGTACAGTTCCATGTTTCACCCATTCAACAGTAAACCCGAGGGGTTACAAGACCGGGATTGTTCCATGGATGTAGATGAGAGGAGATACGTCTCTTCTGCAACCGAACATAGTGCAATAGGCAATCACATTGATCAGCTTATTGAGAAGAAGAACGAAGATGGCTATTCATTATCCGTCGGGAAAATTCAGCAATCTCTTCAACGAGAAGCCGCTTTAACCAAATTCCGAATGAAGCGAAAGGACAGATGTTATGAGAAAAAGGTTCGTTACGAGAGCCGGAAGAAATTAGCAGAGCAACGACCACGAATCAAAGGCCAATTCGTTCGTCAAGTCCAATCCACACAAGCTCCATAG

>AtPRR7
ATGAATGCTAATGAGGAGGGGGAGGGTTCACGTTACCCAATCACTGATCGAAAGACCGGAGAGACGAAATTCGATAGGGTTGAGAGTCGGACAGAGAAGCATAGTGAAGAAGAGAAAACTAATGGAATTACTATGGATGTGAGAAATGGGAGTTCAGGTGGACTGCAAATTCCATTGTCGCAACAAACAGCGGCAACTGTCTGTTGGGAAAGGTTTCTTCATGTGAGAACCATTAGAGTTCTGCTTGTCGAAAATGACGACTGCACTCGTTATATCGTTACTGCACTTCTTCGCAATTGTAGCTATGAAGTTGTTGAGGCGTCAAATGGGATACAAGCTTGGAAGGTGTTAGAAGATCTAAACAATCATATTGATATTGTGCTAACAGAGGTGATCATGCCTTACTTATCTGGTATCGGTCTCTTGTGCAAGATTTTGAACCACAAATCTCGTCGGAACATCCCTGTCATCATGATGTCATCTCATGACTCAATGGGGCTGGTCTTTAAGTGCTTATCGAAAGGAGCTGTTGACTTTCTTGTTAAGCCAATAAGAAAAAATGAGCTTAAGATCCTTTGGCAGCATGTTTGGAGAAGATGCCAAAGTTCTAGTGGTAGTGGAAGTGAGAGCGGAACGCATCAAACTCAAAAGTCTGTGAAATCGAAAAGTATTAAAAAATCTGATCAAGATTCAGGAAGCAGTGATGAGAATGAAAATGGGAGCATTGGCCTGAATGCTAGTGATGGAAGTAGTGATGGGAGTGGCGCTCAGAGCTCTTGGACGAAAAAAGCTGTGGATGTTGATGACAGTCCACGAGCGGTATCTCTATGGGACCGAGTTGATAGCACTTGCGCCCAAGTGGTACATTCTAACCCTGAGTTTCCAAGTAATCAGTTGGTTGCACCACCTGCTGAGAAGGAGACTCAAGAACATGATGATAAATTTGAAGATGTCACAATGGGTAGAGACTTGGAGATTAGCATTCGTAGAAACTGTGATCTGGCCCTGGAGCCAAAAGATGAACCCCTATCTAAAACTACTGGCATTATGAGACAGGATAATTCGTTTGAAAAGAGCTCTAGTAAATGGAAAATGAAAGTTGGAAAAGGACCATTGGACCTCAGTAGCGAAAGTCCTTCAAGTAAACAAATGCATGAAGATGGAGGCTCGAGTTTCAAAGCTATGTCTAGCCACCTTCAAGATAACAGAGAACCTGAGGCGCCTAACACTCACTTGAAAACTTTAGATACAAATGAAGCTTCTGTTAAAATTTCTGAAGAGCTAATGCACGTGGAACATAGTTCAAAGAGGCATAGAGGAACTAAAGATGATGGGACACTAGTTAGGGATGATCGGAATGTGCTGAGGCGTTCAGAGGGCTCAGCTTTCTCAAGGTATAATCCAGCCTCAAATGCCAATAAGATTTCTGGTGGGAACTTAGGAAGCACTTCGCTTCAGGATAATAATAGTCAGGATCTTATAAAAAAGACTGAAGCAGCATATGATTGTCACTCGAACATGAATGAGAGTCTCCCCCATAATCATCGCTCACATGTCGGTAGCAATAACTTTGATATGAGTTCCACGACTGAGAACAACGCTTTCACAAAACCAGGAGCTCCAAAAGTAAGCTCAGCAGGATCTTCATCAGTGAAGCATTCATCGTTTCAGCCTTTACCCTGTGATCATCATAATAATCATGCCTCCTATAACCTTGTCCATGTCGCTGAGAGGAAGAAGCTACCGCCACAATGTGGATCCTCAAATGTGTACAACGAAACGATTGAAGGTAACAACAACACAGTGAATTACAGTGTGAATGGAAGTGTATCAGGTAGTGGTCATGGAAGTAATGGGCCATATGGAAGCAGTAACGGTATGAATGCTGGAGGAATGAATATGGGAAGTGATAATGGTGCTGGCAAAAATGGAAATGGCGATGGTAGTGGAAGCGGAAGTGGAAGTGGTAGCGGAAACTTGGCGGATGAAAATAAGATCTCTCAAAGGGAAGCTGCTTTGACAAAGTTCCGTCAGAAGAGAAAAGAGAGGTGCTTCCGAAAGAAGGTACGATACCAAAGCCGGAAAAAACTAGCAGAACAACGCCCTCGAGTGCGAGGCCAATTTGTGCGTAAAACAGCCGCTGCAACTGATGATAACGACATAAAAAACATTGAGGATAGCTAA

>AtPRR9
ATGGGGGAGATTGTGGTTTTAAGTAGTGATGATGGTATGGAGACTATAAAGAACAGAGTAAAGTCATCGGAAGTTGTTCAGTGGGAGAAGTATTTGCCTAAAACTGTACTTAGGGTTTTGTTAGTTGAATCTGATTACTCAACTCGTCAAATCATCACTGCCCTTCTTCGTAAATGCTGTTACAAAGTTGTAGCTGTTTCTGATGGTTTAGCTGCGTGGGAGGTTCTAAAGGAGAAGTCACATAACATTGATCTTATACTAACAGAGCTGGATTTGCCATCTATATCTGGTTTTGCTCTGCTTGCTTTGGTAATGGAGCATGAAGCTTGCAAGAACATTCCTGTCATAATGATGTCTTCTCAAGATTCGATAAAAATGGTGTTGAAGTGTATGCTGAGAGGTGCTGCTGATTATCTAATCAAACCAATGAGGAAAAACGAGTTGAAAAATCTATGGCAACATGTTTGGAGAAGACTGACTTTGCGTGATGATCCTACTGCTCATGCTCAAAGCTTACCAGCTTCACAGCACAACCTTGAAGATACTGATGAAACTTGTGAAGATTCCAGATATCATTCAGATCAAGGAAGTGGTGCTCAGGCTATCAATTACAATGGTCACAATAAGCTGATGGAGAATGGCAAATCAGTGGATGAAAGAGACGAGTTTAAGGAAACTTTTGATGTGACAATGGATTTGATTGGTGGAATTGACAAGCGTCCTGATAGTATTTATAAAGACAAGAGTCGAGATGAGTGTGTTGGTCCTGAGCTTGGACTTTCTCTGAAAAGATCTTGCTCTGTAAGTTTTGAGAACCAAGATGAAAGCAAGCATCAAAAGCTTAGCCTCTCTGATGCGTCGGCCTTCTCAAGATTTGAGGAAAGCAAGTCAGCAGAAAAAGCCGTCGTTGCTTTAGAGGAGAGTACTTCAGGTGAGCCAAAGACACCAACCGAATCACATGAAAAGTTAAGAAAAGTAACATCTGATCAAGGAAGCGCCACAACGAGCAGCAACCAGGAGAATATCGGATCATCAAGCGTAAGCTTCCGTAACCAAGTTCTTCAGTCCACAGTAACGAATCAGAAGCAAGATTCACCCATACCGGTAGAATCAAATCGCGAGAAAGCAGCTAGCAAGGAAGTAGAAGCTGGTTCTCAAAGCACCAATGAGGGGATTGCTGGACAAAGCAGTAGCACAGAGAAACCAAAGGAAGAAGAAAGTGCGAAACAACGTTGGAGTAGAAGCCAGAGAGAAGCTGCATTGATGAAGTTCCGGTTGAAGAGGAAAGATCGATGCTTTGACAAAAAGGTTCGGTACCAGAGCAGGAAGAAGCTAGCAGAACAACGTCCTCGAGTGAAAGGCCAGTTCGTGCGAACCGTGAATTCAGACGCGTCTACAAAATCATGA

>AtPRR9b
ATGTATTTAACTTGCAGATTTGAGGAAAGCAAGTCAGCAGAAAAAGCCGTCGTTGCTTTAGAGGAGAGTACTTCAGGTGAGCCAAAGACACCAACCGAATCACATGAAAAGTTGAGAAAAGTAAGATCTGATCAAGGAAGCTCCACAACGAGCAGCAACCAGGAGAATATCGGATCATCAAGCGTAAGCTTCCGTAACCAAGTTCTTCAGTCCACAGTAACGAATCAGAAGCAAGATTCACCCATACCGGTAGAATCAAATCGCGAGAAAGCAGCTAGCAAGGAAGTAGAAGCTGGTTCTCAAAGCACCAATGAGGGGATTGCTGGACAAAGCAGTAGCACAGAGAAACCAAAGGAAGAAGAAAGTGCGAAACAACGTTGGAGTAGAAGCCAGAGAGAAGCTGCATTGATGAAGTTCCGGTTGAAGAGGAAAGATCGATGCTTTGACAAAAAGGTTCGGTACCAGAGCAGGAAGAAGCTAGCAGAACAACGTCCTCGAGTGAAAGGCCAGTTCGTGCGAACCGTGAATTCAGACGCGTCTACAAAATCATGA

Carica papaya
>CpPRR3
ATGTGTTATGACAAAAAGGAAGTTGCCAATGGAGTAGTTAGTGAGAGGCAAGGATTAGGGTCATCTGAGGAAGATGAGTCAAGGGTTGATGATGTAAATGCAAACAATGTGTCGCGAGGACAGCTCCAGGTGCATGATGTGTTGCAGATACCTCAACAACAACCTCAAGGTTCCATGATACGTTGGGAAAGGTTTCTCCCAGTTAGGTCTCTCAAGGTTCTTTTGGTAGAAAATGATGATTCGACACGCCAGGTTGTTAGTGCTTTGCTACGAAACTGCAGTTATGAAGTTGTAGCTGTTGCAAATGGTCTTCAAGCTTGGAAAATTCTAGAAGATTTTAACAATCATATCGATCTTGTCCTAACAGAGGTGGTCATGCCTGTATTATCAGGTGTTGGTCTCTTATGCGAGATTATGAACCATATAACTTTCAAGAATATTCCTGTTATAATGATGTCATCTCATGACTCTATGGGTCTGGTCTTTAAGTGTTTGTCAAAGGGTGCAGTTGATTTTCTTGTGAAACCTATTCGGAAAAATGAATTGAAAAACCTCTGGCAGCATGTTTGGAGGAGATGCCACAGTTCTAGCGGTAGTGGGAGTGAAAGTGGGACACACACGAAAAAATCTGTAAAGTCAAAAAGCATTGACGAATCTGAAAATAATACAAGCAGCAGTGATGAACATGATAATGGAAGCAATGGTCCTAGTACTGGGGATGGAAGTGACAATGGAAGCGGTACACAGAGTTCATGGACAAAAAAGGCTGCTGAGATTGACAGCCCACAGCCAATGTCACCCTCCTACCAGTTGACCGATGCTCCTGATAGCACTTGTGCTCAGGTGATCCGCACAGAGCCAGGAACTTTTAGCAACAGGTGGATGCATGTTGCTGACACAAAAGACTACCAAGAGCAAGATGAACAGCTTGATAATGCTGAAATAGGCAAGGATGGAGTACGAGTATTAAAAATTCCAGATTTCGAATGTGAAGATCAGTGTGAGAATTTATCTACCCCCAAATCAAAGAAAAGGCAAACTAAGTTTCCTGAAGGAGATAATAAACTTCTTGATGGTGAAAAGCTGGAGCATAGCAATGATAATTCGACTGGGAAACCAAGAGATAGAGCTCTTAGTGTACTTAATCAAATCACGAAGTCTGCCATTCCGGAGTATGAAAGCAAAGACATTGACACTCCAAATGCCGCCTCTGGTATCTTGCAGATTAAAGACAAAGCATGCAGTGATCCTGGAGAAATGCCATCCCTAGAATTAACTTTGACAAGACCAAGAGGCACAGTAGATGACCGAAATGCTGCAAATGACGATCGCAATGTGTTAAGACACTCGGATTTGTCAGCATTCTCAAAATACAACACTGCATCATCTGCTAACCAGGGTCCTACTGGAAACATAGGAAGCTGTTCTCCTCTAGATAACGGTTCTGTTGCAATGAAGACGGACATGATGCACAATTTTCCATCACATTCAAATAGTACTCCGTTAAATCAGCAATCTAATGGCAGTAGCAACAACGACATGGCCTCTACTGCTAAATGTGTTACCCCGAAATCAGAAGCTTTCAGTGATAAATCTGAGTCCATATCAGCATTTAAACCTTTTCACTCTTCTGCTTTCCAACCTGTGCAGACTGGTTGCATTTCCTCATCTCAACAGTTTTTAACTGAAAATGCAAGTGATGTAGGAGTAAACACAGTTCAAGCACAAGTGAGAGGCTCGAACAAACAGGTTCAACTCCAAGTCCATCATCATCACCACCACTATCACCATCATGTTCACAACATGCAGCAGTGTCAGCCACAGGCAGACCATGACGATATCTTGCTGACAAAAATGGCAGCTGATGCTCGTCAATGTGGATCATCAAATGCCTTTGAAGGATCCATGGAAGAGAATGTTGTCAACTACAGTGTAAATGGAAGTGTCTCAGGGAGCAACCATGGAAGCAATGGACAAAATAGGAGTGGTACTGTGTTGAACACTGAACAGCAAAACATGGAAAGTGACAATATGGCTGGTGGAAATTGTGGGGGTTGGTGTGATAGTAGGAAAAGTAGTGGGAGTGGAGCAGATGAAGATCGGGTTACACAAAGGGAGGCTGCTTTGATCAAGTTTCGCCAGAAAAGGAAAGAACGATGTTTTGAAAAAAGGGTCAGGTACCATAGTAGGAAAAAACTGGCGGAACAACGGCCACGAGTCAGAGGACAATTTGTTCGGCGGTATGATTCCAAATCTGGATTTGATTGTCCAAGCAGCGGCGTAGCATCTGAAGACAATTCTTGTGACAGTGTAAGATAG

>CpPRR5
ATGGGTGAGGTGGTGGTGAGCAGTGAGGAGGTGGAGGTGAAGTTTGGCAGCAAGAGAGAGGAGAAGGCGGTGGAGAACGGCGGCGGAGAACGGAAGAGCAAGAAGGACGGATTGGTGAAGTGGGAGAAATTCTTGCCCAGGATGGTACTGAGGGTATTGCTCGTTGAAGCTGATGATTCCACCAGACAAATAATTGCTGCTCTGCTTAGAAAATGCAGTTACAGAGTTGCTGCTGTGCCTGATGGCTTGAAGGCATGGGAGATGCTGAAGGGAAGACCTCATAATGTAGATCTTATACTGACAGAAGTTGATTTGCCATCAATATCCGGATACGCCCTTCTTTCGCTAATTATGGAGCACGAGATTTGCAAAAGCATTCCTGTTATAATGATGTCCTCACAGGATTCAGTTAGTACAGTATATAAATGCATGTTGAGAGGTGCCGCTGATTATCTTGTTAAGCCTATAAGGAGAAATGAGCTGAGAAATTTATGGCAGCATGTTTGGAGAAGACAAGCTTCAATAGTTGGTGGAAGTGGCCCCCAAGATGAGAGCGTTGGACAGGAAAAGCTAGAAGCTACTTCTGAAAATAATGCTGCAAGTAATCAGTCAAGTGGGTTCTTGGCTTGTATTCAGAGAAACAAGGAGCAAATTGAGAAAGGGAGTGATGCTCAGAGTTCTTGTACGAAACCAGATGCGGAAGCTGAGGGTGAGAACACAGACAATATGCAGGAATTTTCACGGCTGAATTGGAGTAAATCTTTACCAAACGATACAGAAATGCAGCAGCAGAAAGATTATCCTGATTTCTGCACAACACTGCCTGTAAATGAGAATGAGGCTGGAGGGTCGTTGGCTGGTGCCTGCGAGGAAGTGATTAAAGCATCTGCAAATGAAGATGTTGGGCCAGAAAGTCAAGGGAGGGATGCTAACAGCAAACATGAGGCTTGTAATGGCTTCGCCAGCTCCTCAAAAGAAGCCATTGACTTCATGGGGCAATTTAGTACTCACAAATCTTCTTCAAATAATGGCATGAACAAGTATGCTAGCAGAGTCAAACAGCCCGAGTCTTCTACTTCGCCAAGTGTTTCTAATCAAAGGAAAGAATCGGGAAATATTTATGAGAAGAATTTATCTGATCTTGTTGGTGGGTTTAATTGCAATGCTCCTGGTCCACCAAGTACCCGAACCTCTCTGACTATTTGGCAATCTGATTGTCAATCTAAACAATCTGAACATGCCACAACTTCATGGCCTCAACAGAGAGTTTTTCCTGTCCCCATTCCTGTAAAAAGCATCAAAGTTAATAGTCCAAACACAGGCTATGGTTCTGGGATTCCTCCAATATTTGGAAGACAATCAACTCCGTCACCAACACTGAGCCCTAGTTCAGCCAGCCAGCCAGAACTCACCTCTCGAGCAAATACATTTTGTTGGCCTGGTTTTGGAAACAGTAAATGTGAACAGATGTATTTGTATGATGGAATGGGTCAAAATGTAAATTCAGCCAACCAATTTATGTACAACAAATTGGAATCTTTGGAGGATCGAGGACATATTTCTCCCGCCACGGTTCAGAGTGGAACTAGCAGTTTCTGCAACGGTGCCATAAGCCATCTTAACGGCTATGCCAGTGCATGTGGAAGTAATGTCAATGTTGATCAGGTTTCCCTTATCAGAGAAGGTCCAGAGAGCAAGAACGAAGACAGTTATTTCTCTCCCACGAGAAACTCACAACGCTCCCTCCAAAGGGAAGCAGCTCTTACCAAGTTTCGTCTAAAGCGAAAAGATAGATGCTTTGAGAAAAAGGTTCGGTACGAAAGTAGGAAAAAGCTTGCTGAGCAGCGACCTAGAGTAAAAGGACAATTTGTTCGACAAGTGCACCCGGAACATGCACCTTCAACAGCAGAAAATCACTATGGCAATTCATCTAGTGGCTAG

>CpPRR7
ATGGGAATCACTGAGACCAGTAACAATGGTCCTGTGACCAATGGAATACTAAAATCAAATCATCAAGTACAAGATGATCCAAATGGAACAAAGGATGAAGTAATAAGAGACAGACCGTTCTCAAAGGAAAATGAATCGCGGATTAATGAGGATGTAAAAGATGTAGATGATGGAAAGACTGGAGCAGTTCAGCTCCAGGAGCATGTACAGATTCTCCATAGGACACAGAAGAGATCCCAAGGGCCTCCAGACCACTGGGAGAGTTTTCTTCCTTTTAGGTCTGTGAAAGTTCTACTGGTGGAAAATGATGATTCTACCCGCCACATTGTTTATGCATTGCTGCGCAATTCTGGGTATGAAGTTATTGCTGTCGAAGATGGCCTGCAAGCCTGGAAAATTTTGGAAGATATGACAAATCGTATTGATCTTGTTCTAACAGAGGTAGTCATACCATTTTTATCAGGCATTGGCCTTTTGTGCAAGATAATGGGTCAAAAGACTTGCAAGAATATTCCAGTGATCATGATGTCATCTCACGATTCTATGAGTGTAGTCTTTAATTGTTTGTCCAAGGGTGCAGTTGACTATTTAGTGAAACCTATACGAAAGAATGAGCTTAAAAACCTTTGGCAGCATGTTTGGAGGAAATGCCACAGTTCTAGTGGTAATGGGAGTCAAAGTGGCATTCAGACCGAAAATTCCTCAAAATCCAAGAGTGTTGAGGACTCGGACTACAATACTGGTAGCAACACTGAAGATGATATAAGAAGCATCGGTTTTAATGCTTGGGATGGAAGTGACAATGGAAGCAGCACTCAGAGCTCATGGACAAAAAGGACAATCGAAGTTGACAGTCCCAAACCAATGTCACCAGGGAACCAATTAGCTGATCCACATGATAGCACATGTGCCCAGATCATTCGGTCAAGATCTGAAGCAATTCATAACCATTGGTCTGTGACTGCTGCAAAAGAGTACAAAGGGGACAAATTCAGCAATATGCTGATGGGCAAAGATTTGGAGATAGGAGTCACTAGAATTCCAAAGTTGCAACTTAAAAAGCCAAGTGCAGAGGCACTAAGCCACATAGATGGTCTACCTGGATTGAACTCGATTAAAGATCCTGAGAATTTATTGAAAGAAGAACAGGGAAACAACAGTGATAGTGATTTAAAAAGCCTATCTGCTGATCTTATTGGTTCCATCAATTACAAGTGTCCTAATCTAGAATGTGTTACCTTTGGCACCACAAATCTTCTTCCCAAGGTCTCTGATGATAAAAATAAGGTTATTTATGACACGGAGGAAATGCCATGCCTTGAGCTCAGTTTGAAAAGATTTAGTGATGCTGGAGATACTAGGAAAAGTACCCATGACAGAAATCTTCTGAGACATTCTAAACTCTCAGCCTTCTCAAGGTATAATTCTGCTTCAACTGCTAATCAGGCACCAACAGGCGATATAGGTAGCTGTTCTCCACCAGATAATAGCCTAGAGGTTGCAAAAACAGAATCAGAGCAGAATATTCAATCTAACTCAAATCAGCATTCCAATAGTGATAGCAACAACAATGATATGGGTTCTACAACTAATAATATTTTCAACCAATCGATTGTATTTAGTGACAAACTAGCTCCTATATCCAAAGACAATTGTCTCCCTCCTTCCGCTCTGCACCCTATGCAAAATGGTCATTTCTCTCCTGTTGAGCCTGTGTTAGAAGGTAAATCTGATGCTGCAGTGCCTAAGGCAATGTTGGCTCAAGGGAAGAGCGTGGACCAGCTGATCCAAGTGCACCACTACCATCACCATCATCACCAGTATTACATCCATAATGTGCCACAGCAGCAGTATCTAGCCAACCATGATGATACGCCTTTAAATAACATGGTGGAAGCAGCTTCCCAATGTGAGGCATGCAATTCATCAGGTGCATATAATGATGGTAGTGATTGTGCTCATGCTTTCAGTGGAAGCGCCTCAGGCAGTAACCATGGGAGCGATGGGCAAAATGGAAGCAGCACTGCATTCAATGTCACAGGAACAAACATGGACATTCACAATAGGATATCTGGTGAAGGTGGAGCTGGTGGTGAAATCACCATTGGGATAGATCAGAATCATTTTTCACAGAGAGAAGCTGCCTTGAACAAGTTCCGCCAGAAAAGGAAAGAAAGATGCTTTGAGAAGAAGGTCCGATATCACAGTAGGAAGAAACTAGCAGATCAAAGACCTCGTGTTCGTGGACAATTTGTGCGACAGGTGGTGCACGAAAACAGAAACGAGGACAAGCATTGTCAACCTGACGCTAGTGAGAGATAG

>CpPRR9l
ATGGGTGAAGTGGTGGTTAGCAGCGACGAAGTCATAGAGGTGGTGTTTGAGAAGGAACCGGACAAGGACAAAAACTGCAAGAAGAATGAGGATAAAGATGGATCATCGGAGGTGGTCAGGTGGGAAAAGTTTCTGCCTAGGATGATGCTTAGGGTTCTGTTGGTGGAAGCCGATGATTCAACTCGCCAGATCATAGCTGCTCTTCTTCGTAAATGTAGTTACAGAGTTGCTGCTGTTCCTGATGGTTTGATGGCATGGGAAACTTTGAAAGGACGACCGCAAAACATAGACATCATATTGACTGAAGTGGAGTTACCATCAATCTCTGGGTTTGCACTCCTTACTTTAGTAATGGAGCATGACGCGTGCAAAAATATTCCTGTCATAATGATGTCGTCACATGATTCAGTTAGCATGGTCTTAAAGTGCATGTTGAAAGGTGCAGCTGATTTCCTTATTAAGCCTATTAGGAGGAATGAACTGAGGAATATCTGGATGCATGTTTGGAGAAGGAACACCCTAAGTGGTGGCTCTTTTCATCAAAGCTCACCCATTAGGCAACCTAAAGTTGAATGTACTTCTGAAAACACTGCAGCAAGCAATTATTCAAGTGACCATGCAACTTCTGCACAGAAAAACAAGGAATGCAGTGAGAAAGGGAGTGATGCCCTGAGCTCTTGTACAACGCCTTACTTGGAAGCTGAGAATGCATGCATGCAAAATATGCAGGGACTTTCACAGCTAAAATACAGGACTACTTCTATTTTGAGAAATAATGATCAAGGGAAGCAAGTAGAGCATCTCAAACCATATAAACAATCTATTATACCAGAGTGCAAAACTAGAGAAAATTTTGACAGATTAGGAACGAAGGTTGCACCTTGTGTACAAGCTTATAGATCAAGTGCCTTGAAACTTGAGGAAGGTCGTGACTGTTGCAAAGGAACGGGTCAAGAGGAAGGTGTATGCACAGAAAATGATGATAGAGATGCTGATATTTCTGTGGAGACTCATGGCTGCAATGATGAACCGGTTGAGCCTTCTAATGGTGCTGTTGACTTGATTGGTACATTTGATGACCATCAATTGCAGCGCATGTATAGATGCTCAAATTTTAATGACTACATAAACAAGTCGGAATTTGCTCCACAACTTGAACTTTCTTTGACAAGATTCTGTCCCAGTAGCTCAATGAACAAAGGGTACCATGAAAGGCATACTCTTAACCATTCTAATGCCTCGGCCTTTTCATGGTATAACAATAACAAGTTGTTGCAGTCCCCTTTCTCAGCAGTGGTCGGTAGTTGTACAGAATCGAAGGAGAAGGCAAGTAAAACACATGAACCGTCATCTTACAAACTTTCTCAAGACAGCGTTCGTTCTTCTGACCCGCTAGGTGCCCCATCAGGGGGAAGTCAGGGGAATATAATTGGTCAATCTCGAGACATTGGGCTAGTTTCTGTTTCTGGAGTGACATTTGATAGTACATGTGCAGAACAGATTCATGTTTCCACACCGATATTCTATAGCCAATCTGGTTTACCTCCACAAGGGTGGAATGCAAAACCAGCCTTCCTGAAAGAGCAATCTCCTTTTCCTATGAGCACTTCTGTTCATTCCGATTCTTACACCCATGACGCAGAACAAGGTTACCATCTATGTGATGAAACCATCGGTTATTCTGTTGACCAAGCAGTGCCCGTGCACAGCACTTTGGAAACCGTCAGGGAACTGAAACAGGGTTCTCCGGCTGCTGGGCAGAGCACTGGCACTAGCTTATATAATAGTGTTACGAGTCACGACAACAGTGATGCTTGTGGAGGCATTTGTAGAAGTGACGGGAATGGTACTTCAGCATCAGCAGTCGAGAAGTCCAATGCTTCCGAAATATTGAACGAGGGAAGTCTTTTAGTTCATAATGGCTTGAAAGTGATGGAGTCTCACCGCTCCAGCCAAAGAGAAGCGGCTCTAACAAAATTCCGACTGAAGCGGAAAGAAAGATGCTATGAGAAAAAGGTTCGATATCAAAGCAGGAAAAGACTAGCAGAGCAGCGTCCCCGAGTAAAGGGCCAATTTGTTCGTCAGGCTATGATCATGTATGGGAATCTCGATAGAGTGCGTATGCATTACGATATTTCAACTTCTACCATCTCTCCTCTTATTAACTATGGAAATCAAGAAACGAGTCTTTTTAAGTTTAAAAAAAAGTGCGAGGCTGTCCATATCAACATCCATAAAGCAACCGCCGAGGGTGACTTCGGTCGTGGTGGAAGAAATAGTAGAGGCCGTGATTGGATTTTCCGGTTCGGCGATGAGGTGACTATTGGGGTTTATGGATTTGGATTGAGCCGAAAAGATTGGAAAGGTGATGGTTTGAGGAAGCATAAAGGCTATGTAAGGCAAGAAAATATGAGATCTAATTAG


Populus trichocarpa
>PtPRR1/TOC1
ATGAAGGAGAGTGGTAATGGTAAAAGTGTAGGAGGAGGAGGAGCAGGAGATGGTTTTGTTGATAGAAGCAAAGTGAGGATTTTGCTATGTGATAATGATGCTAAGAGCTCTCAGGAGGTTTTCACTCTCCTTTTGAAGTGCTCTTATCAAGTTACATCAGTGAGATCGGCTAGGCAGGTGATTGATGCACTAAATGCTGAGGGGCCTGAAATCGACATTATACTTTCTGAAGTTGACATTCCAATGACCAAGGGCATGAAAATGTTGAAGTATATCATGCGGGATAAAGACTTGCGGCGTATTCCTGTGATCATGATGTCGGCACAGGATGAGGTCTCAATTGTTGTAAAGTGCTTGAGACTTGGAGCGGCAGACTATCTTGTAAAGCCTTTACGTACTAATGAGCTCTTGAACCTGTGGACACACATGTGGAGAAGGCGGCACATGCTTGGATTGGCGGAGAAGAACATCTTGAACTATGACTTTGATCCGGTTGCATCAGATCCTAGTGATGCTAATACAAACAGTACTACGCTGTTTTCAGATGACACGGATGACCTGTCTCGTAGGAGCACCAATCCTGAAATGGGGATGTCAACTCATCAGGAAGATGAGCCTTCTCCTGGTGATCCACAGAAATATCGACCTGATGTGCCAGGGATCAGTGACCGCCGAACAGGACATTTATCTTCTGGTCCGAAGAAGAGTGAATTGAAGATTGGCGAGTCCTCTGCCTTTTTCACTTATGTCAAACCAAGCACGGTAAAAAACAATTCCCAAGGAGTTGCTCTTATTGAGGACAATACTAATCAAAATTTGAGGATGGAAGAGAAACTTCAAGTATGTGGCGAGCAAATGCTTAATGATGCCCATTTACAAGAAAATGGGGAGGCATTGGAAATCCACTCACAAGTAGATGACTTCCGTAGCAGTTCTAGCATTCCTGACTCTCTCTCTCTGGAAAGATCTTGCACCCCTCCTATGTCAAGAGAATTTCCCCAAAGAAATTTCAAGGATGATCGGGTGCTTATGCATCAAACGAATGAACCTCAATTAGATGCGTCAAGTTTATCAACACAAAGTGTGTATCCATATTTTATGTCAGGTGTTGTAAATCAAGTTATGATGTCATCATCAGCACAACTCTATCAAAAGAACCTGCATGAGTTGCAAAGCCTTGGAACTTCAGCCATGCTTCCTGGATACAATCATCTTCCCCAATGTCCACCTCACATGTCTGGGATGGCATCATTTCCTTATTACCCTGTTAGTATATGCTTACAACCTGGTCAAATGCCTACCACTCCGTCATGGCCATCATTTGGATCTTCGACGTCAGCTGATGTGAAACTAAATAAAGTAGATAGAAGAGAGGCAGCATTAAATAAGTTTAGACAGAAAAGAAAAGAGCGCTGTTTTGATAAGAAGATAAGGTATGTTAATCGGAAAAAACTTGCTGAAAGGAGGCCTCGAGTGCGGGGACAGTTTGTGAGGAAGGTAAATGGTGTAAATGTGGATCTCAATGGGCAACCTGCTTCTACGGATTATGATGAAGATGAAGAAGAGGACGGTGATGAGCAAGCATCAAGGGATTCTTCTCCTGAGGATGATGCTTCAGGATCTTAA

>PtPRR5a
ATGGGGGAGGTAGTGATTAGTAGTGGTGAAGAATTGGAGGTGAGATCAAAGAGTGAAAGAGAGGAGGAAAAACAGAGGAAACAGAGTAAAGAAGAAACAGGGGAGGTGAAAAAAAAGAAGAAAAAGAAGAAAGAAGGTGAAGGTTTGAATGACGGGTTAGTGAGATGGGACGGGTTTCTACCAAGAATGGTGTTAAGGGTGTTGTTAGTTGAAGCTGATGATTCAACTAGACAGATAATTGCTGCTCTTCTTAGGAAATGTAGTTATAGAGTTGTTAGTGTTCCTGATGGCTTAAAGGCATGGGAGATACTGAAAGGAAGGCCACATGGCATAGACCTCATATTGACTGAAGTGGATTTGCCTTCGATATCTGGATATCCTCTTCTTACTATTATAATGGAGCATGAGATTTGCAAAAACATTCCAGTCATAATGATGTCCTCTCAGGATTCAATTAGTACAGTTTATAAATGCATGTTGAGAGGTGCTGCTGACTATCTTGTCAAGCCTTTAAGGAAAAATGAACTGAGAAACTTGTGGCAACATGTATGGAGAAGACAATCTATGAGCCCTAACCAGTTCCCATTTTCTCAGTCCCTTGCTGGAGGAAATGGCCCCCAAGATGAAAGCGTTGGACAGGACAAGATTGAAGCCACTTCAGAAAATAGTCCTGCCAGCAATCATGCAAGTGGAGAAATGGCTTCTATTCAGAGGAGTAAAGGGCAAACAGAGAAAGGGAGTGATGCTCAGAGCTCTTGTACAAAGCCAGACTTGGAAGCTGAGAGCAGCCACATGGAAAATATGCAGGAATTTTTGCAGCCTGTACGGAGCATATTTTCGTTGACTGACATGAATATGCAGAAGCGTGAAATGCATGTGAATCTGGGTCAGAAATTGCTTTTGCATGATAGAGAAGCTGAAGGTTCAGCTGCAGCTGCCCGTGAGGATGCTAATATAATGGATGTGGACAAGGAAATTTCTCCAGGAAATGGAAGAACGGGTGCTTATGTTGCCATTGAGAGTTGTGACAACGATGTTGCCCTTGCCAACTCTCACAGAGAAGCCTTTGATTTCATGGGAGCATCTACTAATCGCAGTTCTTCTTTCAATAACGTCAAAATCAACTTTGATTCTTCTCCACATTTGGATCTTTCCTTGAGAAGATCTCATCCAAGTGGGTTTGAGATTCGAGATACGGAAGAAAGACGGGCTCTCTGGCATTCTAATGCCTCAGCCTTTACACAGTACATTAACAGGCCTCTACAGCTTCCACATTCAGCACTGGAAAGTACTGGGAATCAGAAGGAATTAGGAACCAATTATGACAGAAAAATATCCAGTACTGGCTATAACTCTGATGCCCTGAGTCTAGCACCAAGCACTCAAAAAAGTGAGATCTCTCTGGCAGCTGGTCAAACCAAGGAATCTGAAATTGCAACTTCATCTCCTGGACAAAGAGTGTTTCCTATCCAAATTCCAGCGAAAGAAACAAGACTCAATAATCTATGCAACAGTTATGGCTCCGTGTTTCCCCCAATATTTTGCAAACAATCAGGTTTATCACCAATGATGAGTCCAAGTTCAGCTTGCCAGCAAGAGCCCACCTATAAGGTGAACCAATTTCAACATTCCAATCATGGGAGCACCTCCGAACAGAATCGACTTGGTCAACATACAAATGATTCCACCAATGGTTCACTACAAAAGCAAGAAGACAGATTGGATTCCTTGGAAGATAGAGGACTTATTTCTCCTGCCACTGATCAGAGTGCAAGTAGTAGTTTCTGTAATGGTGCTGCAAGTCACTTTAACAGCATGGGCTATGGAAGCACTTCTGGAAGCAATGGCAATGTTGATCAAGTTGCTATTGTCAGGGATGCTTCTGAGAGCAAGAATGAGGAAGGTGCTTTCACACATTCATACTCTCACAGATCTATTCAAAGAGAAGCAGCTCTAACTAAGTTTCGCTTGAAGCGAAAAGAGAGATGCTATGAGAAGAAGGTTCGGTATGAGAGCAGAAAAAAACTTGCTGAGCAGCGTCCTAGAGTGAAAGGACAATTTGTTCGCCAAGTGCACATTGATCCCTCACCTGCGGAAACTGATCAGTAG

>PtPRR5b
ATGGGAGTGGTAGTGGTTAGTAGTGGTGAAGAATTGGAAGTGAAAACAGGAAGTGAAACAGAGGAGGAGAAACAGAGTAAAGAAGAAACAGAGAGTGAGACAGGAGAGGTGAAGAGGAAGAGGAAGAAGAAAGAAGGTGAAGGTTCAGATAATGGGTTAGTGAGATGGGAGAGATTTTTACCAAGAATGGTATTAAGGGTTTTGTTAGTTGAAGCTGATGACTCAACTAGACAGATAATTGCTGCTCTTCTTAGAAAATGTAGTTATAAAGTTGCTACTGTTTCTGATGGCTTAAAGGCATGGGAGATACTGAAAGAAAGACCACACAACATAGACCTCATATTGACCGAAGTGGATTTGCCTTCAGTATCTGGATATGCTCTTCTTACTCTTATTATGGAGCACGAGATTTGCAAAAACATTCCAGTCATAATGATGTCCTCTCAGGATTCAATTAAAACAGTTTATAAATGCATGTTGAGAGGTGCTGCTGACTATCTTGTTAAGCCTATTAGGAAGAATGAACTGAGAAACTTGTGGCAGCATGTATGGAGAAAACAATCTTCCCTTGGTGGAGGAAATGGCCCCCATGATGAAAGTGTTGGACAGGATAAGACTGAAGCAACTTCTGAAAATAATGCTGACGGCAATCATTCAAGTGGTGAAATGGCTTCTATCCAGAGAAGTAAAGAGCAAGCGGTGAAAAGGAGTGATTCTCAGAGCTCTTGTACGAAGCCAGGCTTGGAAGCTGAGGGTGCCCACATGGAAAACATGCAGGAATTTTTGCAGCCAGTATGGAGCAAATTTTCATTGACTGACACGAATATGCAGAAGCACGAAGAGCATGTGAATTTGGGTCAGAAATTGCTTGTGCGTGATAGTGAAGCTGAAGGTTCAGCTACAGCTGTCTGTGAGGATTCTAATAAAATTACGGTGGACAAGGAAATTACTCCAGGAAGTGGAAGAGTGACAGCTAATATTGCCATCGAGGGCTGTGACAAAATTGGTGCCCTTGCCAACTCTCCTAGAGAAGCCATTGACTTCATGGGAGCATCTACGAATCACAGTTCTTTCAATAACGTCGAAATCCACTTTTGTTCTTCTCCACATTTGGATCTTTCCTTGAGAAGATCTCATCCTAGTGGGTTTGAGACTCAAGTTACAGAAGAAAGACACACTCTCCGGCATTCTAATGCATCAGCCTTTACGTGGTACACTAACAGGGCTTCACAGCTTCCACATTCAGCACTGGCAAATACTGGGAATCAGGAAGAATTCAGGGCCAATTATGACGGAAAAATATCCAGTAATGTTAATGGCTACAACTCTGATGCCTTGAGCCTAGCACCAAGTACTCGAAGAAGTGCTATCTCTCTTGCTGCTGGTCAAACCAAGGAATATGAAATTGTAACTTCATCTTCCGGTGAAAAAGTATTTCCGATCCACATTCCTGTGAAAGATACAAGGTTCAATAATCTATGCAACAGTTATGGTGCTGTGCTTCCCCCAATGATGAGTCAAAGCTCAGCTAGCCAGAAAGAACCCATCCATAAAGTGAACCCATTTCAATGTTCCAATTATGGGAGCACCTCTGTACAGCTCTGTGATCGACTTGGTCAAAATGCTAATGATTCCATCAATGGTTCTCTACAAAAGCAAGAAAACAAATTGGATTCTTTGGAAGGTAGAGAACATATTTCTTCCGCCACTGATCAGAGTGCAAGTAGCAGTTTCTGTAATGGAGCCGCAAGTCACTTTAACAGCATAGGCTATGGAAGTGCTTCTGGAAGCTACAGCAATGCTGATCAAATTGCTACTGTCAGTGCTGCTTCTGAGAGCAAGAATGAAGAAGGTGTTTTCACACATAATTCAAACTCTCATCGATCTATTCAAAGAGAAGCAGCTCTAACCAAGTTTCGCTTGAAGCGTAAAGAGAGATGCTATGAAAAGAAGGTTCGGTATGAGAGCAGAAAAAAACTTGCTGAGCAGCGTCCTAGGGTAAAAGGACAATTTGTACGTCAAGTGCACATTGATCCTTCACCTGCTGAAACTGACCAGTAG

>PtPRR5c
ATGGAGGTGGAATTGAAAGAGATGCTTAACGATCTTGATTATTTGAAGAAATCACTGTCAAATCCTTCAAACCTTGCTTCTTCATTTCATAAGAGCTCTTGTACGAAGCCAGGCTTGGAAGCTGAGGGTGCCCACATGGAAAACATGCAGGAATTTTTGCAGCCAGTATGGAGCAAATTTTCATTGACTGACACGAATATGCAGAAGCACGAAGAGCATGTGAATTTGGGTCAGAAATTGCTTGTGCGTGATAGTGAAGCTGAAGGTTCAGCTACAGCTGTCTGTGAGGATTCTAATAAAATTACGGTGGACAAGGAAATTACTCCAGGAAGTGGAAGAGTGACAGCTAATATTGCCATCGAGGGCTGTGACAAAATTGGTGCCCTTGCCAACTCTCCTAGAGAAGCCATTGACTTCATGGGAGCATCTACGAATCACAGTTCTTTCAATAACGTCGAAATCCACTTTTGTTCTTCTCCACATTTGGATCTTTCCTTGAGAAGATCTCATCCTAGTGGGTTTGAGACTCAAGTTACAGAAGAAAGACACACTCTCCGGCATTCTAATGCATCAGCCTTTACGTGGTACACTAACAGGGCTTCACAGCTTCCACATTCAGCACTGGCAAATACTGGGAATCAGGAAGAATTCAGGGCCAATTATGACGGAAAAATATCCAGTAATGTTAATGGCTACAACTCTGATGCCTTGAGCCTAGCACCAAGTACTCGAAGAAGTGCTATCTCTCTTGCTGCTGGTCAAACCAAGGAATATGAAATTGTAACTTCATCTTCCGGTGAAAAAGTATTTCCGATCCACATTCCTGTGAAAGATACAAGGTTCAATAATCTATGCAACAGTTATGGTGCTGTGCTTCCCCCAATGATGAGTCAAAGCTCAGCTAGCCAGAAAGAACCCATCCATAAAGTGAACCCATTTCAATGTTCCAATTATGGGAGCACCTCTGTACAGCTCTGTGATCGACTTGGTCAAAATGCTAATGATTCCATCAATGGTTCTCTACAAAAGCAAGAAAACAAATTGGATTCTTTGGAAGGTAGAGAACATATTTCTTCCGCCACTGATCAGAGTGCAAGTAGCAGTTTCTGTAATGGAGCCGCAAGTCACTTTAACAGCATAGGCTATGGAAGTGCTTCTGGAAGCTACAGCAATGCTGATCAAATTGCTACTGTCAGTGCTGCTTCTGAGAGCAAGAATGAAGAAGGTGTTTTCACACATAATTCAAACTCTCATCGATCTATTCAAAGAGAAGCAGCTCTAACCAAGTTTCGCTTGAAGCGTAAAGAGAGATGCTATGAAAAGAAGGTTCGGTATGAGAGCAGAAAAAAACTTGCTGAGCAGCGTCCTAGGGTAAAAGGACAATTTGTACGTCAAGTGCACATTGATCCTTCACCTGCTGAAACTGACCAGTAG

>PtPRR7a
ATGGAACTGCTTCAGGTCCAGAACGATGCCCAGGCTGTAATTCAGAGTCAACAGCAACAGTCTCAAGGGCCTTTGGTTATTTGGGAGAGGTTCCTGCCTCTTCGGTCTCTTAAGGTTCTGCTTGTGGAAAATGATGATTCAACTCGCCATGTTGTCAGTGCGTTGCTTAGAAATTGTGGCTATGAAGTTACTGCTGTATCAAATGGTCTACAAGCTTGGAAGGTTTTACAAGATCTGACAAATCACATTGATCTTGTTTTAACTGAGGTGGCCATGCCTTGTTTATCAGGCATTGGCCTTTTAAGCAAGATTATGAGCCACAAAACTTGCCGGAATATTCCTGTTATTATGATGTCGTCTCATGATTCCATGAATGTAGTCTTTAAGTGTTTGTCAAAGGGTGCAGTCGACTTTTTAGTGAAGCCTATTCGAAAAAATGAGCTTAAAATCCTCTGGCAACATGTTTGGAGGAAGTGCCATAGCGCTAGTGGTAGTGGGAGTGAAAGTGCTGTGCGGACTCAAAAATCCACAAAGTCAAATGGTGCTGACGAGTCAGATAACGATACCGGCAGCAATGATGATGATGGCATTGGAAGCGTTGGTTTGAATGCAAGGGATGGAAGTGACAATGGAAGTGGTACCCAAAGTTCCTGGACAAAGAGGGCTGTCGAAGTTGAGAGTCCCAAACCAATGTCACCTTGGGACCAGGACCATTTATCTGATCCGCCTGATAGCACATGCGCCCAGGTTATTCACTCAAGGCCTGAGGCATGTGACAACAGCTGGGTGCCCCTGGCTACAATGAAGAAGTGTGGAGAGCAGGATGATGAACTTGATAATATTGTTATGGGCAAGGACTTGGAGATAGGAGTACCTAGAATTCCAAATTTGCAGCTTAAAGATCCGATCAAAAGGGTACCAACCAACATAGCAGATAATGATGGAGAGAAATTTCCTGAAATAAAATCAAAGCACGATGGTGGGCATTTAGAGAAAAGACAACAGGAGCTCAACAACGTCCCAAACGGTCTCTCCTCTAACCGAAAAAATGAGGTCACCTATGAGACCAAGGAGGTGCCTTCTTTTGAGCTGAGTTTGAAAAGGCTGAGAGATATTGGAGATGCCGGGGCCAGTTCCCATGACCGAAATGTGTTGAGGCATTCAGACCTTTCAGCATTCTCAAGGTATAATTCTGCTTCAACTGCTGATCAGGCTCCAACAGGGAATGTAGGCAGCTGTTCTCCGCTTGACAATAGTTCAGAGGCAGCAAAAACAGAATCAATGCAAAATCTTCAATCTAACTCAAATAGCACACCTCCTAATCAGCGTTCCAATGGAAGTAGTCACAACAATGACATGGGCTCTACAAATAATATTACTTTCGCCAAACCTTCTGTAATCAGTGACAAGCCAACTCTCAAACCTACAGTTAAATGCCACTATCCTTCTGCCTTCCAGCCAGTGCAGAATGACCATACAGCTCTTCCTCAGCCTGTTATACAGGGTAAGGGTGATGCTCCTATTGCTAACACAACTCTGGTTAAATCAAGAGGTGTGAACCAGCAAGGTCAAGTGCAGCACCATAATCATTGTGTGCATAACATGCCCCAGCAGCAGCAGCTCACCAACCATGATGACCTGTCTTTGAATATGACAGCTGCAGCTCCCCAGTGTGGGTCATCTAACATGTTGAGCACACCCACGCAAGGAAATGCTGGTGATTACAGTTTGAATGGAAGTGACCATGGGAGCAATGGCCAGAATGGAAGCAGCATCGCTTTGAGTGGGGCTGTTGAGAAAGGTGGAACCCCTGGACCTGGTGATGAAAGTGGGAGCAGGAGTGGAGTTGGTCGAAATCGCTTTGCACTGAGAGAGGCTGCATTGAGCAAATTCCGCCAGAAGAGGAAAGAGAGATGCTTTGAGAAGAAGGTTCGATACCAGAGTAGGAAGAAGCTGGCAGAACAAAGACCCCGGATTCGAGGACAATTTGTGCGACAACAAGGTGGAGGATAG

>PtPRR7b
ATGCTCTCAATGAACAACGGGTTTGCGGAGCAAAATCATATTGTAGAAGATGAGCAGAAGAAAATAAGGGACGGGATCATGGGTGAGGACCAGGAGCTCTCAGAGGAAGGTGAGTCCCAGATTAATGAGGATGAAAAAGATGTGAATGATAAGGGCATGGAATCGCTTCAGGTCCTGACCGATGCACAGGTTGTTATTCAGAGCCAACACCAACAGTCTCAAGGGCCTTTGGTTCACTGGGAGAGGTTCCTGCCTCGTCGGTCTCTAAAGGTTCTGCTTGTGGAAAATGATGATTCAACTCGCCATGTTGTCAGTGCGTTGCTTCGAAATTGCGGCTACGAAGCTACTGCTGTAGCAAATGGTCTACAAGCTTGGAAGCTGTTACAAGATCTGACAAATCACATTGATCTTGTTTTAACTGAGGTGGCTATGCCTTGTTTATCAGGCATTGGCCTTTTAAGCAACATTATGAGCCACAAAACTTGCAGGAATATTCCTGTAATTATGATGTCATCTCATGATTCCATGAATGTAGTCTTTAGGTGTTTGTCAAAGGGTGCAGTTGACTTTTTAGTGAAGCCTATTCGAAAAAATGAGCTTAAAATCCTTTGGCAACATGTTTGGAGGAGGTGCCACAGCGCTAGTGGTAGCGGGAGTGAGAGTGCTGTACGGATTCAAAAATCCTTGAAGTCAAAAGGTGCTGATGAGTCCGATAATGATACTGACAGCAATGATGACGATGACATTGGAAGCATTGGTTTGAATGCAAGGGATGGAAGTGACAACGGAAGTGGTACCCAGAGTTCCTGGACAAAGAGGGCCGTTGAAGTTGACAGTCCCAAACCAATGTTACCGTGGGACCAATTAGCTGATCCTCCTGATAGCACATTCGCCCAGGTTATTCACTCAAGGTCTGAGGCATGTGACAACTGGGTGCCCCTGGCTACAACGAAGAAGTTTGGAAAGCAGGATGATGAACTTGATAACTTTGTTATGGGCAAGGACTTGGAGATAGGAGTACCTAGAATTCCAAATTTGCAGCATAAAGATCTAAGCAAAGAGGTACTGACCAACATAGCAGGGAATAATGGAGAGAAATTTCGTGAAATAAAATCAGAACAGGACAGTGGGCATTTAGAGAAAGGACAACTGGAGCTCAACAGTGAAAAACACAATACAGAATTGAGAAACCAAGGCAATGATCTCAAGGGTGTCAGCACAAACATTACTAATCCTCAGATTGAAAGTGAAGTCGTAGACATCTCAAACAGTCTCTCTTCTAACAAAAAGAATGAGGTCATCTATGAGACCAAGGAGATGCCTTCTCTTGAACTGGTTTTGAAAAGGCTGAGAGATACTGGAGATGCTTGGGCCAGTGCCAATGACCGAAATGTGTTGAGACATTCAGACCTTTCAGCATTCTCAAGGTATAATTCTGCTTCAACCGCTTATCAGGCTCCAACAGGGAATGTAGGTAGCTGTTCTCTGCTTGATAAATGTTCAGAAGCAGCAAAAACAGAATCAATGCAAAATCTTCAATCCAACTCAAATAGCACACCTCGCAATCTATGTTCCAATGGAAGTAGTAATAACAATGATGTGGGCACTACAACTAATAATGCTTTCGCCAAACCTTTGGTAATCAGAGACAAGCCAACACCAAAATCTACAGTTAAATGCCTCCATCCTTCTTCTGCCTTCCAGCCAGTGCAGAATGACCAGACACTTCATGCTCAGCCTGTAATACAGGGTAAGGGTGATGCTCCAATCGCTAACACAATTCTGGCTCAATCAAGAGGCATGAACCAGCAAGGCCAAGTGCAACACCATCGTCACTGTGTTCATAACATGCCCCTCACCATCCGCAATGATTTGTCTTTGAAAAATATGGCAGCTGCAGGTCCCCGATTTGGGTCATCTAACATGTTGAGCACACCCATGGAAGGAAATGCTGGTAATTACAGTATGAATGGGAGCAATGGCCAAAATGAGAGCTGCATTGCTTTGAATCCCAGAGGAATAAACTTGGAGAGCAATAGTGGGGCAGCTGGAAAAGACGAAAATCCTGGTACAGGTGATGAAAGTGGGAGCAGGAGCGGAGGCGGTCAAAATTGCTTTGCACTGAGAGAGGCTGCATTGAACAAATTCCGCCAGAAGAGGAAGGAGAGATGCTTTGAGAAGAAGGTTCGATACCAGAGTAGGAAGAAGCTGGCAGAACACAGACCCCGTGTCCGAGGACAATTTGTGCGACAAGTACCATTTGAACATAAAGATGAGGATGCACAGAGCTAA

>PtPRR9la
ATGGGTAAGGTGGTGTTGAGTAGCAGTAGTGAAGAGGCGGGGGGGATGGTGGTGGAGTTAGAGACAGAGAAGAAGGATATTGGATCGTCGGAGGTGGTTCGATGGGAAAAGTTTCTACCAAAGATGGTGCTCAGGGTTCTTTTGGTCGAAGCGGATGATTCTACTCGTCAGATTATTGTTGCTCTTCTCCGAAAATGCGGTTACAGAGTTTCTGCTGTTCCTGATGGATTAATGGCGTGGGAGACTTTGAAGGAGAGACCCCATAGCATAGATCTCATATTAACTGAAGTGGAGTTGCCATTAATATCGGGATATGCGTTTCTTGCTTTGGTCATGGAGCATGATGTTTGCAAAAACATTCCTGTCATAATGATGTCTTCACACGATTCAATTAGTGTGGTTCTGAAGTGCATGTTAAAAGGATCAGCTGACTTTCTCGTTAAGCCTGTTCGGAAGAATGAATTGAGGAACTTGTGGCAGCATGTTTGGAGAAGGCAAACTCAAACTGCTGGAAAAATCCCTCGAAACTCGAATAGAGTTGAGGCCTCATCTGAAAACAACGCAGCTTCGAGTGATTTTGCGACGTCTTTGCAGAAAAATAAGGATTGCAGCGAGAAAGGGAGTGATGCCCAAAGCTCTTGTACGACCCCTTGCTTGGAAGCTGAGAGTGCTCACATGCAAAATATACAGGGACTTTCATATCTGAAGTATAGGAGTGCTTCAAACTTGAGCGATGCAGATAATGAGAAGTATGAAGATTATGCCAAATTAAATAAAAGCCCAGTAAATCCTGAGAGCAAGACCGGAGTATTTGTAGCAGAAAGGTCAAACAGGACGAGACCTGACAGAGAACCGTACCATGGAGCTTATAATCCAACTGCTTCGAGACTGGTAGAGGAGCATGCTTGTGCTAAGTCAGCGATTCATGATGAGAACTCGAGACCAGAAAATGATAGGGAACATGCTAATAGCTCCTTTGGCCACGATGATGTACTTGCTGAAACCTCAAGTGGAGCCATTGACTTGATTGGTTCCTTCAATAATCAACCAAAGCACACATATGCATACTCTAGTTTACATGATGCGACAAACAAGTTTGAATTCCCTCCACTACTTGAACTCTCTCTCAGAAGATTGTATCCCAGTAGCTCAAAGAACCAAGGGCTAGATGAAAGACATGCATTGAACCATTCCAATTCCTCAGCCTTCTCACTGTACAATAGTAAGACACTGCAATCCCTTTTTCCAACATCTGCCAGTAATGGCTCAGATTCCAAGGAAGAAGCCAGTAAGTCTCCTGATCCGTCATCCAATCAACTTGCTCAAAATGTTGGTACTCTTTCTCAGATACACGATGCCTCTTTGAGTGGCAATCAAGAAATTATTACCACTCCGGTCATTGGCCAGTCTGGGAAGGTGGAATTAGCACATCCAAGCCCTCAACTTGGATTGATTCCTGTCTTAGGTACAAGGCTTGACAATATCTCTACCGGATGTGGTCATGTTTTCTCCCCTCTATGTTATACACAATCAAATGCAGCATGGAATCCCAATCTTGCGGGGCGGCAACAGTCTCCATTTCCTACAACTGCCTCAGTTCATTCAAATCCTGAAGTTCTTGATTCCAAGCAAAACCACAAGTGTTATGTTGACCAAAATGATCTTCAGCAGAACAATAGGGAACCTGTGGATGAAATGAGACATGATTCACCTGCTGCTGGTCAGAGTACTAGTAGTAGTTTATGTAACCGTGTTGCCAATAATAATAGTAGCAGTGCTTATGAAAGCTTTGGCAGTGGAAACGATGTAAATGCCTCTTCGGTTGGGACAGCTGAGAAGTCCATGGCTCAAGAGAACTTGAATAATGGGGGTAACTTCAACCATGATGGTTTTGGAGGGAGCGATTCCTATCGCTCCAGCCAAAGAGAAGCTGCTCTAACAAAGTTCCGCTTGAAGCGGAAAGATCGATGCTATGAGAAAAGGGTTCGATACCAAAGTCGGAAAAGACTGGCAGAGCAACGTCCTCGGGTGAAAGGTCAGTTTGTTCGTCAAGCGCAAAATGATTGCCCAGTTGCTAATGGTTGA

>PtPRR9lb
ATGGGTGAGGTTGTGGTGAGTAGCAGCAGTGAAGAGGTGGAGGGGATGGCGGTGGAGTTAGAGACAGAGAAGAAGGATATTGGATCATCGGAGGTGGTTCGATGGGAAAAGTTTCTACCAAGGATGGTGCTCAGCGTTCTTTTGGTTGAAGCTGATGATTCTACTCGTCAGATTATTGCTGCTCTTCTCCGAAAATGCAGTTACAGAGTTGCTGCTGTTCCTGATGGATTAATGGCATGGGAGACTTTGAAGGGTGGACCTCATAACATAGATCTCATATTAACTGAAGTGGAGCTGCCTTTAATATCAGGATATGCACTTCTTACTTTGGTCACGGAGCATGCTGTTTGCAAAAACATTCCTGTCATAATGATGTCTTCACAAGATTCAATTAGCATGGTTCTGAAGTGCATGTTAAAAGGAGCAGCTGACTTTCTCATTAAGCCTGTTAGGAAGAATGAATTGAGGAACTTGTGGCAGCATGTTTGGAGGAGGCAAACTCTAAGTGCTGGACAAATCCCTCAAAACTTGCATAAAGTCGAGGCCTCATCTGAAATCAACGCTGCAAGCAATGGTTCGAGTGACTCTGTGATGTCTTCGCGGAAAAATAAGGATTGCAGCGAGAAAGGGTGTGATGCCCAAAGCTCTTGTACGACCCCTTGCTTGGAAGCTGAGAGTGCTCACATGCAAAATATGCAGGGACTTTCACAGATGAAGTATAGGAGTGCTTCAAATCTGAGCAACACAGATAGAGAGGAGTTCGAAGAGTGTGCCAAATTAGATAAAAGCCCTGTAACTCCTGAGAACAAGACTGGAGTATTTGTACCAGAAAGGCCGAACCGAATGGAGTCAGATGGAGAACCGTGCAGTGGAGCTTATAATCCAACTTCTTTGAGACTGCTAGAAGAGCACGCTTGTGCCAAGTCAGCAATTCAAGATGAAAATTCGAGGCCAGAAAATGACAGGGGGCTTGCTAATAGCTCCTTTGGCTGCGATGATGTACCTTTTGAATCCTCAAGTGGAGCCATTGACTTGATTGGTACCTTAAATAATGGTCCAAAGACCACATATGTACACTCTAGTTTACATTATGGCACAAACAAGTTCGAATTTGCTCCACAGCTTGAACTCTCTCTCAAAAGATTGTATCCCAGCAGCTCAAAGAACCAGGGGGTAGATGAAAGACATGCACTGAACCATTCCCATGCCTCAGCCTTCTCATGGTACAATAGCAAGACATTGCAACCTCCTTTTCCAGCATCTGCCAGTAATGGTTCTGATTCCAAGGAAGAAGCCAGCAAGTCTCCTGAGCTGTCATCCAATCAACATGCTCAAAACATTAATTCTATTTCTCAGAGACATGGTGCCACTTTGAGTGGGAATCAAGATATGACCATTCCAATCATTGGCCAATCTGGGAAGGCTGAATTAGCATATCCAAGCCCTCGACATGGATTGATTCCTGTTCGAAGGGGGATGCTCGACAATATATCTACCGAATACGGTCATGATTTCTCCCCTCTATATTATACTCAATCAAGTGCAGCATGGAGTCCCAAACTTGCTGGGTGGCAACAGTCTCCATATCCTCTTAGTACCTCAATTCATTCAAATCCTGATATTCATGATTCTGAGAAAAATCACAGGTGTTCTGATGAAACTACTTACAACTCTGTTGACCAAAATGATCATCAGCAGAACAATAAGGGACCTGTGGATGAAGTGAGACATGACTCACCTGCTGCTGGTCAGAGTACTGGTGGTTTATGTAATGGTGCTATCAATCATAATAAGAGCAGTGCATATGAAAGCTTTGGCAGTAGAGATGATGGAAATGCCAAAGAGAAGGCCATGGCTCAAGATAATTTGAATGATGGGGACAACTTCAATCGTGATGGTTTTAGAGGGATAGATTCCCTTCGCTCCAGCCAAAGAGAAGCTGCTCTAACAAAGTTCCGACTGAAGCGGAAAGATCGATGCTATGAGAAAAAGGTTCGATACCAAAGCCGGAAAAGACTGGCAGAGCAACGTCCTCGGGTGAAAGGACAGTTTGTTCGTCAAGTGCAAAATGATTCCCCAATTGCTAATGGTTGA


Vitis vinifera
>VvPRR1/TOC1
ATGGAGATGGCGCGGAATGAGATTGGCAGCGGCGGAAGTAACAGTAAGAGCAGTGATTCCTTTATCGATCGAAGCAACGTGCGGATTTTGTTGTGCGACAACGATACCAAGAGTTCGGACGAGATCTTCACTCTTCTCTGCGGGTGCTCCTATCAAGTAACATCAGTGAGGTCAGCTAGACAGGTGATTGATGCATTGAATGCTGAGGGACATGATATTGATATCATACTCGCTGAAGTTGACCTTCCAATGACCAAAGGCATGAAGATGTTAAAGTACATTATGCGGGATAAGGAGTTGCGACGCATACCCATTATCATGATGTCTGCACAGGACGAGGTTTCTGTTGTTGTGAAGTGCTTGAGACTTGGTGCAGCTGACTACCTTGTAAAGCCTTTGCGCACAAATGAACTATTGAACTTGTGGACTCACATGTGGAGAAGGCGGCGAATGCTTGGACTTGCAGAGAAGAACATCTTGAATTATGATTTTGATGTGGCAGCTTCAGACCCTAGTGATGCTAATACAAACAGTACTATGTTCTCGGATGACACGGATGACAAGTCACGGATGAGTGCCAATCCAGAGATGGTTGTTTCAGTTCATCAGGAAGATGAGTCTAATGCTACTATTGATAGCACTGATGTTGCTGCTGAGCCTCCAGTTGTGAATCAGTTGGAATGTCGGCCTGATGTTCCAGGAATTAGTGACAGGCGAACAGGACAACTTCTTTCAGGCCCAAAAAAGAGTGAACTGAAGGTTGGTGAGTCTTCTGCCTTCTTTACATATGTCAAATCAAGCATGATTAAAACCAATTCTCAAGGGATTCCCAACATCAATGAAAGTGCTCCTCAACATTCAAGGATGGAAGAGAGAGTTCAAGCATGGAGTGAAAAGGGGGTTAATGATACCCAAGAACATGAAAATGGAGAGGCATGGGAAAACTATTCACAAGGAGATGACTTTCCAAGCAAAGAGTTCTCTCAGGTGCCAATTCATCCAAGAAATGAACATCAAGTTGATATTTCAGGGTTTCCGGGGCACACTGCTTACCCATATTGTATGTCAGGAGTGATGAATCAAGTGATGATGCCATCATCTGCACAGCTGTATCCGAAGAGCCTGCATGACATGCAAAATAATGCTACTACTTCTGCCATGCTACCTCAGTACAATCATCTTCCACAATGTCCTCCCCATGTGCCTGGAGTTGCATCATTTCCTTATTATCCAGTTAGTATATGCCTGCAACCAGGTCAAATGTCCACTACACACCCCTGGCCATCATATGGAAATTCATCTTCCACTGAAGTGAAATTAGGTAAAGTAGATAGAAGAGAGGCTGCCTTGATAAAGTTTAGGCAGAAAAGGAAGGAGCGTTGTTTTGACAAGAAAATTAGATATGTGAACAGAAAACGACTTGCTGAAAGGAGGCCACGTGTGAGAGGACAGTTTGTGAGGAAAATGAATGGTGTAAATGTGGATCTTAATGGGCGACCTGCTTCTGTTGATTTTGATGAGGATGAAGAGGAGTATGAGGAGGAGAATGCATCAAGGGATTCTACTCCTTGA

>VvPRR3
ATGGGGTTTGTTCAAGCAAACAATGATGGTGGTGGGGAGGGAGGGTTGGTGAATAGAGAGAACAGTACATGGGATGAGGGAAAAGAAGTTAGGAATGGAATGGTGGGTGAGGGGCAAGGCTTGGGCTCATCTGAGGAAGATGAGCCTGGGAGCAATGAGATAATTGAAGATGCAAATAAGGGGTCGGAAGAAGCAATCCAAGTCCATGATGGGCTGCAGAATTCGCAGCAACAGCCTCAAGGGTCAGCAATAAATTGGGATAGTTTTCTCCCCATTAGATCACTGAAGGTTCTTCTGGTGGAAAATGATGATTCGACACGCCATGTAGTTACTGCTCTGCTTCGGAATTGCAGTTATGAAGTTACTGCTGTTGCAAACGGTCTTCAGGCATGGAAAATCTTGGAAGATTTGACCAATCATATTGATATCGTCCTAACAGAGGTAGTCATGCCCTTTATATCGGGAATTGGTCTTTTATGCAAGATCATGAGCCACAAAACTTTCAAGAATATTCCTGTGATTATGATGTCATCCCATGATTCTATGGGTATAGTCTTTAAGTGTCTGTCCAAGGGTGCAGTTGATTTTTTGGTGAAACCTATTCGAAAGAATGAACTTAAAAACCTTTGGCAGCATGTTTGGAGAAGATGCCACAGTTCTAGTGGTAGTGGGAGCGAAAGTGGCACACAGACTAAAAAATCTGTGAAGTCAAAAAGCAATGATGAGTCTGAAAACAATACGGGCAGCAGTGATGAGCGTGATAATGGAAGTACTGGCCCAAGCATTCGGGATGGAAGTGACAATGGAAGTGGTACCCAGAGCTCATGGACAAAAAGAGCAGCTGAAGTTGACAGCCCCCAACCATTATCACCATTGGATCAATTGGCTGATGCACCTGATAGCACTTGTCCCCAGGTAATCCATACAAAGCCTGGAACTTTAAGCAACCAGTGGGTGCATGTTATTGAAACAAAAGAATGCCAGGGCAAGGATGAACAACCTGACAATGTTGCAATGGGTAGAGACTTGGAATTAGGAGTAGCTACAAATCCAGCTGTACAACTTGAATATCAACATGAGAAGTTTTCAACCTACCCAACATGCAAGAGGCAGAATAAATTGCCTGAATCAGATAGTAAACCATTTGATAAAGAACACTTGGAACATAACAGTACAAGTGCCAATTGTACTGACCCACAGGTAGAAAGCAGAACTTTTGAGAATCCAAATGGCCTTTCTGATGTGTCACAGATCAAAGACAAGGGAAGCTGTGAAACTACAGAACTGCCACCCCTTGACTTAAGTTTAAAAAGGCTGAGAGGAGCAGGAGATGTTGGGGCCTGTGTGCATGATGATCGCAGTATTTTAAGACATTCAGATCTTTCAGCATTCTCAAAGAGTAATACTACCTCCTCTGTTAAGCAGGCTCCCACAGGGAATGTAGGAAGCTGTTCTCCACTCGATAATAGCTCAGTTGCAATGAAGACAGAAACAATGCATAATTTCCCCTCTCATGTGGATGGCATTCCTCCAAATCAGCAGTCTAATGGCAGTAGCAACAACATTGACATGGTCTCTACCACCAAATATGCTATTCCCAAGTCAGAAGCTTACAACGAGAAATCAGAGTCAACGTCAGCATTTAAATGTTTCCATTCATCTGCCTTCCAACCTGTAACTAGTGGGCGAATGTTTCCACCACAGAAAGTCTCATCTGGGAAGGCAGATGATGTGGAGCAGCAGCAGACTGATAATGACGATCTCTTTTTGAAGAACATGGCAGCAGCAGCTCCACAATGTGGATCATCAAATGTGATTGGTGGACCCACTGAAGGCAATGCTGGAAACTACAGTGTAAATGGGAGTGCTTCGGGAAGTAACCATGGGAGCAATGGGCAGAATGGGAGCAGCACTGCCTTGAACGTGGGTGCAACAAACATGGAAGCTGGTGCCATCAGCGGAAAGGGCGGTGGAAATAGAGTTGAAGAAGACCGGTTTGCGCAGAGAGAGGCTGCCTTGACTAAATTCCGTCAGAAAAGGAAAGAGAGATGCTTTGAAAAGAAGGTTCGTTACCAAAGCAGGAAGAAACTGGCAGAACAACGACCACGTATTCGAGGACAATTTGTCCGGCAAAATGTGTATGCTAACACAATTCCATAA

>VvPRR5
ATGGGTGAGGTTGTGGTGAGCAGTGAGGCAGGAGGAGGAGGCATGGAGGGTGAGGTGGAGAAGAAGGAGGTGGGCAGTGGGGTTGTGAGGTGGGAGAGGTTTCTTCCCAGAATGGTTCTCAGGGTTTTGTTGGTTGAAGCGGACGATTCCACCAGGCAAATTATCGCTGCGCTTCTCAGGAAATGCAGTTACAAAGTTGCTGCTGTTCCTGATGGCTTAAAGGCATGGGAGGTACTGAAGGCTAGACCCCACAACATTGACCTCATATTGACAGAAGTGGAGTTGCCATCAATATCTGGCTTTGCTCTCCTCACCTTGGTTATGGAACATGAGATCTGCAAAAACATTCCTGTTATAATGATGTCCTCACATGGTTCGATAAACACGGTTTATAAATGCATGTTGAGAGGTGCAGCTGACTTTCTTGTTAAGCCTGTTAGAAGAAATGAGCTGAAGAATTTGTGGCAACATGTCTGGAGAAGACAATCGTCAACTGTTAGCGGAAATGGCCCCCAAGATGAGAGTGTTGCACAACAGAAGGTCGAAGCCACTTCTGAAAACAACCCCACAAGTAATCACTCAAGTGATCATGTTGCTTGTATTCAGAAAAATAAGGAAGCACTCAATAAAGTGAGTGATGCTCAGAGCTCTTGTTCAAAGCCAGACTTGGAAGCTGAGAGTGCCTACATGGAAACTATGCAGGATTTCTCAAATCCGACATGGAGCAGATCTCTTGTGAGTGACACAAAAATGCAGAAGAATGAAGAATGTGCCAAATTGGGCCCGAAATTTCTTATGCACAATAAAGAAGCTGGGGGAACACTGGAGGCTGCCTGCAGGGATGTGAACACAATGACTCAGCCTGAAGCAGTGGAACCAGAAAATGATGGGCAAGGTGCTAACGCTCCTAGTGAGGCTTGTGGTAACAATGCCATATTGGGCAGCTCATCTAGAGAAGCCATCGACTTGATTGGAGTATTTGATAATTCTAAAAAATGCACTTATGGAAATTCTTCTTCAAATAATGGCACCAAAAAGAGTGATTCTATTCCACAGTTGGACCTTTCCTTGAGAAGATCTCATCCTAGTAGCCCTGAGAATCAAGTTGCTGATGAAAGGCATACACTGAACCATTCTAATGGCTCGGCCTTTTCACGCTACATAAACAGGTCATTGCAGCCACCACATCTACCATCAACAGGTGTTTTCAATCAGCAGAAAAACTTTGGAGCTGATTCTGATAAACGTTTATCTCAGCTGGTTACTGGTTATAACTCTGATATTACTATTCCAGTGAGAGGTGTAAGGTTTGATGCCAGACTAATCACAAGCCAGAGCACAAATTGGACTCACTGGAGGGCCGCTGCAGAGGGCAAGAATGAAGAAGGTATCTTCAGTCATGAAGGACACTCTCAACGATCTATCCAAAGAGAAGCTGCTCTAACCAAGTTTCGCTTGAAGCGGAAAGACAGATGCTTTGAGAAGAAGGTTCGTTATGAAAGCAGAAAGAAGCTTGCAGAGCAGCGACCCCGAGTAAAAGGACAGTTTGTTCGACAAGTGCATACCATCCCCCCACCTGCAGAGCCTGATACATACTATGGCAGTTCGTTTGATGGTTAG

>VvPRR7
ATGAATGTTAACGGTCCTTGGACCAAAGGGTCAGCCGAACTTAACCACCATGCACAAGATGAGCAAAAAAATATAAGGGACAAAGTTGTGGGTGAGGGCCATGGCCTATCCGAGGAAGATGAATCGAGGATTAATGAGGATGTGGAAGATCTGAATGATGGGCGAATAGGGTCAGTCCAGGCAGTCCAGGGCGTTCTGCATGGACCACAGCAACAGCCTCAAGGGCCTATTGTTCGTTGGGAAAGGTTTCTCCCCCTGAGGTCTTTGAAGGTTCTACTGGTGGAAAATGACAATTCAACTCGCCAGGTTGTCAGTGCATTACTTCGAAATTGCAGTTATGAAGTTACTGCTGTAGCAAATGGTGTACAAGCATGGAGCATCTTAGATGATCTAACCAATCATGTAGACCTTGTTCTGGCCGAGGTAGCATTGCCTTCTTTATCAGGCATTGGCCTTCTTTGCAAGATTATGAACCATAAAGCATGCAAGAATATCCCTGTGATTATGATGTCATCCCATGATTCTGTGGGCATAGTCTTTAAATGTTTGTCAAAGGGTGCAGTTGACTTTTTTGTGAAGCCTATTCGAAAAAATGAGCTTAAAAACCTTTGGCAGCATGTGTGGAGGAAATTCCACAGGTTTAGCGGTAGTGAAAGTGAAAGTGAAAGTGGTATACGGACTAAAAAATCTGCAAAGTCAAAGAGTGTTGCTGGGTCAGACAACAACACTGGCAGCAATGATGAGGATAATGGAAGTATTGGTTTGAATGTTAGGGATGGAAGTGACAATGGAAGTGGTACTCAGAGTTCTTGGACTAAAATGGCAGTAGAAGTTGACAGCCCCAAACCAATGCAACCATGGGACCAATCGGCTGATCCTCCAGATAGCACTTGTGCACAGGTTACCCAATCATGGCCTGAAGCATTTGGCAACTACCAGGTGCCCATGACTTCCTCCAAGGATTACCAAGAACAAGATGATGAACTTGATAATGTTGAAATGGGCAAAGACTTGAAGATAGGAGTACCTAGAAACTCAAATTTACAGCTTCAAGATGATGTAGTAGGCGCTAATAAGGATAAATTTCATGAATTGACCTTGAAGAAAGATGATGAAAAATTAGAGAACAGACAAATGGACCTCAACAGTAACAAGCCAAATGATGAATTGGATAAAGAAGCTGTCGATCTGATGAGTGTCATTGCCAACAATACCAATCCTCAGAAAAAAAGTATGGGCTTCAAAACTCCAAGTGGCCTCTCTGAGGTCCCTGAAACTAAAGATAAGGCCATGTACGACAAAAAGGAAATACCTTCCCTTGAGCTCAGTTTGAAGAGGCTGAGAGATACTGGAGGTACTGATACCAATCCCCATGACCAAATCATTTGGAGACATTCCGACCTTTCAGCCTTTTCAAGGTATAATTCTGCTTCAACTGCTATTCAAGCTTCAACTGGCAATGTAGGTAGCTGTTCTCCACTTGATAACAGCTCAGAAGCAGCAAAGACAGAATCCATGCAAAATTTCCAATCTAATTCGAATGGCACTCCTCCCAACCAGATCAAACATCTCCAGCACTCTGCTTTCCAACCAGTACAGAATACAATTTTGGCCGATTTTGCAAATGCCAATACAATTTTGGCCCACCCAAGTGCCATGCCCCCACAGGTCCAAATCCAGAACCACCATTACTATTACCACCACCATGTCCATAATATATCACAGCAACAGATACGCATCCATGATGATTTGGCTTTGACCAACATGGCAAAATCTGCTCCACAGTGTGGGTCATCCAATGTGTTGAATGCGCCTGTTGAAGGTTATGCCTGTAATCACAGTTTGAATGGGATAACCATGGTAGCAACGGCCAAAATGGGAGTACCACTGCTTGATGATGGAATAGCTGGAAAAGGTGGAGCAGGTGGAGGAAGTGGGAGTGGAATAGATCAAAACCAATATGCACAAAGAGAGGCTGCATTAAACAAATTCCGCCAGAAAAGGAAAGAACGATGCTTTGAGAAAAAGGTACGATACCAAAGCAGGAAGAGACTGGCCGAACAGAGACCACGCATTCGAGGACAATTTGTTCGTAGGGTATTCCATGATATTAACAGCGAGGATGCAGACAGCTAA

>VvPRR9l
ATGGAGGGGAAGGAGGAGAAAGAGAAGAGTTCATCGGGTGTGGTGAGGTGGGGGAGGTTTCTTCCTCGGATGGTTCTGAGGGTTTTGTTAGTGGAACCTGATGACTCCACTCGTCAGATTATCGCTGCTCTTCTTCGGAAATGCAGTTACAAAGTTGCTGCTGTTTCTGATGGCTTGAAGGCATGGGAGGCACTGAAGGGGGAACCCCAAAATGTTGACCTTATATTAACAGAAGTGGAGTTGCCATCAATATCTGGATTTGCACTTCTGTCATTGATAATGGAGGATGACATTTGCAAGAAGATTCCTGTCATAATGATGTCTTCTCATGATTCAATTAGCATGGTTTTGAAATGCATGTTGAAAGGGGCCGCTGACTTTCTTGTCAAGCCTGTTAGGAAGAATGAGCTGAGGAACCTATGGCAGCATGTTTGGAGAAGGCATGCACCAACTAGTGGGCATGTGTCTCAAAATTTAAGCATTGCACAGAACAAAGTTGAAGTCAGTTCCGAAAACAATACAGCAAGCAATCACTCGAGTGATTATGTGGTTTCTGCACAGAAAAAGAAGGAATGTAGTGAGAAAGGGAGTGATGTCCAAAGCTCTAGTACAACGCCTTACTTGGAAGCTGAGAGTGCATACATGGAAAATATGCAGGGATTTTCACAGTTGAAATGCAGGAGTGTATCTAACTTGAGCAATGAGGAGATAAGGAAGCATGAAGACTGTATTGAAGTGACTAGTGGGGATGGTGTGGGACCTGCAAGCCATATAGAAAACACTGATATTACTGGTGAGAATCATGGTTGCAATGAAAAATGGATTGAACCTTCTAGTGGGGCCATTGACTTGATAAGCACATTTGATAATTACCCAAAGGACCGTAACCAATTTTCTAGTTCCAATGATGGCATCAGCAAGGATGGTTTTGCTCCACAGTTGGAACTTTCTTTGAGAAGATTCCAACCCTGTAGCTCAAAGAACCATGGAAGTGATGAAAGGCATACACTAAACCATTCCAACTCTTCTGCCTTTTCATGGTATAATAATGGGAAGTCATTGCAACCCCTTTTTCCAACATCGGCCATTAATTGTAGTGAACTGAAGGAGGATGCAAGTTATTCTCATGAACGTTTATTCAATCAACTCCCTGAAAGTACTGTTGGTACTTCTGAGAGTGCTAGTAGTAGTTTATGCAACGGTGTGGTAAGTCATCTTAGCAGCAGTGTGCATGGAGGCATTTGCAACAGAAATGATGGCAATCCCACTTCAAATGGTGCCGTTGTGAGGACTACTGCTCCTGAAAGGATGGATTCCCATCATTCCACCCAACGTGAAGCAGCTCTAATGAAGTTTAGATTGAAACGGAAAGATCGTTGCTTTGAGAAGAAGGTTCGATACCAAAGCAGGAAAAGGCTTGCAGAGCAGCGTCCCCGAGTGAAAGGACAGTTTGTTCGACAAGTGCAGACTGACACTCCAACCGCTGATGGGTAA


Oryza sativa
>OsPRR1/TOC1
ATGGTGGGCGCCGGCGAGGGGGATCGCGTTGGCGGCGGGGCGGCGGTAGGGGGAGGGCAGCAGTTCGTGGACCGGAGCAAGGTGAGGATTCTGCTCTGCGACAGCGACCCAAGTAGCTCACGGGAGGTGCTCCGCCTCCTCTGCAACTGCTCATACCAAGTGACTTGCGCCAAGTCTCCGAGGCAGGTGATCAACGTACTCAACTGCGAGGCGGGGGAGATCGACATCATCTTGGCCGAGGTCGATCTGCCCGTCTCCAAGTGCTTCAAGATGCTCAAATACATCGCCAGGAACAAGGAATTGCGCCACATCCCCATCATAATGATGTCCAACAGAGACGAGGTCTCTGTTGTTGTCAAGTGCTTGCGTCTCGGGGCAGCCGAGTACCTGGTCAAGCCACTGCGCATGAACGAGCTGCTGAACCTCTGGACCCATGTGTGGCGGCGAAGGCGGATGCTTGGTTTGTCGGAGAAAAACTTCTTCAATGACAATTTCGAGTTGGCGTTATCGGAACCTAGTGACGCCAATACCAACAGCACCACTCTTCTCTCGGATGACACAGATGATAAGCCAAAAGAAAACATTAATCAAGAAACAAGCACCTCGAATCAACATGAATATGAGTCTAATCCTTCTGATGCTGAGCCTAAACAAAAAGGCACACCAGAGGGTTTACTAGTCTCTACTGAAGGCGGCGACCAAGCTTCATCTCCCGGAGTAATGTTTTCACGTCCAATAAAGACTAACTTGAGGGTTGCTGAGTCTTCTGCTTTTCTAGCTTATGTTAAGTCAAGCACCCCAACTACCAGCTCATTTGACAGCGAACTACAAAAAGGTGGAAATCGGTTAGACTCTTCGGATCACAGGGGTAATTTCTCTAGTACAACTGACAGAAGTGACACTGGCACTGATGTAAATATTCGGGATAAAGAAGCCTTTGAGATGCCAGTGCAATACCCTGTGGTATGCTTTTCTTCCTCTAACTTGCATCTGGAGCGAAGCAATGAAGGCCAAAATGATGCTTCAGGAACTCCTCCTGTATATCATTTTCCCTTTTATTACCCAGGGATGATGGACCACGGTATGACACATCCTCCGGTGCAAAATTTCCAAGGGAACATAAACAACGCTCAAGTGCATACACCACAAACGTTGCTCCCTCAGTATAATGTTTATCCCCAATGTCATGGTGTATCTATGATGCCACCATTTCAGTATAATCCTGCTGGTATGAGCATTCAATCAAATCAACTGCCAACACAAAATATGTGGCCACAGGCATCAAGCACACCAATGCCCGAGGAAACATGTAGTCGGTCTGAACGGAGAGCTGCAGCACTTGCCAAATTCAGGCTGAAAAGGAAGGAACGTTGTTTTGACAAGAAGGTGAGGTACGTGAATAGGAAGAAACTTGCTGAAACAAGGCCGAGGGTGCGAGGGCAATTTGTTAGACAGGCAAACTATACAGATATAACCAGCACTGGAGATGATATCTCAGAAGATGAAGATGATGATCCATCCTCCAGGGAGGTAGAGATGGTTTCTTCTCCAGAGTAG

>OsPRR37
ATGATGGGAACCGCTCATCACAACCAAACCGCCGGCTCTGCCCTCGGAGTCGGAGTCGGAGATGCCAACGACGCCGTGCCTGGGGCTGGGGGTGGGGGCTACAGCGACCCGGATGGCGGACCAATCTCCGGTGTGCAGCGGCCACCGCAGGTCTGCTGGGAGCGCTTCATCCAGAAGAAGACTATCAAAGTCTTGCTAGTTGATAGCGATGACTCCACCAGGCAGGTGGTCAGTGCCCTGCTTCGTCACTGCATGTATGAAGTCATCCCTGCTGAAAATGGCCAGCAAGCATGGACATATCTAGAAGATATGCAAAACAGCATTGATCTTGTTTTGACAGAGGTTGTTATGCCTGGTGTATCTGGAATTTCTCTATTGAGTAGGATCATGAACCACAATATTTGCAAGAATATTCCAGTGATTATGATGTCTTCAAATGATGCTATGGGTACAGTTTTTAAGTGTTTGTCAAAGGGCGCTGTTGACTTCTTAGTCAAGCCCATACGTAAGAATGAACTTAAGAACCTATGGCAGCATGTGTGGAGACGGTGCCACAGCTCCAGTGGCAGTGGAAGTGAAAGTGGCATTCAGACACAAAAGTGTGCCAAATCAAAAAGTGGGGATGAATCCAATAATAACAATGGCAGCAATGACGATGATGACGACGATGGTGTAATCATGGGACTTAATGCAAGAGATGGCAGTGATAACGGCAGTGGCACTCAAGCGCAGAGCTCATGGACAAAGCGCGCTGTTGAGATTGACAGTCCACAGGCTATGTCTCCAGATCAATTAGCTGATCCACCTGATAGCACTTGTGCACAAGTGATCCACCTGAAGTCAGATATATGCAGCAATAGATGGTTACCATGTACAAGCAACAAAAATTCCAAGAAACAAAAAGAAACTAATGATGACTTCAAGGGGAAGGACTTGGAAATAGGTTCTCCTAGAAATTTAAACACAGCTTATCAATCCTCTCCGAATGAGAGATCCATCAAACCAACAGATAGACGGAATGAATATCCACTGCAAAACAATTCAAAGGAGGCAGCGATGGAAAATCTGGAGGAGTCAAGTGTTCGAGCTGCTGACTTAATTGGTTCGATGGCCAAAAACATGGATGCACAACAGGCAGCAAGAGCCGCAAATGCCCCTAATTGCTCCTCCAAAGTGCCAGAAGGGAAAGATAAGAACCGTGATAATATTATGCCATCACTTGAATTAAGTTTGAAAAGGTCAAGATCGACTGGGGATGGTGCAAACGCAATCCAAGAGGAACAACGGAATGTTTTGAGACGATCAGATCTCTCGGCATTTACGAGGTACCATACACCTGTGGCTTCCAATCAAGGTGGGACAGGATTCATGGGAAGCTGTTCGCTGCATGATAATAGCTCAGAGGCTATGAAAACGGATTCTGCTTACAACATGAAGTCAAACTCAGATGCTGCACCAATAAAACAAGGTTCTAATGGTAGTAGCAATAACAATGACATGGGTTCCACTACAAAGAACGTTGTGACAAAGCCTAGTACAAATAAGGAGAGAGTAATGTCACCCTCAGCTGTTAAGGCTAATGGACACACATCAGCATTTCATCCTGCACAGCACTGGACGTCTCCAGCTAATACAACAGGAAAAGAAAAGACTGATGAAGTGGCTAACAATGCAGCAAAGAGGGCTCAGCCTGGTGAAGTACAGAGCAACCTCGTACAACACCCTCGCCCAATACTTCATTATGTTCATTTCGATGTGTCACGTGAGAATGGTGGATCCGGGGCCCCTCAATGTGGTTCATCCAATGTATTTGATCCTCCTGTCGAAGGTCATGCTGCCAACTATGGTGTCAATGGAAGCAACTCAGGCAGTAACAATGGAAGCAATGGGCAGAATGGGAGTACGACTGCTGTAAATGCTGAACGGCCAAATATGGAGATCGCTAATGGCACCATCAACAAAAGTGGACCTGGAGGTGGCAATGGAAGTGGAAGCGGCAGTGGCAATGACATGTATCTGAAACGCTTCACTCAACGAGAGCATAGAGTGGCTGCAGTGATCAAGTTTAGACAGAAAAGGAAAGAGCGCAACTTCGGAAAAAAGGTGCGGTACCAGAGCAGAAAGAGGCTGGCCGAGCAGCGGCCAAGGGTCCGCGGACAGTTCGTGCGGCAAGCTGTGCAAGACCAACAACAGCAGGGTGGTGGGCGCGAAGCGGCAGCGGACAGATGA

>OsPRR59
ATGTCTCCCGACGCCGACGCGGCGGCGGCGGCGGCGGCCGGCGGCGAGGGCGCGGCGGCGGCGGGGGTGGGGACGGCGGGTGAAGGGCGGGGGGTGATCCGGTGGGACCAGATCCTGCCGCGGCGGTCCCTCCGCGTGCTGCTCGTCGAGCACGACGACTCCACCCGCCAGGTCGTCACCGCGCTCCTCCGCAAGTGCGGCTACCGCGTGGCGGCGGTGGCGGACGGGATGAAGGCGTGGGGGGTGATGCGGGAGCGGGCGTACGCCTTCGACCTCGTGCTCACGGAGGTCACTATGCCCACGCTCTCCGGCATCGAGCTGCTCTCCAGGATCGTCGCCTCCGACGAGTGCAAGAACATCCCCGTCATAATGATGTCGTCCCAAGATTCTATTGGCACAGTGCTCAGGTGCATGCAGAAGGGTGCGGTGGATTTCCTTGTGAAACCAGTGAGAAAGAATGAATTGCGTAACTTATGGCAACATGTATGGAGGCGACATGCAATGAATAGCCAAACAAATGCATCGGAAAACAATGCGGCTAGCAATCATTTAAGTGCCAATGGTGGTAATGGGTCAAAGACAGGAGAACACAGCGATGAGGAAAGTGATGCTCAGAGTTCTGGTAGCAAAAGGGAGGTTGAAATTCAAAGTGCTGAAAAGTTACCAGAGGTTGTTGCAGATGGTGGGGCAGGCTCATCCAGAGAACATAAAATACAGAATGGTTTCATTGATGGAATGAACACAAAATCACATGCATTAAAGGGTAATGATGATGCTCCAAGTGGAAACGCGTGTGGTGATAGTGAACTACAAGTGCTTTCAACTGAGAAGAATGTGCGTTCCAAATTCCTTAATGGTATTACTTCTGCGAAGGTTGCTGGACAGATAATGGATAATGCCTTGAGGTTTGCTGATTCAAGTTCACTTCGTTCAAGTGATCCTGGAAAAGATCTTTTGGTTGTTGCCCAGACCACAGCTGACAGAAAATGCAAATCTTCAGCACTGGAAAATAATGCTGTCATGGAAAATAACCTTAGTGAAAATTCAAAGGGGACTGCAACAGGCCATGCTGAATCTTGTCCGTCTCACTTTGTGGAGATAAATTTAGAAAAGCAACATCATCTCAATGGTTATACAAACCACAAGTTGAATGAGAAAGATATCTTTAATCACTCAAATTCCTCTGCCTTTTCGAGGTATGGTAATAAGAGGATAGAATCATCAGCTCAACGGCCATTTCCCCCTTCCTTCCGCGTGGTTCACCAACAACCTGTTTACGACAAGAATCCCCAATCCAGCCGGGTCTTGCTTTCCTGTGAACATAATACACGCGAGAGTACAGTGCAAGCTCAGGTCCCTTTGGACAGAAGCACAGAGGGTGCTGCAATCCTGTGTTCCAGTAGTGTAAGAGAAGATGCTGGTACAAGCAGTTCCTCTCCTAGAAAGGACAGTTTAACTCATCCTTCTTATGGGTTTATACCTGTTCCAATTCCTGTTGGTGCTGCTATACCCTACCACTACGGTGCAATTATGCAGCCAATGTATTATCCACAGGGTGCTTTTATGCATTGTGATTCAGCTGCCATCAACAAGACAGCAATTCAGCATGTCTCTTGTCAATCTAACTACCATGAAAATCTTGGTAAACCGCCACAGATTGATGAGCACAAGCAGCCAGAAGAAAACCATCAGTTGCATCATTCAAGACAAATTCTCCGAGAATCAGGAGAACCAGTTGACTTGGCGAAGGCTCATATGGAGCGTATTAACCAGAGTGCTAGCTGTAGCCAGGATATTCGCAAAGGAAGTGGATGCACTGGGAGCGGTGAAACTGATGCAAATACAAATACGGTAATTGCACTAGAAAGTGGCAATGAGAGCGGTGTCCAGAACTGCAGTAATAATGTATTGGATGGTGACCGGTCTCGCCGTGAAGCTGCCTTGTTGAAATTCCGGATGAAAAGAAAAGATAGATGTTTCGAGAAAAAGGTCAGGTATCATAGCAGGAAGAAGCTTGCAGAACAAAGACCCAGGGTTAAGGGCCAGTTTGTGAGCCAAAAACTGAAGTCAGCTATAACAACAGAGGCAGAAACTGACTAG

>OsPRR73
ATGGGTAGCGCCTGCGAAGCTGGTACGGACGAGCCTTCCCGAGACGATGTTAAGGGGACAGGGAATGGCATCCTGGAGAATGGTCATAGTCACAAGCCAGAGGAGGAGGAATGGAGGAATGGCATGGGAGAGGACTTACCCAATGGGCACAGTACACCACCAGAGCCCCAGCAAACAGATGAACAGAAGGAGCACCAAGTGCAGATTGTCCGGTGGGAGAGGTTCCTCCCTGTGAAGACACTGAGGGTCTTGCTGGTGGAGAATGATGACTCTACCCGTCAGGTGGTCAGCGCACTGCTTCGTAAGTGTTGTTATGAAGTTATCCCTGCTGAAAATGGGCTACATGCATGGCAATGTCTTGAAGATCTGCAAAACCACATTGACCTTGTATTGACCGAGGTCGTAATGCCACGTCTGTCTGGCATTGGTCTGCTTAGTAAGATCACAAGCCACAAAATTTGCAAGGATATTCCCGTGATTATGATGTCTTCGAATGACTCAATGGGTACAGTCTTTAAGTGTTTGTCAAAAGGAGCAGTTGACTTTCTAGTGAAGCCTATACGTAAGAATGAACTTAAGAACCTTTGGCAGCATGTTTGGAGACGATGCCACAGTTCCAGTGGCAGTGGAAGCGAAAGTGGCATCCGAACACAAAAGTGTACCAAACCAAAGGTTGATGATGAATATGAGAATAACAGCGGTAGCAATAATGACAACGAGGATGATGATGACAATGATGAAGATGATGACGACTTAAGTGTTGGACACAACGCTAGGGATGGCAGTGATAATGGCAGTGGCACTCAAAGTTCATGGACAAAGCGTGCAGTGGAGATTGACAGCCCACAACAAATGTCTCCTGATCAACCATCCGATCTACCAGATAGTACTTGTGCGCAAGTAATTCACCCCACATCAGAGATATGCAGCAACAGGTGGTTACCGACTGCAAATAAAAGGAGCGGAAAGAAACATAAAGAAAATAACGATGACTCCATGGGGAAGTACTTAGAAATAGGAGCTCCTAGAAATTCTAGTATGGAGTACCAATCTTCTCCAAGAGAGATGTCCGTTAATCCAACAGAAAAACAGCATGAAACTCTCATGCCCCAAAGTAAAACAACAAGAGAAACAGATAGTAGGAACACACAGAATGAACCAACTACTCAAACTGTTGATTTAATTAGTTCAATAGCCAGAAGCACAGATGACAAACAAGTAGTTAGAATCAATAATGCTCCTGATTGCTCCTCCAAGGTTCCAGATGGAAATGATAAAAATCGTGATTCTCTCATTGATATGACATCTGAAGAGTTGGGTTTGAAGAGATTGAAAACAACTGGATCTGCAACTGAAATCCATGATGAACGAAATATTCTGAAAAGATCAGATCTCTCAGCTTTCACCAGGTACCATACAACTGTGGCTTCTAATCAAGGTGGAGCTGGATTTGGGGGAAGCTGTTCACCTCAAGATAACAGTTCAGAGGCTCTGAAAACAGACTCCAACTGCAAGGTGAAGTCAAATTCAGATGCTGCTGAAATAAAGCAAGGCTCCAATGGTAGTAGCAACAACAATGACATGGGCTCCAGTACTAAGAATGCCATCACAAAACCTTCTTCAAACAGGGGAAAAGTGATATCACCATCAGCTGTCAAAGCTACCCAACATACATCAGCATTCCATCCTGTGCAGCGTCAAACGTCACCTGCTAATGTTGTAGGGAAAGACAAAGTTGATGAAGGAATTGCTAATGGAGTTAATGTGGGCCACCCTGTAGATGTACAAAATAGCTTTATGCAGCACCATCATCATGTTCATTACTACGTCCATGTTATGACACAGCAGCAGCAGCAGCCATCCATTGAGCGAGGATCATCAGATGCTCAGTGTGGTTCATCCAATGTATTTGATCCTCCCATTGAAGGTCATGCGGCAAACTATAGTGTGAACGGGAGCTTTTCAGGTGGCCATAATGGAAACAATGGGCAAAGAGGACCTAGTACTGCTCCCAATGTTGGGAGGCCAAACATGGAGACTGTTAATGGTATCGTGGATGAAAATGGGGCTGGAGGTGGCAATGGAAGTGGGAGCGGTAGTGGTAATGACTTGTATCAGAATGGGGTCTGTTACCGAGAAGCTGCATTGAACAAATTCAGACAGAAACGGAAAGTGAGGAACTTTGGAAAAAAGGTGCGCTATCAGAGCAGAAAGAGGTTGGCTGAGCAGCGCCCTCGGATCCGCGGGCAATTCGTGCGACAATCTGGACAGGAAGATCAGGCAGGCCAAGACGAAGACAGATAA

>OsPRR95
ATGGGGGGTGGAGTGGAGGAGAGGAAGGTGGTGGACTTGGAGGACGGGGACGGGGAGGAAGGGGAGGATGCGGCGGCGGTGGCGGCGGGGTCGAGCAGGGAGACGCGGATGCTGCCGAGGATGCCGGTGCGGGTGCTGCTCGCTGAGGGCGACGACTCCACGCGCCACATCATCTGCGCGCTGCTCCGCAAGTGCGGGTATCGAGTTGCCGCAGCCTCTGATGGTGTGAAAGCGTGGGACATACTAAAGGAAAAATCTTTCAATATAGACCTTGTTTTAACTGAAGTTGAACTTCCCTTGATGTCTGGGTTCCTGCTGCTCTCCACGATCATGGAACATGACGCATGCAAGAATATCCCCGTCATAATGATGTCTTCAAATGATTCCGTTAGCATGGTTTTCAAATGCATGCTAAAGGGTGCTGCAGATTTCCTTGTTAAGCCAATACGGAAGAATGAATTAAGGAACTTATGGCAGCATGTTTGGAGAAAACAACTGTCCAGCGGTGTTCTTGATGTGCAGCATACTCAACAAGAAGATAATCTTACAGAAAGGCATGAGCAGAAGACCGGAGTGACGAAAGCTGAACATGTGACTGAAAATGTTGTCCACAAGAACATGGAATGCAGTGAACAAGAAAGTGATGCTCAAAGTTCATGTACAAGGTCAGAACTGGAGGCTGACAGTAGGCAAACCAACAATTTACTGGAATATAAGCAGCCAATGGGAAGGCATTTCTCCAAGCCTGACCACAAGAACACCGAGAAAAATGGAGGGACTAAAATTCATGCATCTAATGATGGCAACTTGATTCCACGGAGGGAAGAGGATGCATCACTGAGGAGGATGACATGTTCAAATGATATTAATTGTGAGAAAGCTTCCAGGGATATGGAGCTGGTTCATATTATTGACAATCAGCAGAAGAATAACACACATATGGAGATGGATGTTGCGAGAGCAAATTCCCGTGGAAATGATGACAAGTGCTTTTCCATTCCAGCACACCAGTTAGAACTTTCTCTCAGAAGATCTGACTACAGTAGATTAGAGAGCCAGGAGAAAAATGAACGAAGAACACTGAACCACTCAACTTCATCTCCATTTTCCTTGTACAACTGCAGGACAGCATCATCTACCATTAATGCTGGTGATGCTCAGGCATGCAGCACCTCGGCAACACATATAGATCTTGAAAACAAAAATGGAGATTCCAAAACTCCCTCTCAAGACAAGAGGGAAACAAACCAACCTCCCATTAGAGTTGTACCATTTCCTGTTCCTGTTGGAGGACTAACATTTGATGGGCAGCCATTCTGGAACGGTGCACCTGTGGCATCCCTTTTCTATCCACAATCAGCTCCTCCCATTTGGAATAGCAAAACATCTACCTGGCAAGACGCAACCACACAAGCAATTTCTCTGCAGCAGAATGGTCCAAAAGATACGGATACTAAGCAGGTTGAAAATGTAGAGGAACAAACTGCTCGGAGTCATCTAAGTGCTAATCGGAAACATCTCCGCATTGAAATTCCTACAGATGAGCCGCGGCATGTTTCTCCAACCACCGGTGAAAGTGGGAGCAGCACTGTGCTGGACAGCGCCAGAAAAACGCTCAGTGGCAGTGTCTGTGACAGCTCTTCCAACCACATGATTGCCCCTACTGAATCATCAAATGTTGTTCCTGAAAATCCTGATGGTTTACGCCACCTGAGCCAACGAGAGGCTGCACTGAACAAGTTCCGGCTTAAGAGGAAAGACAGGTGCTTTGAGAAGAAGGTGCGATACCAGAGCAGGAAGCTACTAGCCGAGCAGCGACCGCGGGTTAAGGGTCAGTTTGTTCGGCAAGATCATGGTGTCCAAGGGAGCTAG


Sorghum bicolor
>SbPRR1/TOC1
ATGGTAGGCGGCGGCGAGGGGGATCGAGTGGGCGGCGGCGGCGGAGGCGGAGGCGGTTTGGGGGTTGGGGGAGGCCAGCAGTTCGTGGACCGGAGCAAGGTGAGGATTCTTCTCTGCGACGGTGATGCCACCAGCTCCAGGGAGGTGCTGCGGCTCCTCTGCAACTGCTCGTACCATGTAACTTGCGCCAAGTCTCCGCGGCAGGTGATCAATATTCTCAACTATGAGGGTGGCGAGATCGACATCATCCTGGCTGAGGTTGATCTGCCGGTCTCCAAGTGCTTCAAGATGCTCAAGTACATTGCCAGGAACAAGGACCTGCGCCACATCCCAATCATCATGATGTCCAACAGAGATGAGGTGTCTGTTGTTGTCAAGTGCTTGCGGCTTGGTGCGGCTGAGTACCTGGTGAAGCCGCTGCGCACGAATGAGCTGCTGAACCTTTGGACCCATGTGTGGCGGCGGAGACGGATGCTTGGTTTGCCCGAGAAAAACTTCTTCCATGACAACTTCGAGTTGGTGCTCTCAGAACCTAGTGATGCCAACACCAATAGCACCACCCTCCTCTCAGACGAGACAGACGATAGGCCTAAAGAAAACATGAATCAAGAAACGGGCACCTCAAATCAACTTGAATACGAGTCCAATCCTTCTGTTGCTGAGCCTGACCAGAGAGACAAGATGGAGGGTGTACCAGGTTCTGTCGTAGATGCCAGTCAAGCATCCTCTCCGGGAAGAATGTTTTCACGCCCTATAAAAACTAACCTGAGGATTGCTGAGTCGTCTGCATTTCTAGCATATGTTAAGACAAGCACACCAACCACCAGCTCACTTGATAGCGAATTACAAAGAGGTGGCAGTCAGTTAGATTCTTTGGATAACCAGGGTAATTGCTCTAGTGCAACAGATAGAAGTGACACCGTTACTGATGTAAATATTCGGAATAAAGAAGCTTTTGAGATGCCTGTGCAGTACCCTATGGTGTGCTTTTCTTCCTCTAACACACATATGGAGCGAAGCAATGAAGGCCATAATGATACTTCAGGAACTCCACCTGTATACCATTTCCCATTTTATTATCCAGGGATGGTAGAGCACAACATGGCAGTTTCATCAGTGCAAAATTTTCAAGCAAACATAAACAATGCTCAAGCACATACACCACCAACGATGCTTCCCCAGTACAATGTTTATCCCCAATGCCATGGTTTACCTATGATATCATCATTTCAGTTTAATCCTGCGGGCATGAATATGCACTCAAGTCATTTACCGACACAAAATGTGTGGTCATCGGCATCAAGCACACCAATGCCTGAGGAAACATGCAATCGCTCTGAAAGGAGGGCTGCAGCACTTGCCAAATTCAGGCAGAAAAGGAAAGAGCGCTGCTTTGACAAGAAGGTGAGGTATGTGAACCGGAAGAAACTTGCTGAAACAAGGCCTAGGGTGCGAGGTCAATTTGTTAGACAGGCAAGCAATACAGATATCACCAGCACTGGAGAtgatatctctgaagacgaagatgacgatccatcgtccagggaggtagatatcgtttcCTCTCCAGAGTAG

>SbPRR37
ATGATGCTTCGGAATAACAACAATAATCTGAGGAGCAATGGCCCATCAGATGGCTTGCTCAGCAGGCCAACCCCTGCAGTACTCCAGGATGATGACGATGGTGGTGATGATGATACGGAAAACCAGCAGCAGGAGGCGGTCTACTGGGAGCGCTTCCTCCAGAAGAAGACCATCAACGTCTTGCTCGTGGAGAGTGACGACTGCACTAGGCGGGTCGTCAGTGCCCTTCTTCGTCACTGCATGTACCAAGTTATCTCTGCTGAAAATGGCCAGCAAGCATGGAATTATCTTGAAGATAAGCAGAACAACATAGATATTGTTTTGATTGAGGTTTTTATGCCCGGTGTGTCTGGAATTTCTCTGCTGAGTAGGATCATGAGCCACAATATTTGCAAGAATATTCCAGTGATTATGATGTCTTCGAATGATGCTAGGAATACAGTCTTTAAATGTTTGTCGAAAGGTGCTGTTGACTTTTTAGTCAATCCTATACGTAAGAATGAACTTAAGAATCTTTGGCAGCATGTATGGAGACGGTGTCACAGCTCAAGTGGTAGTGGAAGTGAAAGTGGCATTCAGACGCAGAAGTGTGGCAAATCAAAAGGTGGAAAAGAATCTGGTAATAATAGTGGTAGCAATGACAGTCACGACAACGAAGCAGACATGGGACTTAATGCAAGGGATGACAGTGATAATGGCAGTGGCACTCAAGCGCAGAGCTCATGGACTAAGTGTGCTGTGGAGATGGACAGCCCACAGGCAATGTCTCTGGATCAGTTAGCCGATTCACCTGATAGCACTTGTGCACGGCTACCAGGTACGAGCAACAGAAACTGCATGAAGCAAAAATACACTAATGACGACTTCAAGGAAAAGGACTTGGAGATAGGTGGCCCTGGAAATTTATATATAGATCACCAATCTTCCCCAAATGAGAGGCCTATCAAAGCAACAGATGGACGTTGTGAGTACCCACCAAAAAACAATTCGAAGGAGTCAATGATGCAAAATCTAGAGGACCCAACTGTTCGAGCTGCTGATCTAATTGGTTCAATGGCCAAAAACATGGATACCCAGGAGGCAGCGAGAGCTGCAGATACCCCTAATCTCCCTTCCAAAGTGCCAGAAGGGAAAGATAAGAACAAGCATGACAAAATTTTGCCATCACTTGAGTTGAGTTTGAAGAGGTCGAGATCATGTGGATATGGTGCCAATACAGTCAAAGCTGATGAACAACAGAATGTATTAAGACAGTCAAATCTCTCAGCTTTTACAAGGTACCATACATCTACGGCTTCCAATCAAGGTGGGACTGGATTAGTAGGGAGCTGTTCGCCACATGACAACAGCTCAGAGGCTATGAAAACAGATTCTACTTACAACATGAAGTCAAATTCAGATGCTGCTCCAATAAAACAAGGCTCCAACGGAAGTAGCAATAACAATGACATGGGTTCCACTACAAAGAATGTTGTGACAAAGCCCACTACAAATAATAAGGACAGGGTAATGTTGCCCTCATCAGCTATTAATAAGGCTAATGGACACACATCAGCATTCCACCCTGTGCAGCATTGGACGATGGTTCCAGCTAATGCAGCAGGAGGGACAGCGAAGGCTGATGAAGTGGCCAACATTGCAGGTTACCCTTCAGGTGACATGCAGTGTAACCTGATGCAATGGTACCCTCGTCCAACCCTTCATTACGTCCAGTTTGATGGTGCACGGGAGAATGGTGGATCGGGAGCCCTGGAATGTGGTTCCTCCAACGTATTTGATCCTCCAGTTGAAGGTCAAGCTACTAACTATGGTGTGAACAGGAGCAACTCAGGCAGTAACAATGCAACCAAGGGGCAGAATGGAAGTAATACAGTTGGTGCAAGCATGGCTGGTCCAAATGCAAATGCAAATGGTAATGCTGGACGAACAAACATGGAGATTGCTAATGAGGTCATCGACAAAAGTGGACATGCAGGAGGTGGCAATGGGAGTGGCAGTGGCAGTGGCAATGACACATATGTCAAACGGCTTGCAGCGGGCTTGACACCACGACAAGCACAACTAAAGAAATATAGAGAGAAAAAGAAAGATCGAAACTTTGGGAAAAAGGTGCGGTACCAGAGCAGAAAGAGGCTGGCCGACCAGCGGCCGCGGTTTCGTGGGCAGTTCGTGAAGCAAGCCTTGCAAGATCAGGGCGAACAGGACGGAACTGGAGAGAGATGA

>SbPRR59
ATGTCTCCCGACGCCGACGCCACCGCCGGAGCCGGAGGCGGCGACGGCGGCGGAGGAGAGGCGGGCGGCGGCGCCGGCGCCGGCGCGTCGTCGTCTCCGGCGTCCGCGGCGGCGAACGGGCGGGCGCTGGTGCGGTGGGACCAGATCCTGCCGCGCCGCTCGCTGCGGGTGCTCCTCGTCGAGCACGACGACTCCACGCGCCAGGTCGTCACCGCGCTGCTCCGCAAGTGCGGCTACCGCGTGGCGGCGGTCGCGGACGGGATGAAGGCGTGGGAGGTGATGCGGGGCCGCGCATACGCCTTCGACCTCGTCCTCACCGAGGTCGCCATGCCCTCGCTCTCCGGCATCCAGCTGCTCTCCAGGATCGTCGCCGCCGACGAGTGCAAGAACATCCCCGTCATCATGATGTCGTCCCAAGATTCTATTGGCACGGTGCTCAAGTGCATGCAGAAAGGTGCGGTGGACTTCCTTGTGAAACCGGTGAGAAAGAACGAGCTGCGTAACTTATGGCAACATGTATGGAGGCGCCATGCAATGAATTGCCAGACAAATGGATCAGAAAATAATGCAGCCAGCAATCATATAAGTGCAAATGTTGCTAATGGGTCAAAGACAGGAGAAAACAGCGATGAGGAAAGTGATGCACAAAGCTTTGGTAGCAAGAGGGAGACCGAAATTCAGAGTGTTGAGAAGTTACCAGACATTCGTAGAGATGAAGATGAAGTGGCTGGCTCATCCAAAAAAACTGAATCACAAAATAAGTCTTATGATGTCAGAGTAAACACAAAAGTGGATGCGTCAAAAGATAGCGATGGTGCTCCAAGTGGAAGTGAGAAGAATGTACACTCCAAATGCCTTAATGGTATTACTTCTGCAAAAGTGGCTGAGCAAATTATGGATAATGCCTTGAGGATCACGGATGCAAGTTCACGTCGTCCAAGCAATCTTGGCAAAGATTTGGCTATGACTGAGCCAGAAGCTGACAGAAAATGCCAATCTTCAGTCATGGAAAATAATGCTGTCACAGAAAATAACCTTGGTGTAAAGTCAAAGGGTGCTGCAATATGTCCTGCTGATTCTTGCCCCTCCCAGTTTCTGGAGACCAACTTGGGAAAGCAACACCATCTTAATGGTTACAAAAACCAAGAATTCAGGGAAAAAGATATCTTTAATCACTCGAATTCCTCTGCCTTTTCAAGATATGGCAATAAAAGGATAGAACCATCAGGGGAAATACAATTTTTTCCTTCTCTCTGCATCACTGGGCAAGAGCATGTTCATGGCAAGGACCCAGTTTTCCAACCCAATGGGGTGTTGCTGCCTCCCAATGAACATAATACAGGTGAGAGTACAAGGCAAACTCGAATTACTTTGGACAGTAGCCCGGAGGGTGCTGCCATCATGTGTTCCAGTAGTGCTAGGGAAGATGCTGGTGTAAGTAGTTCTTCCCATAGAAAGGACAGCATGAGCCATCCGTCATATGGGTTTATTCCTGTTCCAATTCCTGTCGGTCCTGGGATGCCCTACCATTATGGTGCAATTCTGCAGCCAGTATACTACCCACAGGGTCCTCTTATGCACTGTGATTCAGCTGGAATCAACAAGGCAGCAATCCAGCATGGCTCTGGTCAATCTAATTACCATGAAGCTCCTGGTAAACCATCTCAGGATGATGAGCACAAACAGTCAGAGGAAAACCACCAGTTGCATCATTCAAGACAAATTCTTCGGGAATCAGGAGAACCAATTGAGATGGCGAGAGCTCACATGGATCGCGCTAACCAGAGTGCAAGTTGCAGCCAAGATATCTGCAAAGGAAGTGGATGCACGGGTAGTGGTGAAGCCGATATCAATGCAAATACAATGGTTGCCCTTGAGAGTGGCAACGAAAGTGGCATCCAAAATGGTGATAGGTCTCGCCGTGAAGCCGCGTTGATGAAGTTCCGCATGAAAAGGAAAGATAGATGCTTCGAGAAAAAGGTTAGGTATCATAGCAGGAAGAAGCTTGCAGAGCAAAGGCCAAGAGTTAAGGGACAATTTGTAAGCCAAAAGCAGAAATCTGCTACAACAACAGAAGCAGAAACTGACTTGTGA

>SbPRR73
ATGGGTAGCGCTTGCCAAGCTGGCATGGACGGGCCTTCCCGCAAGGATGTGTTGGGGATAGGGAATGTCGCCTTAGAGAATGGCCACCATGAGGTTGGAGCTGATGCAGATGAATGGAGGGAAAAGGAAGAGGACTTGGCCAATGGGCACAGTGCGCCACCGGGCATGCAGCAGGTGGATGAGCAGGAGCAACAAGGACAAAGCATTCACTGGGAGAGGTTCCTACCTGTGAAGACACTGAGAGTCATGCTGGTGGAGAATGATGACTCTACTCGTCAGGTGGTCAGTGCCCTGCTCCGTAAGTGCTGCTATGAAGTTATCCCTGCTGAAAATGGTTCACATGCATGGCGATATCTTGAAGATCTGCAGAACAACATTGACCTTGTATTGACTGAGGTTTTCATGCCTTGTCTATCTGGCATCGGTCTGCTTAGCAAAATCACAAGTCACAAAATTTGCAAGGACATTCCTGTGATTATGATGTCTTCAAATGACTCTATGAGTATGGTGTTTAAGTGTTTGTCGAAGGGAGCAGTTGACTTCTTGGTAAAGCCACTACGTAAGAATGAGCTTAAGAACCTTTGGCAGCACGTTTGGAGGCGATGCCACAGTTCCAGTGGCAGTGGAAGTGAAAGCGGCATCCAGACACAGAAGTGTGCCAAACCAAATACTGGTGATGAGTATGAGAACGACAGTGACAGCAATCATGATGATGAAGAAAATGATGAAGACGACGACGATGACTTCAGTGTCGGACTCAATGCTAGGGATGGAAGTGATAATGGCAGTGGTACTCAAAGCTCATGGACAAAACGTGCTGTGGAGATTGACAGTCCAGAACCTATGTCTCCTGATCAACTAGCAGATCCACCTGATAGTACATGTGCACAAGTAATTCACCCCAAATCAGAGATATGCAGTAACAAGTGGCTACCGACAGCAAACAAAAGGAATGGCAAGAAACATAAGGAGAATAAAGATGAATCTATGGGAAGATACTTAGAAATAGGTGCTCCTAGGAACTCAAGTGCAGAATATCAATCATCTCTCAATGACGTATCTGTTAATCCAACAGAAAAACGTCATGAGACTCACATGCCCCAATGCAAATCCAAAAAGAAAATGATGGCAGAAGATGATTGTACAGACATACCTAGTGAAATAAATACTGAAACTGCTGATTTGATTAGCTCAATAGCCAGAAACACAGAAGGCCAACAAGCAGTACGAGCTGTTGATGCACCTGATGGCCCTTCCAAGATGCCCGATGGAAATGATAAGAATCATGATTCTCATATCGTGGTGACACCCCATGAGTTGGGTTTGAAGAGATTGAGAACAGATGGAGCTGCAGATGAAATCCATGATGAGCGAAATATTCTCAAAAGATCAGATCAGTCAGCCTTCACCAGGTACCATACATCTGTGGCTTCCAATCAAGGTGGAGCAAGATGTGGGGAAAGCTCTTCACCACAAGATAACAGTTCTGAGGCTGTGAAAACAGACTCTACATGCAAGATGAAGTCAAATTCAGATGCTGCTCCAATAAAGCAGGGCTCCAATGGCAGTAGCAACAACGATGTGGGCTCCAGTACAAAGAATGTTATTGCAAAGCCTTCAGCTAACAGGGAGAGAGTAACGTCACCATCAGCCATCAAATCTACCCAGCATGCCTCAGCATTTCATACTATACAGAATCAAACATCACCTGCTAATCTGGTTGGTAAAGACAAAGCTGATGAAGGAATTTCCAATGCAGTGAAAATGAGCCACCCAACAGAGGTTCCACAAAGCTGCGTCCAGCATCATCACCACGTGCATTATTACCTCCATGTTATGACACAGAAACAGTCATCAATCGACCGTGGATCATCAGATGTTCAGTGTGGTTCGTCAAATGTGTTTGATCCTCCTGTTGAAGGACATGCTGCAAACTATAGTGTGAATGGGGGTGTCTCAGTTGGTCATAATGGGTGCAATGGCCAGAATGGAACGAGCACTGTCCCCAATATTGCAAGACCAAACATAGAGAGTGTTAATGGTACCGTGAGCCAAAATATCGCTGGAGGTGGCATTGTAAGTGGGAGTGGGAGTGGCAATGATGTGTATCAGAATCGATTCCCCCAACGAGAAGCTGCATTGAACAAATTCAGACTGAAGCGGAAAGATCGGAACTTTGGTAAAAAGGTTCGCTACCAAAGCAGGAAGAGGCTTGCTGAGCAGCGGCCTCGGGTCCGTGGACAGTTTGTGCGACAATCTGGGCAAGAAGATCAAGCAGCACAAGGTTCAGAAAGATGA

>SbPRR95
ATGGGGGGAGGAGTAGATGAGGTGGTGAAGGTGGTGGTGGATTTGGAGGACGGTGAGGGCGAGGAGGACGCAGAGGCCGCGGCGGCGGAGGGGTCGAGCAGGGAGACGCGGATGCTGCCAAGGATGCCGGTGCGTGTGCTGCTCGCCGAGGGCGACGACTCCACGCGCCACGTCATCTCCGCGCTGCTCCGCAAGTGCGGCTACCGAGTTGCTGCAGCCTCTGATGGTGTGAAGGCGTGGGACATATTAAAGGAAAAATCTTTCAACATTGACCTTGTTTTAACTGAAGTTGATCTGCCTTTGATGTCTGGGTTCCTCTTGTTATCCACAATCATGGAGCATGATGCGTCCAAGAACATCCCTGTTATAATGATGTCTTCCCACGACTCAGTTAGCATGGTTTTCAAATGCATGCTAAAGGGCGCAGCAGATTTTCTTGTTAAGCCAATAAGAAAGAATGAATTAAGGAACTTGTGGCAGCACGTTTGGAGAAAACAACTGGCAAACGGTGGGTCTGATGTGCACCACATACAACAAGAAGAGAATCTTGCAGAAAGAATCGAACAGAAGACTGGCGTGACAAAAGCTGATAATTTGAACAGAGATGGGCCCCGTAAAAATAGAGAATGCAGTGAACAAGAAAGTGATGCTCAAAGTTCTTGCACGCGGTCAGAGCTGGAGGCTGAAAGTAAGCAAACTAACAACATTTTGGAGTATAAGCAATCTACTGAAAGGCATTTGTCTATTCCTAGCCACAAGAATGTTGAGCTAAATGGACAGACCAAAATACGAAGTAAGGATAATAACTTGATTCCAACAAGAGAAGATGATTTATCACCAAAGAAAAGAACATGTTTGAATGACAATAATTCTGAGAGAGCTTCCAAAGATATGGACCTAGTCCACATTATGGATAATCAGCAGAAGCATAACACACAGAGGGAGGTGGATACTATGAGAACAACATCTAGGGGAAATGATGAGAAGGGCTCCATCCCAGCACACCAGTTGGAACTTTCTCTTAGAAGAACTGACTACGGAAAATTGGAGAACCATGATAAAAATGATAGAAGAACACTGAACCATTCAACTTCATCTGCATTTTCCTTGTATAATTGCAGGGCTGTGTCCACCTCAGGAAATGCTGGTGATGGTCAATTATGCAGCACCTCAGAAACACTAATGGATGTTGAAAATAAAAATGGAGATTCGGCAGCTCCCTCTCAAGACATGACTGAAACAAATCGTCCTCCTATTAGAGTTGTACCATTTCCTGTCCCTGTTCAAGGTCTCACATTTGATGGGCAGCCATTCTGGAATGGTACACCGATGGCATCCCTATTCTACCCACAGTCAGCTCCTCCCATTTGGAATAGCAAAACATCCATGTGGCAAGAATCAACTCAAGCAACTTCACTGCCACAAAAATCGCAACAGAATGAGCCAAATGAAATGGGTGCTAAACCAGTTGAAAATGCAGAGGAACAATTTGTCACAGGTCCTCCGAGTGCCAGTGGGAAGCAGCTGTGTGTTGAAGTTCCTAAAGATGATCCACGGCATATTTCTCCTATGACTGGTGAAAGTGGAATTAGTACCATGCTAGACAGCACTAGAAATACTCTGAGCAGCAGTGGCTGTGATAGCACTTCCAACCAGTTCACTGCCCCTACTGAATCATCCAATGTATATAAGGGCGTTCCTGAAACCCCAAGCACCGAAGGTTCACGACACCTGAGCCAGCGAGAGGCTGCACTGAACAAATTCCGGCTAAAGAGGAAGGACAGGTGCTTTGAGAAGAAGGTTCGGTACCAGAGCCGGAAATTACTTGCAGAGCAGCGTCCACGGGTCAAGGGCCAGTTTGTTCGTCAAGATCATAGCATCCAAGGAAGCTAG
