# Supplementary material for: Phylogenetic footprint of the plant clock system in angiosperms: evolutionary processes of Pseudo-Response Regulators
Source: BMC Evol Biol. 2010 May 1;10:126. doi: 10.1186/1471-2148-10-126 (PMC2887406; doi:10.1186/1471-2148-10-126)
Supplement: Additional file 3 — Alignments of the amino acid sequences encoded by angiosperm PRR genes. Amino acid sequences were aligned using TCoffee program http://www.ebi.ac.uk/t-coffee/. Amino acid conservation was highlighted by the boxshade program http://www.ch.embnet.org/software/BOX_form.html. Identical and similar amino acid residues are highlighted with black and gray shading, respectively. Blue boxes indicate the regions that were represented in Figures 4 and 5. [file 1471-2148-10-126-S3.PDF]

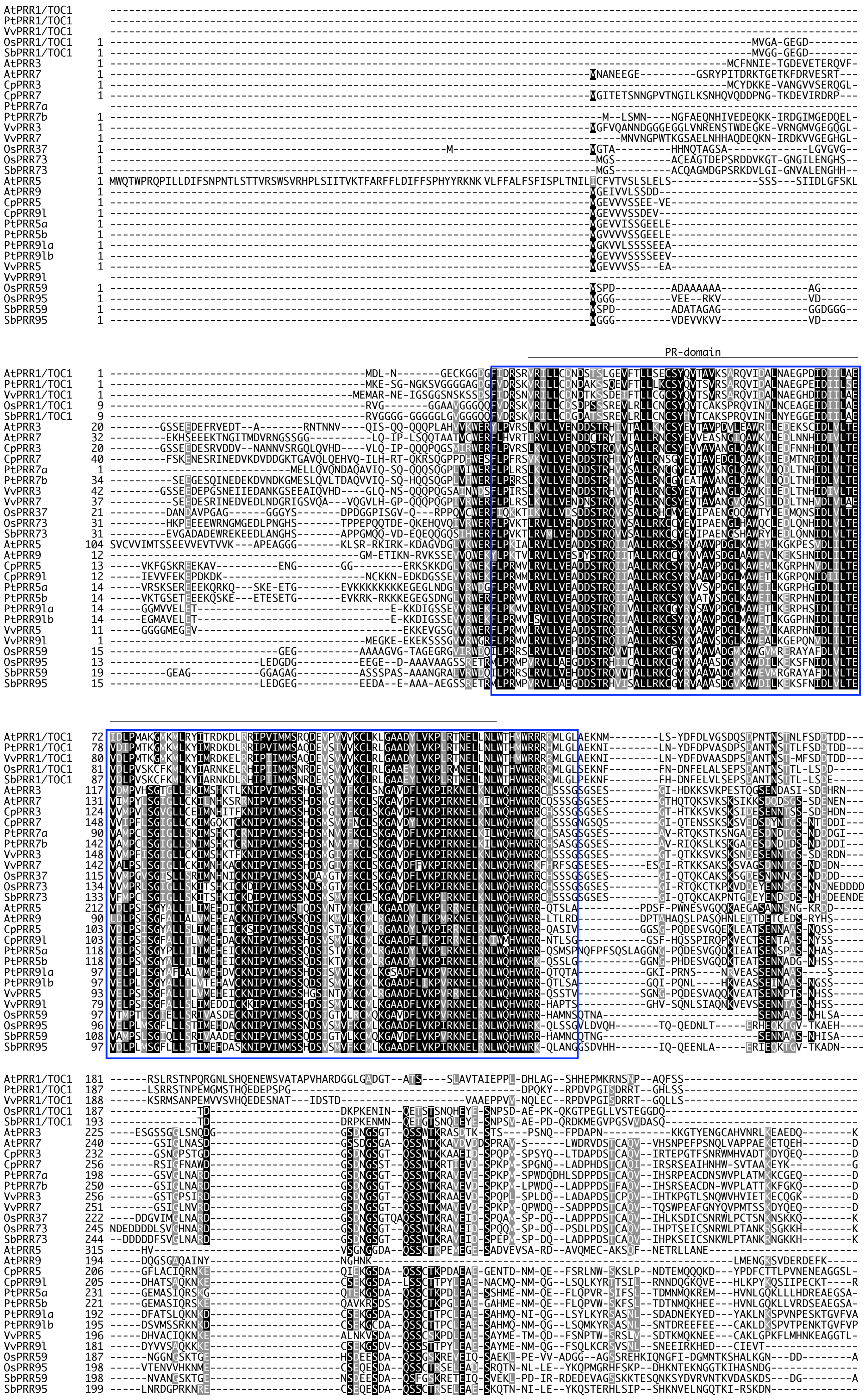
PR-domain
AtPRR1/TOC1
PtPRR1/TOC1
VvPRR1/TOC1
OsPRR1/TOC1
SbPRR1/TOC1
AtPRR3
AtPRR7
CpPRR3
CpPRR7
PtPRR7a
PtPRR7b
VvPRR3
VvPRR7
OsPRR37
OsPRR73
SbPRR73
AtPRR5
AtPRR9
CpPRR5
CpPRR9l
PtPRR5a
PtPRR5b
PtPRR9la
PtPRR9lb
VvPRR5
VvPRR9l
OsPRR59
OsPRR95
SbPRR59
SbPRR95

```
AtPRR1/TOC1      ------------------------------------------------------------------------------------------------------------------------
PtPRR1/TOC1      ------------------------------------------------------------------------------------------------------------------------
VvPRR1/TOC1      ------------------------------------------------------------------------------------------------------------------------
OsPRR1/TOC1  235 ---------------------------------------------------------------------ASSPGVMFSRPI---------------------------------------
SbPRR1/TOC1  241 ---------------------------------------------------------------------ASSPGRMFSRPI---------------------------------------
AtPRR3       293 EQIGTGS--QT-GM-SMSK----------------------------------------------------KAEEPGD---------LEKNAKYSVQALERNNDDTLNRSSGNSQVESK-
AtPRR7       313 DKFEDVTMGRDLEI-SIRRNCDLALEPK-DEPLSKTTGIMRQD-NSFEKSSS----KWKMKVGKGPLDLSSESPSSKQ---MHE-DGGSSFKAMSSHLQDNREPEAPNTHLKTLDTNEA-
CpPRR3       308 EQLDNAEIGKD-GV-RVLKIPDFECEDQ-CENLSTPKSKKRQT-KFPEGDNK----LLDGEKLE--HSNDNSTGKPRD---RAL-SVLNQITKSAIPEYESKDIDTPNAASGILQIKDK-
CpPRR7       329 DKFSNMLMGKDLEI-GVTRIPKLQLKKPSAEALSHIDGLPGLN-SIKDPENL----LKE-------EQGNNSDSDLKS---LSA-DLIGSINYKC-PNLECVTFGTTNLLPKVSDDKNK-
PtPRR7a      276 DELDNIVMGKDLEI-GVPRIPNLQLKDP-IKRVPTNIADNDGE-KFPEIKSKHDGGHLEKRQQE--LN------------------------------------NVPNGLSS--NRKNE-
PtPRR7b      325 DELDNFVMGKDLEI-GVPRIPNLQHKDL-SKEVLTNIAGNNGE-KFREIKSEQDSGHLEKGQLE--LNSEKHNTELRN---QGN-DLKGVSTNITNPQIESEVVDISNSLSS--NKKNE-
VvPRR3       332 EQPDNVAMGRDLEL-GVATNPAVQLEYQ-HEKFSTYPTCKRQN-KLPESDSK----PFDKEHL----------------------EHNSTSANCTDPQVESRTFENPNGLSDVSQIKDK-
VvPRR7       327 DELDNVEMGKDLKI-GVPRNSNLQLQDD-V-------VGANKD-KFHELTLKKDDEKLENRQMD--LNSNKPNDELDK---EAV-DLMSVIANNTNPQKKSMGFKTPSGLSEVPETKDK-
OsPRR37      302 ETNDD-FKGKDLEI-GSPRNLNTAYQSS-PNERSIKPTDRRNE--YPLQNNSK----------E--AAMENLE-ESSV---RAA-DLIGSMAKNMDAQQAARAANAPNCSSKVPEGKDK-
OsPRR73      327 ENNDD-SMGKYLEI-GAPRNSSMEYQSS-PREMSVNPTEKQHETLMPQSKTTR----------E--TDSRNTQNEPTT---QTV-DLISSIARSTDDKQVVRINNAPDCSSKVPDGNDK-
SbPRR73      324 ENKDE-SMGRYLEI-GAPRNSSAEYQSS-LNDVSVNPTEKRHETHMPQCKSKK------KMMAE--DDCTDIPSEINT---ETA-DLISSIARNTEGQQAVRAVDAPDGPSKMPDGNDK-
AtPRR5       367 ------------------------------------------------------------------------LQSKQA---EAI-DFMGASFRR----------------------TGRR
AtPRR9       225 ----------------------------------------------------------ETFDVT--MDLIGGIDKRP----------------D-----------------------SIY
CpPRR5       286 -------------------------AGA---CEEVIKASANE-DVGPES----QGRDANSKHEA--CN---GFASSSK---EAI-DFMGQFSTH----------------------KSSS
CpPRR9l      283 ENFDRLGTKVAPCV-QAYRSSALKLEEG---RDCCKGTGQEE-GVCTEN----DDRDADISVET--HGCNDEPVEPSN---GAV-DLIGTFDDHQ----L-----------QRMYRCSNF
PtPRR5a      311 -------------------------AAA---REDANIMDVDK-EISPGN----GRTGAYVAIES--CDNDVALANSHR---EAF-DFMGASTNR----------------------SSSF
PtPRR5b      301 -------------------------TAV---CEDSNKITVDK-EITPGS----GRVTANIAIEG--CDKIGALANSPR---EAI-DFMGASTNH-----------------------SSF
PtPRR9la     274 ERSNRTRPDREPYH-GAYNPTASRLVEE---HACAKSAIHDE-NSRPEN----DREHANS---S--FGHDDVLAETSS---GAI-DLIGSFNNQP----------------KHTYAYSSL
PtPRR9lb     277 ERPNRMESDGEPCS-GAYNPTSLRLLEE---HACAKSAIQDE-NSRPEN----DRGLANS---S--FGCDDVPFESSS---GAI-DLIGTLNNGP----------------KTTYVHSSL
VvPRR5       276 -------------------------EAA---CRDVNTMTQPE-AVEPEN----DGQGANAPSEA--CGNNAILGSSSR---EAI-DLIGVFDNSK----------------KCTYGNSSS
VvPRR9l      248 ---------------------------------------SGD-GVGPAS----HIENTDITGEN--HGCNEKWIEPSS---GAI-DLISTFDNYP----------------KDRNQFSSS
OsPRR59      260 PSGNA-CGDSELQVLSTEKNVRSKFLN--------GITSAKVAGQIMDNALR----------------FADSSSLRSSDPGKDLLVVAQTTA----DRKCKSSALENNAVMENNLSENSK
OsPRR95      269 -----------------------------------NLIPRREEDASLRRMTC----------------SNDINCEKAS---RDM-ELVHIID----NQQKNNTHMEMDVARANSRGNDD-
SbPRR59      274 PSG-------------SEKNVHSKCLN--------GITSAKVAEQIMDNALR----------------ITDASSRRPSNLGKDL-AMTEPEA----DRKCQSSVMENNAVTENNLGVKSK
SbPRR95      269 -----------------------------------NLIPTREDDLSPKKRTC----------------LNDNNSERAS---KDM-DLVHIMD----NQQKHNTQREVDTMRTTSRGNDE-

AtPRR1/TOC1  256 -----------------------------------APKKSRLKIGESSAFFTYVK-ST-VLRTNGQDPPLVDGNGSLHLHRGLAEKFQVVASEGINNTKQARRATPKSTVLRTNGQDPPL
PtPRR1/TOC1  232 -----------------------------------GPKKSELKIGESSAFFTYVK-PS-TVKNNSQGVALIEDNTNQNLR--MEEKLQVCGEQMLNDAH---------------------
VvPRR1/TOC1  246 -----------------------------------GPKKSELKVGESSAFFTYVK-SS-MIKTNSQGIPNINESAPQHSR--MEERVQAWSEKGVNDTQ---------------------
OsPRR1/TOC1  247 --------------------------------------KTNLRVAESSAFLAYVK-SS-T------------------------------------------------------------
SbPRR1/TOC1  253 --------------------------------------KTNLRIAESSAFLAYVK-TS-T------------------------------------------------------------
AtPRR3       347 ----APSSNREDLQSLEQTLKKTRE-D---RDYK-VGDRSVLRHSNLSAFSKYNNGAT-SAKKAPEE-----------------------------------------------------
AtPRR7       421 ----S-VKISEELMHVEHSSKRHRG-TKDDGTLV-RDDRNVLRRSEGSAFSRYNP-AS-NANKISGG-----------------------------------------------------
CpPRR3       413 ----A-CSDPGEMPSLELTLTRPRG-TVDDRNAA-NDDRNVLRHSDLSAFSKYNT-AS-SANQGPTG-----------------------------------------------------
CpPRR7       430 ----V-IYDTEEMPCLELSLKRFSD-AGDTRK-S-THDRNLLRHSKLSAFSRYNS-AS-TANQAPTG-----------------------------------------------------
PtPRR7a      352 ----V-TYETKEVPSFELSLKRLRD-IGDAGA-S-SHDRNVLRHSDLSAFSRYNS-AS-TADQAPTG-----------------------------------------------------
PtPRR7b      433 ----V-IYETKEMPSLELVLKRLRD-TGDAWA-S-ANDRNVLRHSDLSAFSRYNS-AS-TAYQAPTG-----------------------------------------------------
VvPRR3       422 ----G-SCETTELPPLDLSLKRLRG-AGDVGACV-HDDRSILRHSDLSAFSKSNT-TS-SVKQAPTG-----------------------------------------------------
VvPRR7       430 ----A-MYDKKEIPSLELSLKRLRD-TGGTDT-N-PHDQIIWRHSDLSAFSRYNS-AS-TAIQASTG-----------------------------------------------------
OsPRR37      399 ----N-RDN--IMPSLELSLKRSRS-TGDGANAIQEEQRNVLRRSDLSAFTRYHT-PV-ASNQGGTG-----------------------------------------------------
OsPRR73      427 ----N-RDSLIDMTSEELGLKRLKT-TGSATEI--HDERNILKRSDLSAFTRYHT-TV-ASNQGGAG-----------------------------------------------------
SbPRR73      428 ----N-HDSHIVVTPHELGLKRLRT-DGAADEI--HDERNILKRSDQSAFTRYHT-SV-ASNQGGAR-----------------------------------------------------
AtPRR5       389 NREES-VAQYESRIELDLSLRR--P-NASENQS--SGDRPSLHPSSASAFTRYVH-RP-LQTQCSA------------------------------------------------------
AtPRR9       246 KDKS---RDECVGPELGLSLKRSCS-VSFENQD--ESKHQKLSLSDASAFSRFEE-SK-SAEK---------------------------------------------------------
CpPRR5       342 NNGM---NK-------------------------------------------YAS-RV-KQPESST------------------------------------------------------
CpPRR9l      373 NDYI---NKSEFAPQLELSLTRFCP-SSSMNKG--YHERHTLNHSNASAFSWYNN-NKLLQSPFSA------------------------------------------------------
PtPRR5a      370 NNVK---INFDSSPHLDLSLRRSHP-SGFEIRD--TEERRALWHSNASAFTQYIN-RP-LQLPHSA------------------------------------------------------
PtPRR5b      359 NNVE---IHFCSSPHLDLSLRRSHP-SGFETQV--TEERHTLRHSNASAFTWYTN-RA-SQLPHSA------------------------------------------------------
PtPRR9la     360 HDAT---NKFEFPPLLELSLRRLYP-SSSKNQG--LDERHALNHSNSSAFSLYNS-KT-LQSLFPT------------------------------------------------------
PtPRR9lb     363 HYGT---NKFEFAPQLELSLKRLYP-SSSKNQG--VDERHALNHSHASAFSWYNS-KT-LQPPFPA------------------------------------------------------
VvPRR5       341 NNGT---KKSDSIPQLDLSLRRSHP-SSPENQV--ADERHTLNHSNGSAFSRYIN-RS-LQPPHLP------------------------------------------------------
VvPRR9l      302 NDGI---SKDGFAPQLELSLRRFQP-CSSKNHG--SDERHTLNHSNSSAFSWYNN-GKSLQPLFPT------------------------------------------------------
OsPRR59      351 GTATG-HAESCPSHFVEINLEKQHHLNGYTNHK--LNEKDIFNHSNSSAFSRYGN-KR-IESSAQRP-----------------------------------------------------
OsPRR95      329 ------KCFSIPAHQLELSLRRSDY-SRLESQE--KNERRTLNHSTSSPFSLYNC-RT-ASSTINAG-----------------------------------------------------
SbPRR59      352 GAAIC-PADSCPSQFLETNLGKQHHLNGYKNQE--FREKDIFNHSNSSAFSRYGN-KR-IEPSGEIQ-----------------------------------------------------
SbPRR95      329 -------KGSIPAHQLELSLRRTDY-GKLENHD--KNDRRTLNHSTSSAFSLYNC-RA-VSTSGNAG-----------------------------------------------------

AtPRR1/TOC1  339 VNGNGSHHLHRGAAE--KFQVVASEGIN-----NT----KQAHRSRGTEQYH--------------------------SQGETLQ-NGASYPHSL--ERSRTLPTSME-----SHG----
PtPRR1/TOC1  292 ----------------------------------------LQENGEALEI-H--------------------------SQVDDFR-SSSSIPDSLSLERSCTPPMSRE-----FPQ----
VvPRR1/TOC1  306 ----------------------------------------EHENGEAWEN-Y--------------------------SQGDDF--------------------PSKE-----FSQ----
OsPRR1/TOC1  267 --------PTTSSFD--SELQKGGNRLDSSD-HRGNFSSTTDRSDTGTDVNIRDKEAFEMPVQYPVVCFSSSNLHLERSNEGQND-A-SGTPPVY--HFPFYYPG--------MMDHGMT
SbPRR1/TOC1  273 --------PTTSSLD--SELQRGGSQLDSLD-NQGNCSSATDRSDTVTDVNIRNKEAFEMPVQYPMVCFSSSNTHMERSNEGHND-T-SGTPPVY--HFPFYYPG--------MVEHNMA
AtPRR3       404 --NVESCSPHDSPIA--KLLGS------SSS-SDN----PLKQQSSG-------------------------------------------------------------------------
AtPRR7       479 --NLGSTSLQDNNSQDLIKKTEAAYDCHSNMNESL----PHNHRSHVGSNNF--------------------------DMSSTTENNAFTKPGAP--KVSSAGSSSVK---------HSS
CpPRR3       471 --NIGSCSPLDNGSV--AMKTDMMHNFPSHS-NST----PLNQQSNGSS-NN--------------------------DMASTAK-CVTPKSEAF--SDKSESISAFK-----PF-HSSA
CpPRR7       487 --DIGSCSPPDNSLE--VAKTESEQNIQSNSN----------QHSNSDSNNN--------------------------DMGSTTN-NIFNQSIVF--SDKLAPISKDN-----CL-PPSA
PtPRR7a      409 --NVGSCSPLDNSSE--AAKTESMQNLQSNS-NST----PPNQRSNGSSHNN--------------------------DMGSTNN-ITFAKPSVI--SDKPTLKPTVK-----CH-YPSA
PtPRR7b      490 --NVGSCSLLDKCSE--AAKTESMQNLQSNS-NST----PRNLCSNGSSNNN--------------------------DVGTTTN-NAFAKPLVI--RDKPTPKSTVK-----CLHPSSA
VvPRR3       480 --NVGSCSPLDNSSV--AMKTETMHNFPSHV-DGI----PPNQQSNGSSNNI--------------------------DMVSTTK-YAIPKSEAY--NEKSESTSAFK-----CF-HSSA
VvPRR7       487 --NVGSCSPLDNSSE--AAKTESMQNFQSNS-NGT----PPNQ---------------------------------------------------------------IK-----HLQ-HSA
OsPRR37      456 --FMGSCSLHDNSSE--AMKTDSAYNMKSNS-DAA----PIKQGSNGSSNNN--------------------------DMGSTTK-NVVTKPSTN--KERVMSPSAVK-----ANGHTSA
OsPRR73      484 --FGGSCSPQDNSSE--ALKTDSNCKVKSNS-DAA----EIKQGSNGSSNNN--------------------------DMGSSTK-NAITKPSSN--RGKVISPSAVK-----ATQHTSA
SbPRR73      485 --CGESSSPQDNSSE--AVKTDSTCKMKSNS-DAA----PIKQGSNGSS-NN--------------------------DVGSSTK-NVIAKPSAN--RERVTSPSAIK-----STQHASA
AtPRR5       447 --SPVVTDQRKNVAASQ-DDNIV-LMNQYNTS-------EPP-PNA------------PRRN-DTSFYTGADSP-----GPPFSNQLN-SWPGQS--SYPTPTPINNIQFRDPNTAYTSA
AtPRR9       301 -------------------------------------------------------------A-VVALEESTSGE---------PKTPTE-SHEK----------------------LRKV
CpPRR5       360 --SPSVSNQRKESGNIY-EKNLSDLVGGFNCN-------APG-PPS------------TRTSLTIWQSDCQSKQ---------SEHATTSWPQQR--VFPVPIPVKSIKVNSPNTGYGSG
CpPRR9l      432 --VVGSCTESKEKASKT-HEPS-SYKLSQDSV-------RSSDPLGAPSG--------GSQG----NIIGQSRD---------I----------G--L----VSVSGVTFDSTCAEQIHV
PtPRR5a      428 --LESTGNQ-KELGTNY-DRKISST--GYNSD-------ALSLAPS------------TQKS-EISLAAGQTKE---------SEIATS-SPGQR--VFPIQIPAKETRLNNLCNSYGSV
PtPRR5b      417 --LANTGNQ-EEFRANY-DGKISSNVNGYNSD-------ALSLAPS------------TRRS-AISLAAGQTKE---------YEIVTS-SSGEK--VFPIHIPVKDTRFNNLCNSYGAV
PtPRR9la     418 --SASNGSDSKEEASKS-PDPS-SNQLAQNVG-------TLSQIHDASLS--------GNQEIITTPVIGQSGK---------VELAHP-SPQLG--L----IPVLGTRLDNISTGCGHV
PtPRR9lb     421 --SASNGSDSKEEASKS-PELS-SNQHAQNIN-------SISQRHGATLS--------GNQDM-TIPIIGQSGK---------AELAYP-SPRHG--L----IPVRRGMLDNISTEYGHD
VvPRR5       399 --STGVFNQQKNFGADS-DKRLSQLVTGYNSD-------------------------------IT-------------------------------------IPVRGVRFDAR-------
VvPRR9l      361 --SAINCSELKEDASYS-HERL-FNQLPESTV-------G--------------------------------------------------------------------------------
OsPRR59      413 --FPPSF---RVVHQ--QPVYDKNP--QSSR-VLL-----SCEHNTRESTVQAQVPLDRSTEGAAILCSSSVREDAGTSSSSPRK-DSLTHPSYG--FIPVPIPVG----AAIPYHYGAI
OsPRR95      385 --DAQAC-----------------------------------------STSATHIDLEN--------------KNGDSKTPSQDK-RETNQPPIR--VVPFPVPVGGLTFDGQPFWNGAP
SbPRR59      414 --FFPSL---CITGQ--EHVHGKDPVFQPNG-VLL----PPNEHNTGESTRQTRITLDSSPEGAAIMCSSSAREDAGVSSSSHRK-DSMSHPSYG--FIPVPIPVG----PGMPYHYGAI
SbPRR95      384 --DGQLC-----------------------------------------STSETLMDVEN--------------KNGDSAAPSQDM-TETNRPPIR--VVPFPVPVQGLTFDGQPFWNGTP

AtPRR1/TOC1  410 -RNYQEGNMNIPQVAMNRSKDSSQ--------------VDGSGFSAPNAYPYYMHGVMNQVMMQSAA------------------MMPQYGHQIPHCQPNH-----------------PN
PtPRR1/TOC1  335 -RNFKDD-----RVLMHQT-NEPQ--------------LDASSLSTQSVYPYFMSGVVNQVMMSSSAQLYQKNLHELQSL-GTSAMLPGYNH-LPQCPPH------------------MS
VvPRR1/TOC1  330 -------------VPIHPR-NEHQ--------------VDISGFPGHTAYPYCMSGVMNQVMMPSSAQLYPKSLHDMQNNATTSAMLPQYNH-LPQCPPH------------------VP
OsPRR1/TOC1  364 HPPVQN-FQGN----------------INN-AQVHTPQTLLPQYN-----------------------------------------------VYPQCHGVSMMPP---------------
SbPRR1/TOC1  370 VSSVQN-FQAN----------------INN-AQAHTPPTMLPQYN-----------------------------------------------VYPQCHGLPMISS---------------
AtPRR3           ------------------------------------------------------------------------------------------------------------------------
AtPRR7       556 FQPLPC--------------DHH--------------------------------------NNHAS---------------YNLVHVAERKK-LPPQCGSSNVYNETIEGNNNTVNYSVN
CpPRR3       546 FQPVQTGCISSSQQFLTENASDVGVNTVQA-QVRGSNKQVQLQVHHHHHHYHHHVHNMQQCQPQAD---------------HDDILLTKMAA-DARQCGSSNAFEGSMEEN--VVNYSVN
CpPRR7       558 LHPMQNGHFSPVEPVLEGKSDAAVPKAMLA-QGKSVDQLIQVH-HYHHHHHQYYIHNVPQQQYLAN---------------HDDTPLNNMVE-AASQCEACNSSGAYNDGS--DCAHAFS
PtPRR7a      485 FQPVQNDHTALPQPVIQGKGDAPIANTTLV-KSRGVNQQGQVQ--HHN----HCVHNMPQQQQLTN---------------HDDLSL-NMTA-AAPQCGSSNMLSTPTQGN--AGDYSLN
PtPRR7b      567 FQPVQNDQTLHAQPVIQGKGDAPIANTILA-QSRGMNQQGQVQ--HHR----HCVHNMP----LTI---------------RNDLSLKNMAA-AGPRFGSSNMLSTPMEGN--AGNYSMN
VvPRR3       556 FQPVTSGRMFPPQKVSSGKADDVE-------------------------------------QQQTD---------------NDDLFLKNMAA-AAPQCGSSNVIGGPTEGN--AGNYSVN
VvPRR7       529 FQPVQNTIL----------ADFANANTILA-HPSAMPPQVQIQ--NHHYYHHHVHNISQQQ-IRI----------------HDDLALTNMAK-SAPQCGSSNVLNAPVEGY--ACNHSLN
OsPRR37      533 FHPAQH-WTSPANTTGKEKTDEVANNAAKR-AQPGEVQSNLV---QHPRPILHYVHFDVSRE--------------------------NGGS-GAPQCGSSNVFDPPVEGH--AANYGVN
OsPRR73      561 FHPVQR-QTSPANVVGKDKVDEGIANGVNV-GHPVDVQNSFM---QHHHHVHYYVHVMTQQQQ---------------------QPSIERGS-SDAQCGSSNVFDPPIEGH--AANYSVN
SbPRR73      561 FHTIQN-QTSPANLVGKDKADEGISNAVKM-SHPTEVPQSCV---QHHHHVHYYLHVMTQKQS-----------------------SIDRGS-SDVQCGSSNVFDPPVEGH--AANYSVN
AtPRR5       534 MAP------ASLS-------P---SPSSVSP-HEYSSMFHPF---NSKPEG-------------------------------------------LQDRDCSMD---VDERR-YVSSATEH
AtPRR9       327 TSDQ-------------------GSATTSSN-QE--------------NIGSSSVSFRNQVLQ---------------------STVTN------QKQDSPIP--VESNRE-KAASKEVE
CpPRR5       446 IPPIFG-RQSTPS-------P-TLSPSSASQ-PELTSRANTF---CWPGFGNSKCEQ-MYLYD---------------------GMGQNVNS-ANQFMYNKLE--SLEDRG-HISPATVQ
CpPRR9l      504 STPIFY-SQSGLP-------PQGWNAKPAFLKEQSPFPMSTS---VHSDSYTHDAEQGYHLCD---------------------ETIGYSVD-QAVPVHSTLE--TVRELK-QGSPAAGQ
PtPRR5a      510 FPPIFC-KQSGLS-------P-MMSPSSACQ-QEPTYKVNQF---QHSNHG-STSEQ-NRLGQ---------------------HTNDSTNG-SLQKQEDRLD--SLEDRG-LISPATDQ
PtPRR5b      501 LPPM-------------------MSQSSASQ-KEPIHKVNPF---QCSNYGSTSVQLCDRLGQ---------------------NANDSING-SLQKQENKLD--SLEGRE-HISSATDQ
PtPRR9la     503 FSPLCY-TQSN----------AAWNPNLAG-RQQSPFPTTAS---VHSNPEVLDSKQNHKC----------------------------YVD-QNDLQQNNRE--PVDEMR-HDSPAAGQ
PtPRR9lb     505 FSPLYY-TQSS----------AAWSPKLAG-WQQSPYPLSTS---IHSNPDIHDSEKNHRCSD---------------------ETTYNSVD-QNDHQQNNKG--PVDEVR-HDSPAAGQ
VvPRR5           ------------------------------------------------------------------------------------------------------------------------
VvPRR9l      390 ---------------------------------------------------------------------------------------------------------------------TSE
OsPRR59      511 MQPMYY-PQGA-----------FMHCDSAA-INKTAIQHVSC---QSNYHENLGKPPQIDEHK---------------------QPEENHQL-HHSRQILRESGEPVDLAK--AHMERIN
OsPRR95      445 VASLFY-PQSA---------PPIWNSKTST-WQDATTQAISL---QQNGPKDTDTKQVENVEE---------------------QTARSHLS-ANRKHLRIEI--PTDEPR---HVSPTT
SbPRR59      515 LQPVYY-PQGP-----------LMHCDSAG-INKAAIQHGSG---QSNYHEAPGKPSQDDEHK---------------------QSEENHQL-HHSRQILRESGEPIEMAR--AHMDRAN
SbPRR95      444 MASLFY-PQSA---------PPIWNSKTSM-WQESTQATSLPQKSQQNEPNEMGAKPVENAEE---------------------QFVTGPPS-ASGKQLCVEV--PKDDPR---HISPMT
```

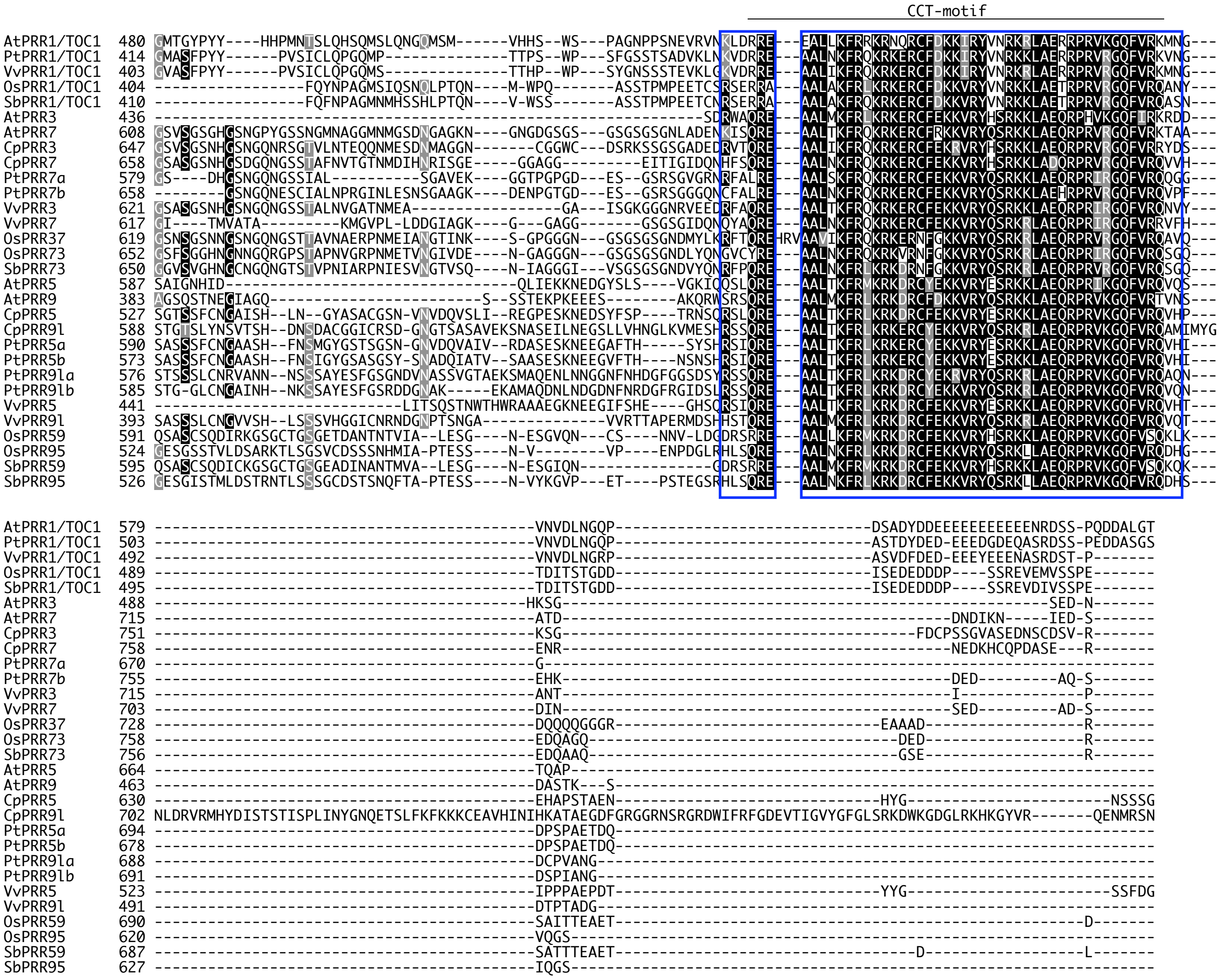

Additional file 3, Takata et al.
