# Supplementary material for: Phylogenetic footprint of the plant clock system in angiosperms: evolutionary processes of Pseudo-Response Regulators
Source: BMC Evol Biol. 2010 May 1;10:126. doi: 10.1186/1471-2148-10-126 (PMC2887406; doi:10.1186/1471-2148-10-126)
Supplement: Additional file 4 — Alignment of the amino acid sequences encoded by (A)AtPRR9s and (B)PtPRR5s. Sequence similarity is indicated below the alignment using the symbols "asterisk," "colon," and "dot" for identical, highly similar, and weakly similar residues, respectively. Black shadings indicate the PR-domain and the CCT-motif. Accession numbers and gene IDs of the PRR genes are shown in Additional file 1. [file 1471-2148-10-126-S4.PDF]

A

```
                                                     PR-domain
AtPRR9    1 MGEIVVLSSDDGMETIKNRVKSSEVVQWEKYLPKTVLRVLLVESDYSTRQIITALLRKCC
AtPRR9b     ------------------------------------------------------------

AtPRR9   61 YKVVAVSDGLAAWEVLKEKSHNIDLILTELDLPSISGFALLALVMEHEACKNIPVIMMSS
AtPRR9b     ------------------------------------------------------------

AtPRR9  121 QDSIKMVLKCMLRGAADYLIKPMRKNELKNLWQHVWRRLTLRDDPTAHAQSLPASQHNLE
AtPRR9b   1 -----MYLTC--------------------------------------------------
                 * *.*

AtPRR9  181 DTDETCEDSRYHSDQGSGAQAINYNGHNKLMENGKSVDERDEFKETFDVTMDLIGGIDKR
AtPRR9b     ------------------------------------------------------------

AtPRR9  241 PDSIYKDKSRDECVGPELGLSLKRSCSVSFENQDESKHQKLSLSDASAFSRFEESKSAEK
AtPRR9b   6 --------------------------------------------------RFEESKSAEK
                                                              **********

AtPRR9  301 AVVALEESTSGEPKTPTESHEKLRKVTSDQGSATTSSNQENIGSSSVSFRNQVLQSTVTN
AtPRR9b  16 AVVALEESTSGEPKTPTESHEKLRKVRSDQGSSTTSSNQENIGSSSVSFRNQVLQSTVTN
            ************************** *****:***************************

AtPRR9  361 QKQDSPIPVESNREKAASKEVEAGSQSTNEGIAGQSSSTEKPKEEESAKQRWSRSQREAA
AtPRR9b  76 QKQDSPIPVESNREKAASKEVEAGSQSTNEGIAGQSSSTEKPKEEESAKQRWSRSQREAA
            ************************************************************
             CCT-motif
AtPRR9  421 LMKFRLKRKDRCFDKKVRYQSRKKLAEQRPRVKGQFVRTVNSDASTKS
AtPRR9b 136 LMKFRLKRKDRCFDKKVRYQSRKKLAEQRPRVKGQFVRTVNSDASTKS
            ************************************************
```

B

```
PtPRR5b   1 MGVVVVSSGEELEVKTGSETEEEKQSKEETESETGEVKRKRKKKEGEGSDNGLVRWERFL
PtPRR5c   1 -----------MEVE---------------------------------------------
                       :**:
                                          PR-domain
PtPRR5b  61 PRMVLRVLLVEADDSTRQIIAALLRKCSYKAVATVSDGLKAWEILKERPHNIDLILTEVD
PtPRR5c   5 --------------------------------------------LKEMLNDLDYLKKSLS
                                                        ***  :::* : ..:.

PtPRR5b 121 LPSVSGYALLTLIMEHEICKNIPVIMMSSQDSIKTVYKCMLRGAADYLVKPIRKNELRNL
PtPRR5c  21 NPS---------------------------------------------------------
             **

PtPRR5b 181 WQHVWRKQSSLGGGNGPHDESVGQDKTEATSENNADGNHSSGEMASIQRSKEQAVKRSDS
PtPRR5c  24 -----------------------------------------------------NLASSFH
                                                                  :  *

PtPRR5b 241 QSSCTKPGLEAEGAHMENMQEFLQPVWSKFSLTDTNMQKHEEHVNLGQKLLVRDSEAEGS
PtPRR5c  31 KSSCTKPGLEAEGAHMENMQEFLQPVWSKFSLTDTNMQKHEEHVNLGQKLLVRDSEAEGS
            :***********************************************************

PtPRR5b 301 ATAVCEDSNKITVDKEITPGSGRVTANIAIEGCDKIGALANSPREAIDFMGASTNHSSFN
PtPRR5c  91 ATAVCEDSNKITVDKEITPGSGRVTANIAIEGCDKIGALANSPREAIDFMGASTNHSSFN
            ************************************************************

PtPRR5b 361 NVEIHFCSSPHLDLSLRRSHPSGFETQVTEERHTLRHSNASAFTWYTNRASQLPHSALAN
PtPRR5c 151 NVEIHFCSSPHLDLSLRRSHPSGFETQVTEERHTLRHSNASAFTWYTNRASQLPHSALAN
            ************************************************************

PtPRR5b 421 TGNQEEFRANYDGKISSNVNGYNSDALSLAPSTRRSAISLAAGQTKEYEIVTSSSGEKVF
PtPRR5c 211 TGNQEEFRANYDGKISSNVNGYNSDALSLAPSTRRSAISLAAGQTKEYEIVTSSSGEKVF
            ************************************************************

PtPRR5b 481 PIHIPVKDTRFNNLCNSYGAVLPPMMSQSSASQKEPIHKVNPFQCSNYGSTSVQLCDRLG
PtPRR5c 271 PIHIPVKDTRFNNLCNSYGAVLPPMMSQSSASQKEPIHKVNPFQCSNYGSTSVQLCDRLG
            ************************************************************

PtPRR5b 541 QNANDSINGSLQKQENKLDSLEGREHISSATDQSASSSFCNGAASHFNSIGYGSASGSYS
PtPRR5c 331 QNANDSINGSLQKQENKLDSLEGREHISSATDQSASSSFCNGAASHFNSIGYGSASGSYS
            ************************************************************
                                                          CCT-motif
PtPRR5b 601 NADQIATVSAASESKNEEGVFTHNSNSHRSIQREAALTKFRLKRKERCYEKKVRYESRKK
PtPRR5c 391 NADQIATVSAASESKNEEGVFTHNSNSHRSIQREAALTKFRLKRKERCYEKKVRYESRKK
            ************************************************************

PtPRR5b 661 LAEQRPRVKGQFVRQVHIDPSPAETDQ
PtPRR5c 451 LAEQRPRVKGQFVRQVHIDPSPAETDQ
            ***************************
```

Additional file 4, Takata et al.
