# Supplementary figures and images for: Phylogenetic footprint of the plant clock system in angiosperms: evolutionary processes of Pseudo-Response Regulators
Source: BMC Evol Biol. 2010 May 1;10:126. doi: 10.1186/1471-2148-10-126 (PMC2887406; doi:10.1186/1471-2148-10-126)

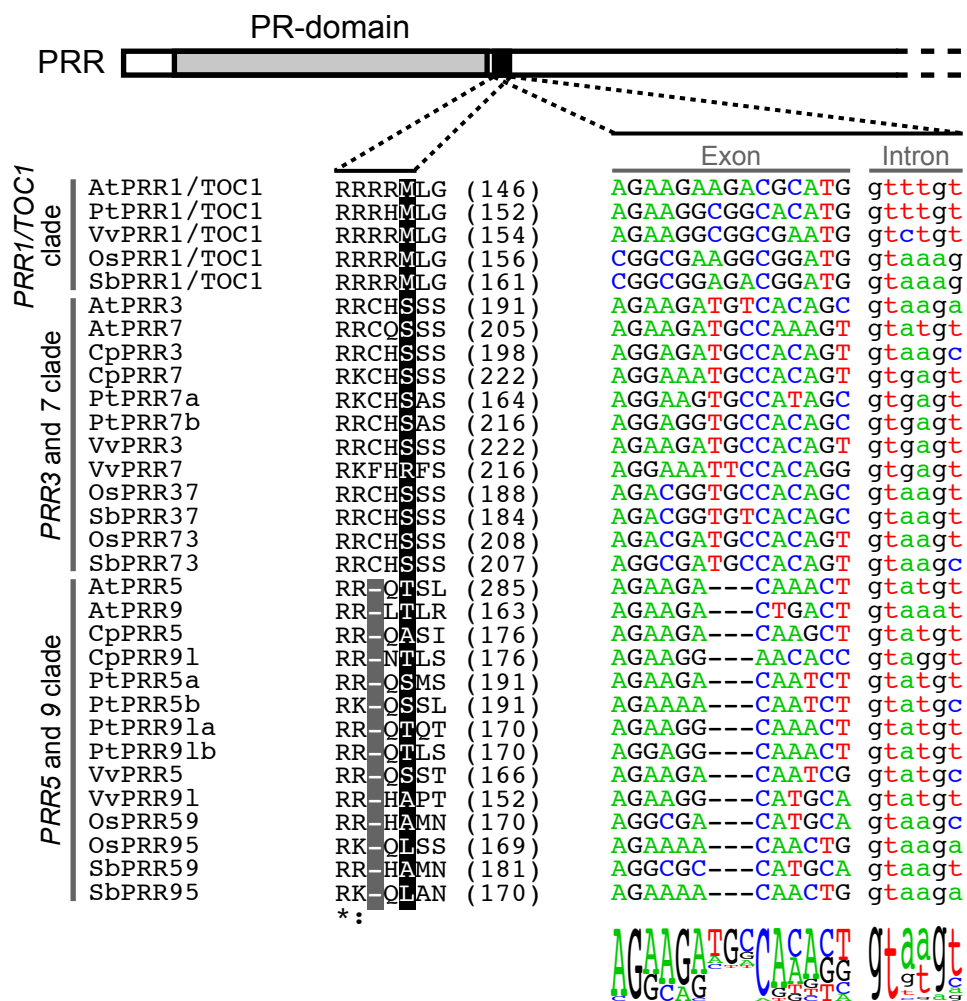

Additional file 5, Takata et al.

Supplement: Additional file 5 — Nucleotide sequences around the region of the exon-intron boundaries of angiosperm PRR genes at the flanking region of PR-domain. Black and gray shadings on the alignments indicate a site of exon-intron boundary and one-amino acid deletion, respectively. Higher degree of conservation of nucleotide sequence is shown by the bigger size of letters. [file 1471-2148-10-126-S5.PDF]

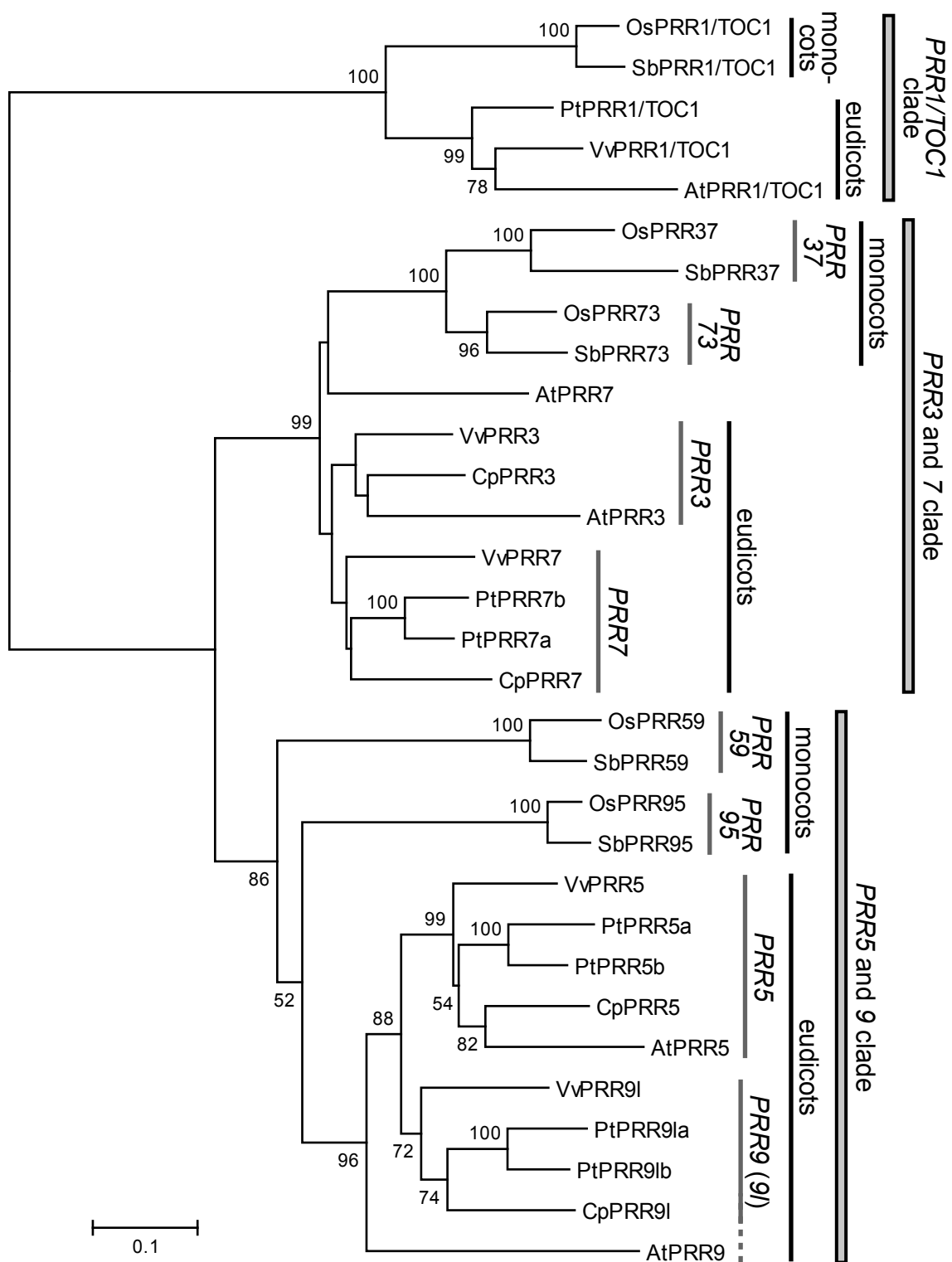

Additional file 6, Takata et al.

Supplement: Additional file 6 — Phylogenetic tree of PRR genes reconstructed by the Neighbor-Joining (NJ) method. Full-length amino acid sequences were aligned using TCoffee program. The phylogenetic tree was reconstructed by the NJ method from the numbers of amino acid substitutions estimated by applying the JTT model. The numerals at the branch indicate bootstrap values calculated by the NJ method with 1,000 replications. Bootstrap values >50% are shown. [file 1471-2148-10-126-S6.PDF]

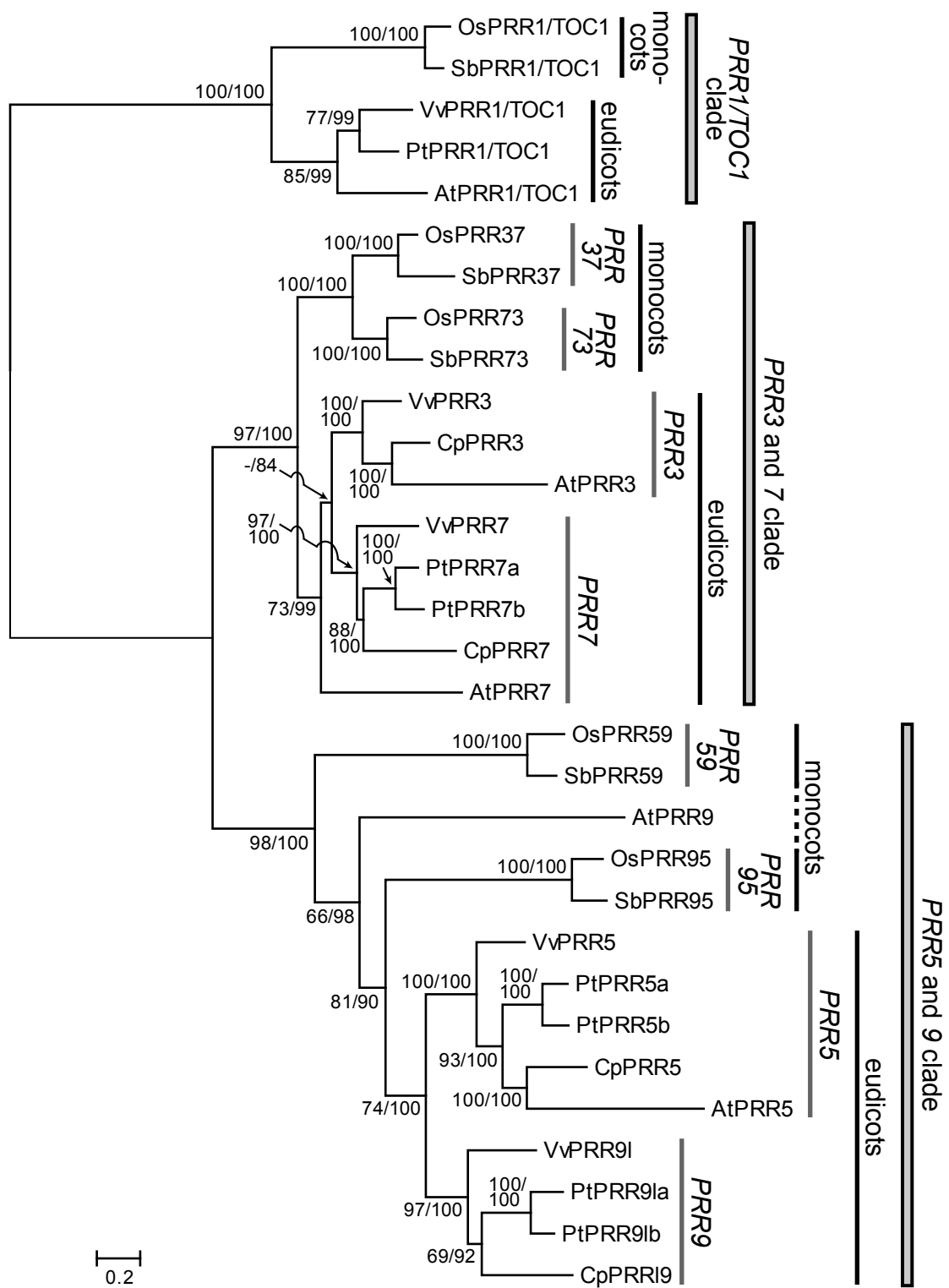

Additional file 7, Takata et al.

Supplement: Additional file 7 — Phylogenetic trees of PRR genes reconstructed by the Maximum likelihood (ML) and Bayesian methods. Full-length amino acid sequences were aligned using TCoffee program. The phylogenetic trees were reconstructed by the ML and Bayesian methods with applying the JTT model. The ML and Bayesian analyses were performed using the PhyML http://www.atgc-montpellier.fr/ and MrBayes http://mrbayes.csit.fsu.edu/ programs, respectively. PRR1/TOC1 genes were utilized as an outgroup in the phylogenetic trees. Support for the branches was calculated as percent of 100 bootstrap replications of the ML method (left) and Baysian posterior probabilities (right). The values >50% are shown. [file 1471-2148-10-126-S7.PDF]
